# The restoration and erection of the world’s first elevated obelisk

**DOI:** 10.1038/s41598-023-29092-z

**Published:** 2023-02-04

**Authors:** Atef M. Saleh, Sherif A. Mourad, Hazem H. Elanwar, Omar K. Metwally, Eissa Zeidan, Mahmoud A. Adam, Mostafa F. Ameen, Khalid R. Helal, Mohamed S. Sholqamy, Hussien E. Allam, Mohamed A. Ismael, Khaled A. Mostafa, Hany M. Helal, Amr Y. Elbanhawy, Christian U. Grosse, Mourad M. Bakhoum, Mousa M. Farag, Hani B. Matar, Hanan H. Eltobgy, Moustafa I. Moharram, Mohamed M. Marzouk, Mahmoud S. Metawie, Mohamed R. Ali, Ashraf N. Sayed, Mohamed G. Mohamed, Mohamed M. Elkarmoty

**Affiliations:** 1 Director General of the Grand Egyptian Museum Project and the development of the surrounding area, Giza, Egypt; 2 Cairo University, Al Gamma St., Giza, Egypt; 3 Grand Egyptian Museum, Giza, Egypt; 4 College of Archaeology and Cultural Heritage, The Arab Academy for Science, Technology and Maritime Transport, Cairo, Egypt; 5 Ain Shams University, Kasr El-Zaafanana, Abbaseya, Cairo, Egypt; 6 Technical University of Munich, Munich, Germany; 7 ACE Consultants, Mohandessin, Giza, Egypt; 8 Banha University, Faculty of Engineering, Shoubra, Cairo, Egypt

**Keywords:** Engineering, Materials science

## Abstract

Obelisks presented an important element in the architecture of ancient Egypt. This research is concerned with the re-erection of an obelisk that belongs to the famous Pharoah Ramses II. It was found broken and was transported to the Grand Egyptian Museum for restoration and display. An observation of Ramses II Cartouche at the bottom side of the obelisk base inspired the authorities to provide an innovative architectural design to display the obelisk elevated. The supporting structure was designed to allow the visitors to walk underneath the obelisk and observe Ramses II's signature. The idea of elevating the obelisk presented several challenges including evaluating the obelisk's current condition, restoration and fixation methodology, structural stability, and uncertainties of material characteristics, amongst others. To control the obelisk deformations under lateral loading, state-of-the-art base isolators were introduced. For the task to be achieved, a multidisciplinary team including historians, conservators, archaeologists, architects, and engineers with different specialties was appointed. The team performed the task successfully and currently, the obelisk stands at the entrance piazza of the Grand Egyptian Museum representing the world’s first elevated obelisk.

## Introduction

**Figure 1:**
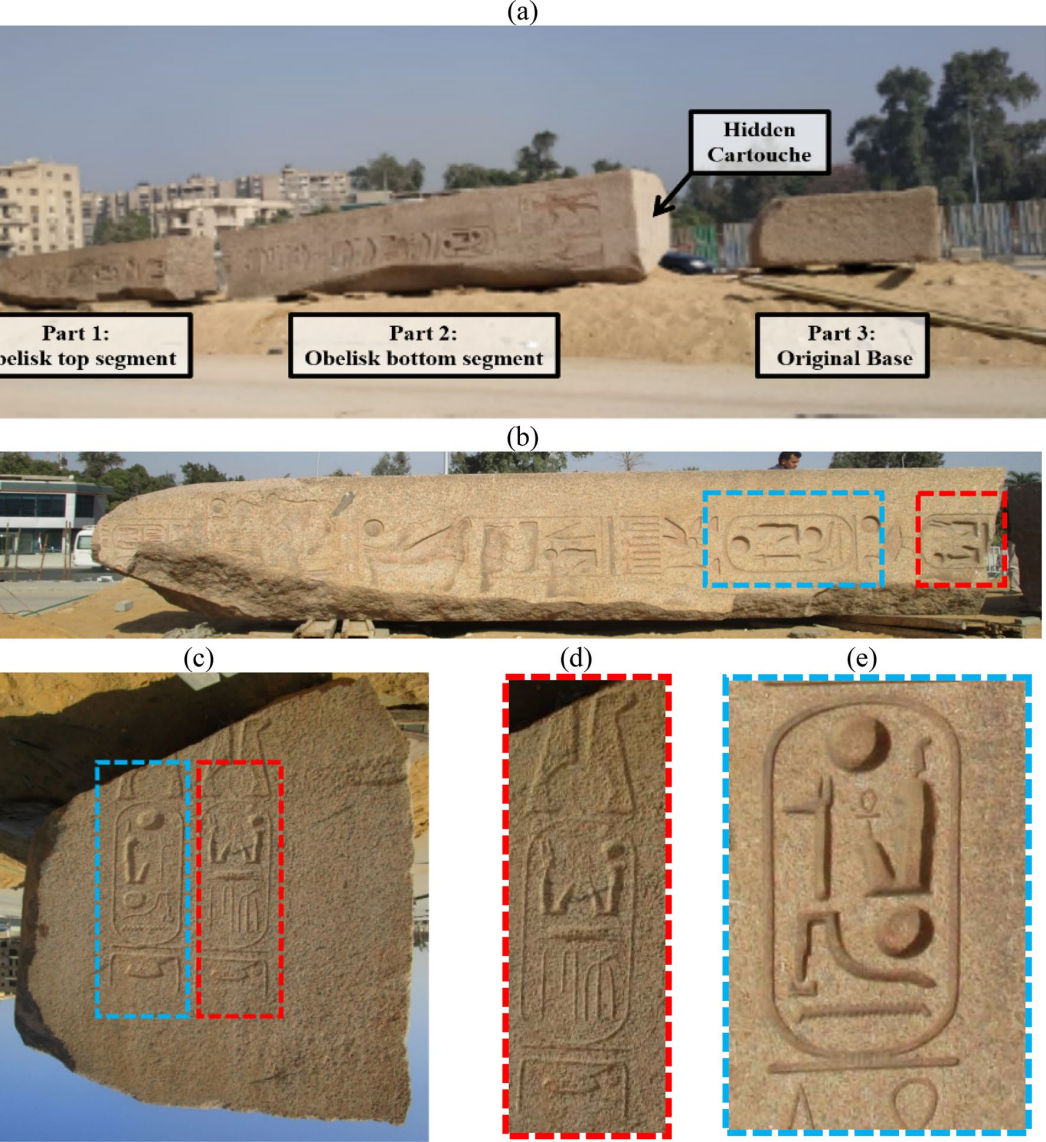
The Obelisk of Ramses II found at San Elhagar. (**a**) The obelisk was found broken into two pieces and separated from the base. (**b**) The top piece of the obelisk with hieroglyphic carvings. (**c**) The cartouche at the bottom face of the obelisk has the names of King Ramses II, which are the same carvings shown on the obelisk side. (**d**) carvings Birth name: Ra-ms-sw mry-Imn “Born is the God Ra, beloved of Amun”. (**e**) Name of the throne (patron surname): Wsr-mAat-Ra stp-n-Ra "Strong is the justice of Ra, the chosen one of Ra".

Their existence at the entrance of temples conveyed an aura of holiness and strength through their association with Gods and Pharaohs^1,2^. Currently, obelisks are showcased either in Egypt or in other countries all over the world^3–5^. A few are still at their original site, typically due to defects or damage^6–10^. King Ramses II is the third Pharoah of the 19^th^ family, who ruled ancient Egypt for nearly 66 years between (1279–1213 BC). He is considered by many scholars to be the greatest Pharaoh of all time, whose military achievements extended to the east, west, and south of Ancient Egypt. Ramses II even wrote the first peace treaty the world has ever recorded. In addition to the military achievements, his reign was distinctive for building new cities and great monuments including temples, statues, tombs, etc.^11,12^. Among the great monuments attributed to King Ramses II is the obelisk found in "Tanis" area, which is now known as "San Elhagar" in Sharkia Governorate, Egypt. The obelisk is about 16 m high with a tapered top and weighs 87 tons carved from pink granite. It was found broken into two pieces and separated from its base (Fig. 1a). The sides of the obelisk are carved in textual writing—which is evident in the vertical hieroglyphic line inscribed on each of the four sides of the obelisk. This is in addition to the bottom, which is also engraved with two cartouches containing the royal names and titles of King Ramses II, which are identical to those contained in the inscription lines on the sides of the obelisk body (Fig. 1b–e).

**Figure 2:**
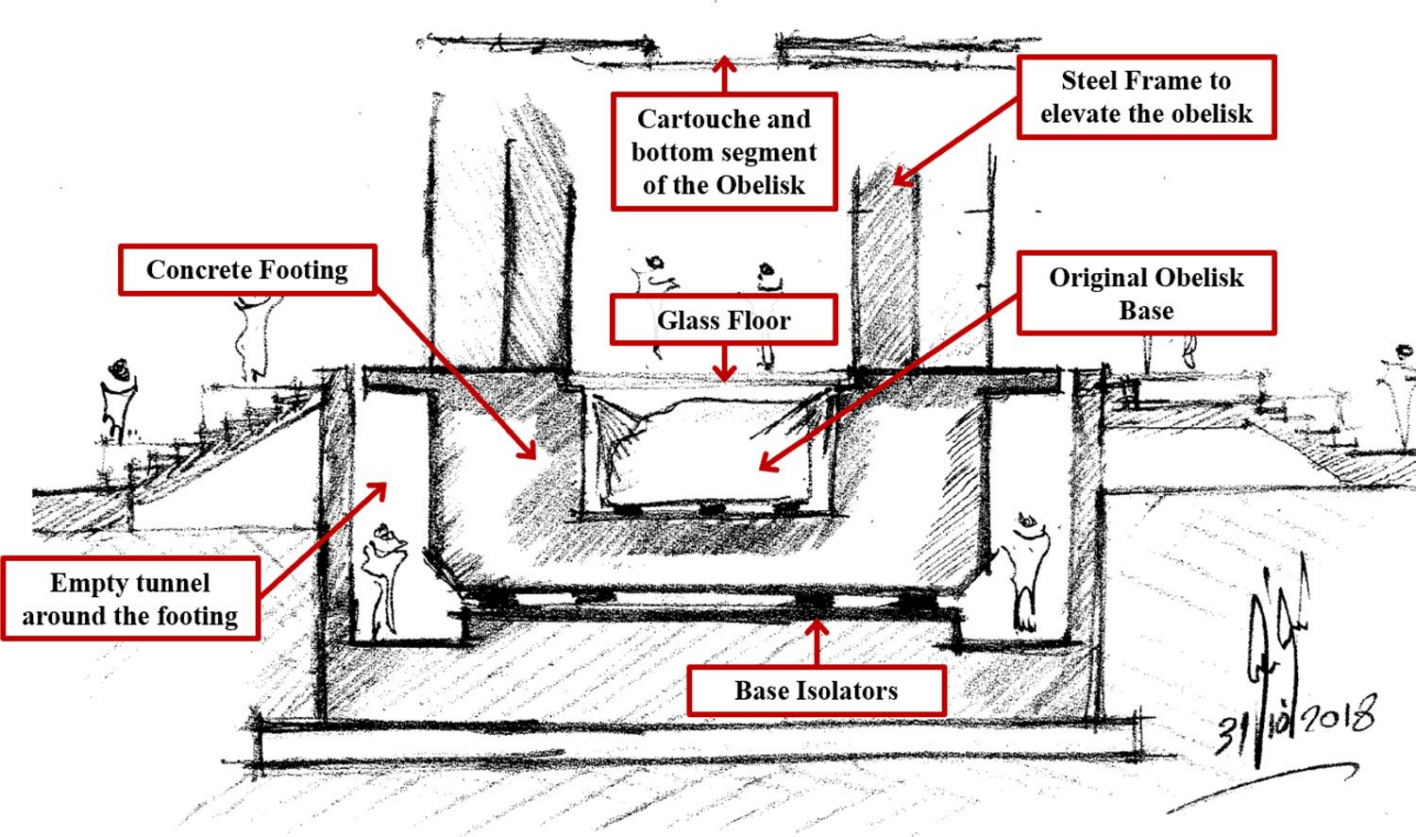
Schematic concept of the elevated obelisk by Atef M. Saleh.

Although the king’s Cartouche is meant to be hidden between the bottom of the obelisk and the granite base, it is believed that it was used by King Ramses II to preserve the origin of his obelisk from future manipulation. Whereby, less capable rulers could replace engraves on the side of the obelisk with their own, but not the one hidden at the base. This discovery inspired the idea of elevating San Elhagar Obelisk to be the first elevated obelisk in the world. Thus, allowing the visitors to observe the Cartouche that has been hidden for more than 3500 years. The architectural concept is shown in Fig. 2, where the obelisk is elevated on a steel frame so that the visitors can stand on a glass floor covering the original base, while the Cartouche at the base of the obelisk is visible above them. This design intends to give the visitors a feeling that they represent the continuity between the different parts of the obelisk. To emphasize the uniqueness of the obelisk, it is located at the outer foyer of the Grand Egyptian Museum, facing the Pyramids of Giza from one side and the museum entrance from another side. It is worth mentioning that the museum entrance is distinguished by a great statue of Ramses II about 11 m high, which would be facing the obelisk. However, the proposed design was associated with several engineering challenges. Elevating the obelisk introduced a new connection between the obelisk base and a steel frame, where stainless steel anchors were used avoiding the location of engravements on the sides and the bottom of the obelisk. Elevating the obelisk also required thorough investigation of the impact seismic forces, which resulted in the introduction of base isolator. In addition, an empty tunnel was introduced around the footing to allow for free lateral movement and to reduce the lateral soil pressure on the footing and can be used as well for base isolation maintenance (Fig. 2). Understanding of the obelisk material mechanical properties and its interaction with the anchor system imposed another challenge, which required performing multiple laboratory tests. In order to understand the internal structure and physico-mechanical properties of the obelisk material a series of non-destructive non-invasive techniques were implemented. To fulfill the proposed concept a multidisciplinary team including historians, conservators, archaeologists, architects, and engineers with different specialties was appointed. The tasks of the team included the following: (1) scanning and modeling the obelisk current condition, (2) restoration of the obelisk damaged parts and engraves, (3) performing field and laboratory material assessment to decide on the best anchoring approach, (4) to design the elevated frame and the associated base isolators, (5) to design the anchor connection between the elevated frame and the obelisk, in addition to the middle part of the obelisk connection, (6) erection method statement of the obelisk, and (7) to survey the obelisk after erection to ensure its verticality.

### Fine restoration

**Figure 3:**
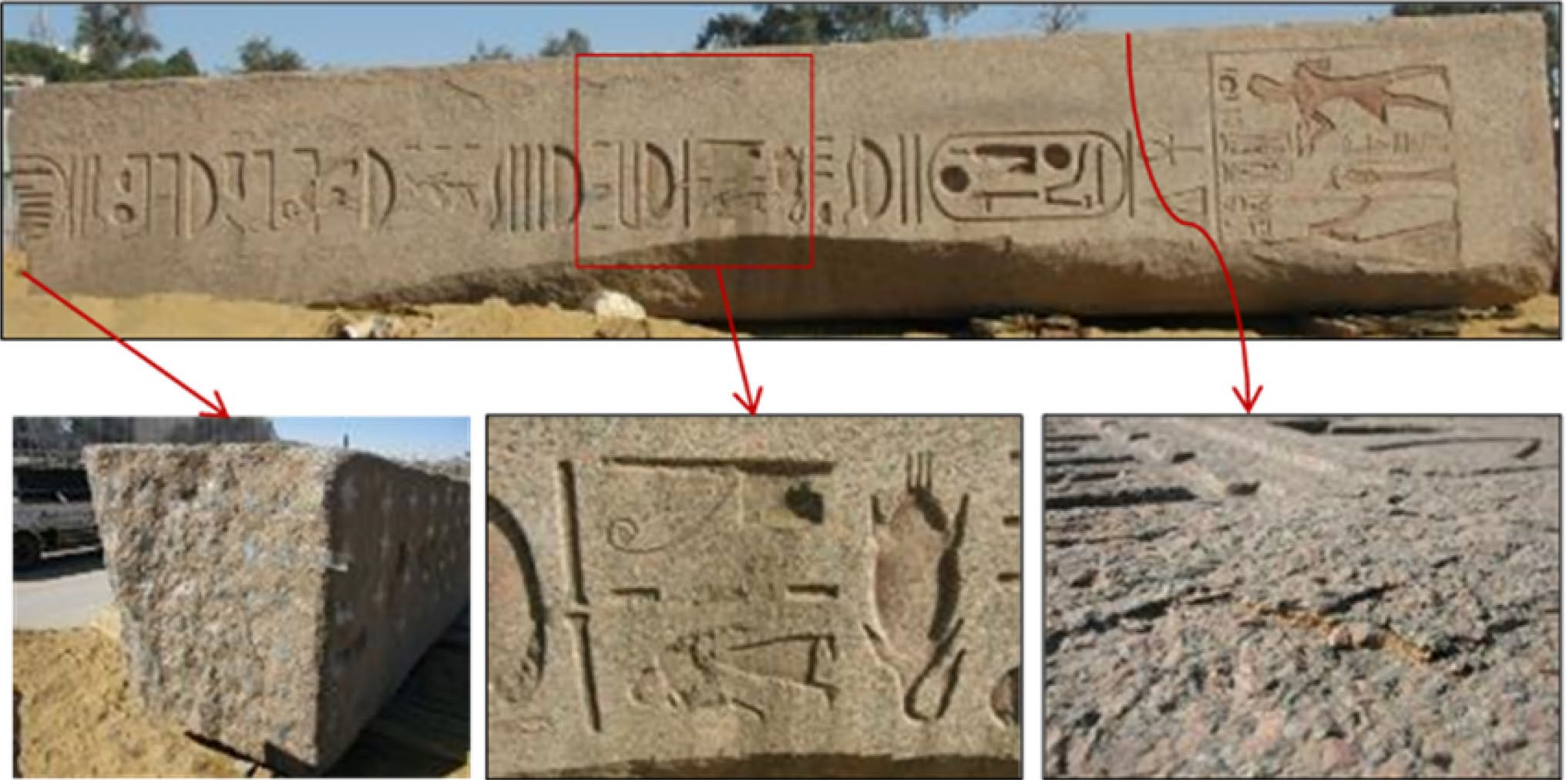
Areas of the obelisk that suffer from flaking, spotting, laminating, and transformation of feldspar minerals into clay minerals, as well as clay deposits that distort the appearance of the obelisk and their inscriptions.

All surfaces of the obelisk carried many stains, dust and dirt, which overlap in its fine pores, gaps and inscriptions as a result of the environment in which the obelisk was preserved, which obscured large part of its inscriptions, fine details, architectural and decorative features. Many parts of the granite obelisk were found covered with mud dirt, from the original site of the obelisk in Tanis "San Elhagar" area, where the obelisk was found. Damage to the granite obelisk with mud dirts, usually saturated with salts, resulted in discoloration spots, microfracturing, mineral alteration, salt crystallisation and granular disintegration as shown in Fig. 3. Cleaning is often one of the first steps to be undertaken to remove the dirt and prepare for subsequent restoration work^13^. It is well known that before applying cleaning and any restoration works, all documentations, examinations, tests, and analyses were carried out.

The restoration works caried out on the granite obelisk can be summarized in the following procedures:

- Cleaning process

**Figure 4:**
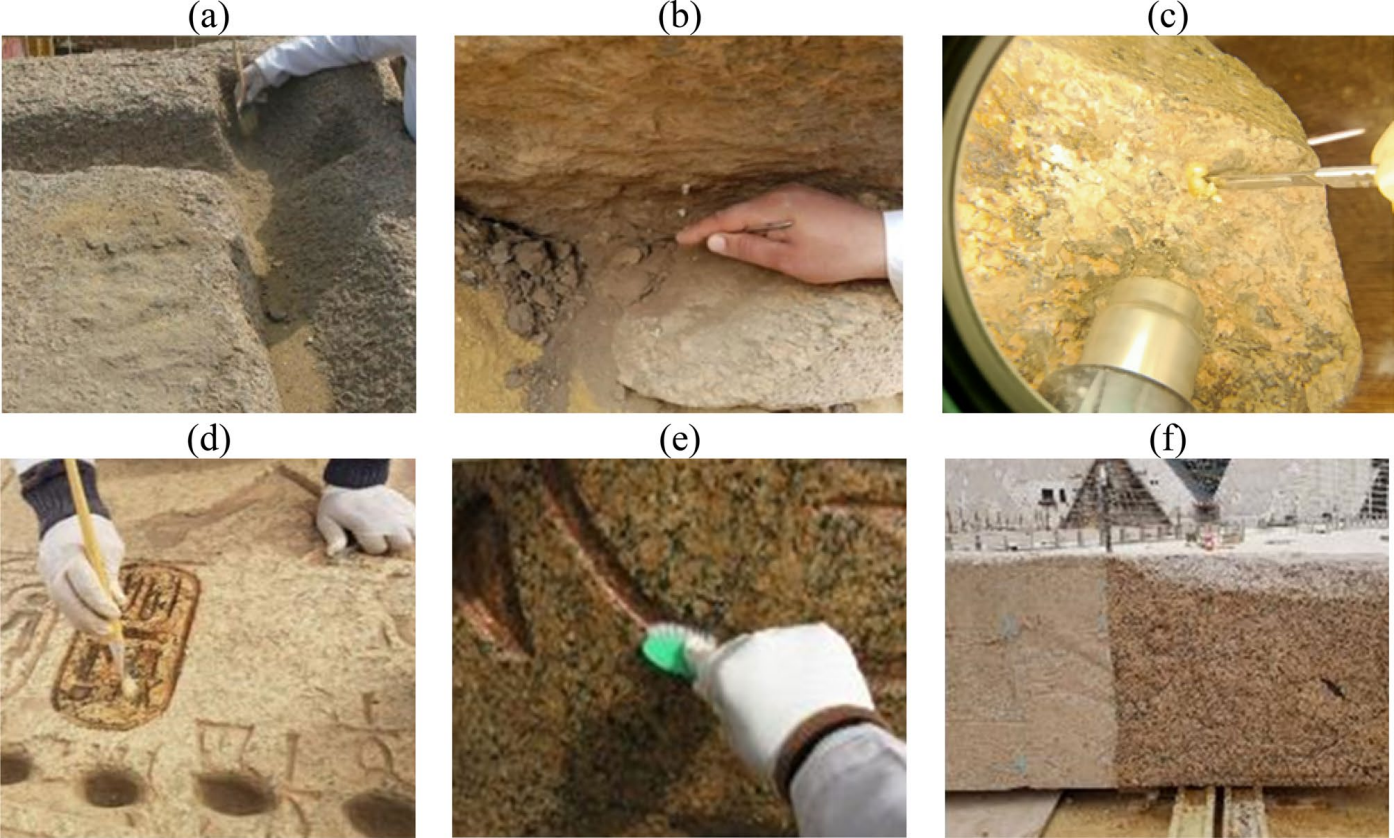
Procedures of mechanical and chemical cleaning (**a**) Removal of sand and mud deposits by brush, (**b**) Removal of sticky mud deposits by scalpel, (**c**) Removal of salt deposits by scalpel under eyepiece, (**d**) Chemical cleaning by a mixture consisting of distilled water and ethyl alcohol, (**e**) Washing the stone by distilled water, The brush indicates the red color of the royal cartouche of King Ramesses II (**f**) Showing a part of the obelisk before chemical cleaning, in the left, and a part of the obelisk after chemical cleaning, in the right.

Mechanical cleaning: A wide range of techniques are available for cleaning of stone monuments, but in the case of the studied granite obelisk, the researchers started carefully with the safe mechanical cleaning using soft and stiff bristle brushes, spatulas, scalpels and flushing out with distilled water to the finest stage. When the tools used can do no more cleaning, the next step, which is chemical cleaning, was introduced^14^. The mechanical cleaning procedures aimed at removing the dust that was firmly attached to the surfaces of the granite obelisk, and reducing the effects of staining and other deposits as shown in Fig. 4a–c.

Chemical cleaning: As a general rule, chemical intervention must be minimal, to ensure the long-term safety of the stone monument, and this is what was taken into account in the restoration implementations of the studied obelisk. The base of chemical cleaning relies on breaking the bonds between dirt constituents, using chemical solutions or organic solvents, and then dismantling and removing them easily. The chemical cleaning process of the obelisk was intervened to remove the highly sticky deposits, which the mechanical cleaning could not remove, and cause undesirable staining of the surface. Chemical cleaning procedures were based on the use of a mixture consisting of distilled water and ethyl alcohol in a ratio (1:1) by direct cleaning and poultices methods. Cleaning was usually carried out using wet swabs, and applied to the granite topically, and washed immediately with distilled water. Otherwise, poultices saturated with the cleaning solution were implemented directly to the dirty positions of granite for a few minutes then removed, and washing its place with distilled water^15^ as shown in Fig. 4d–f. The cleaning processes clarified the archaeological features of the granite obelisk, and showed the royal cartouches painted in red, which were hidden by the muddy dirts accumulated on them.

- Treatment of granular disintegration

Many parts of the obelisk were disjointed in the form of separated granules or powder due to many reasons, the most important of which is the thermal expansion and contraction that the surface of stone was exposed to for many years under harsh environmental conditions. Thermal changes, in fact, were not the only reason to cause severe disintegration of the granite obelisk, but also pollutants, wind, relative humidity, moisture, and groundwater represented important factors for granular disintegration of the granite obelisk. Granular disintegration is responsible for the loss of granite substance. The stone showed loss of hardness and polish leading up to mineral alterations. The textural anisotropy of the granite obelisk lead to the grow of cracks or microcracks, while progressing its physical degradation giving rise to planes of weakness. The disintegration of granite materials decay tends to be by exfoliation^16^.

**Figure 5:**
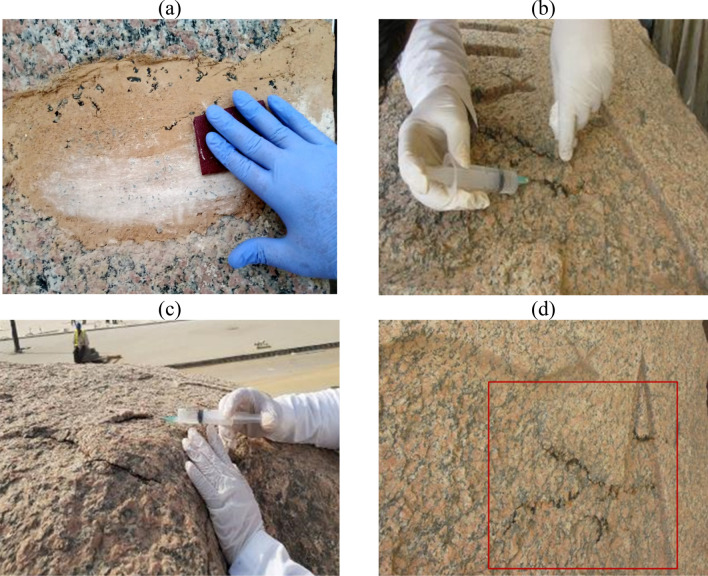
Treatment of granular disintegration and cementation process of separated crusts (**a**) Treatment of granular disintegration, (**b**) Cementing the crusts of small size, (**c**) Cementing the crusts of medium size, (**d**) Completion of the injection process using Paraloid B72 before total drying of the partially detached crusts.

The remedial conservation for such a manifestation relied on a local mechanical cleaning of the disintegrated positions, and then mixing the disintegrated granules with a solution prepared of Paraloid B72 dissolved in acetone at concentrations ranging from 5:15%, depending on the case of granules, and accurately re-gluing the granules in their positions^17^. Some inorganic natural oxides were used in a limited percentage to give some necessary color effects, after that fine and medium sandpaper were used to give the final touches as shown in Fig. 5a.

- Cementation of separated crusts

The damage of granitic stone substances is a complex process caused by the interaction between numerous correlated factors such as climatic conditions, atmospheric pollution, composition and properties of the material itself that finally result in physico-chemical, mechanical and biological deterioration. Moreover, there are some specific damaging forms affecting granitic monuments depending on the surrounding environmental conditions such as exfoliation or separated crusts due to aggressive alternative drying and wetting cycles^10^. Hydration phenomenon of granite-forming feldspar minerals in wet conditions results in the formation of clay minerals. This phenomenon severely affects granite monuments, causing their weakness and flaking. This is most likely what the granite obelisk was exposed to, which caused separation of some surface crusts, and required intervention to cement these crusts to restore the mechanical strength of the obelisk, and the following steps were intervened:

Cementing the crusts of small size: These crusts do not exceed 50 mm in size. The crusts were first cleaned of dirt and mud, then wetted with distilled water. After that, they were cemented using a solution of Paraloid-B72 dissolved in acetone in concentrations of 25:30%. It is worth mentioning that Paraloid-B72, is an acrylic copolymer made of polyethylmethacrylate (EMA)/methyl acrylate (MA) (70/30, respectively) ^18^.

Cementing the crusts of medium size: The sizes of these crusts range from 50:200 mm, The crusts were first cleaned of dirt and mud, then wetted with distilled water, after that, they were cemented using a solution of Paraloid-B72 dissolved in acetone in concentrations of 30:40%, supported by a small amount of Micro balloons. Micro balloons are not waterproof, but they are often preferred for their excellent sanding characteristics The technical specifications are as follows: (composition: Phenolic resin; appearance: red/brown powder; particle size: 50 microns; particle density: 250 g/litre; bulk density: 100 g/litre) ^19^. In all cases, syringes were used for injection and gluing of the separated crusts as shown in Fig. 5b–d.

Cementing the crusts of large size**:** The sizes of these crusts are relatively large, some of them weigh 400 kg and were found at the top of the obelisk. They were re-assembled, after cleaning and wetting procedures, by dowelling technique for the delaminating parts using stainless anchors with diameters ranging from 5:10 mm with Hilti—HIT-RE 500 V3 as an injection mortar, it is an irreversible epoxy resin, chemically safe and, if properly applied, does not cause any damage to the archaeological stone. The material has a high adhesive strength, and it consists of two parts; resin and hardener. The most important characteristic of this material is the chemical stability, it does not show any changes in volume due to the difference in temperature and humidity. Drying time is sufficient for application under different climatic conditions, there are many types of the material that differ in colors and drying time. It is worth noting that this method was used in the re-assembly of the main parts of the studied granite obelisk^20^.

- Injection and filling of cracks and gaps

**Figure 6:**
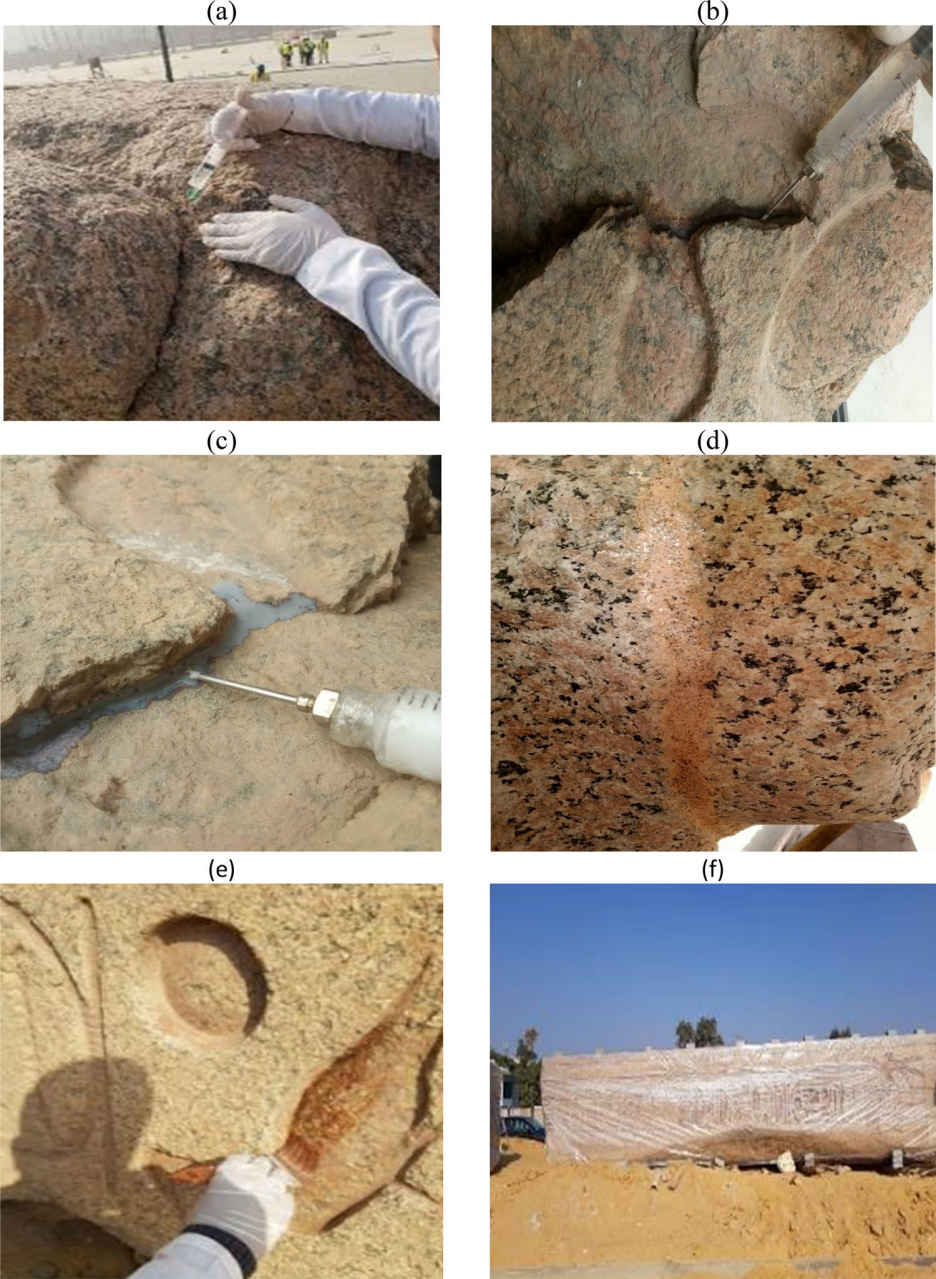
Steps of injection and filling of cracks and gaps, as well as consolidation of colored parts (**a**) Injection of cracks by Paraloid B 72, (**b**) Injection of wide fractures by Paraloid B 72 in high concentration, (**c**) During the application of Micro balloons, (**d**) Using a mortar consisting of granite powder, Micro balloons, Paraloid B72 and small percentage of natural inorganic oxide in order to filling the gabs, (**e**) Consolidation of the red colored parts, (**f**) Covering the obelisk with polyethylene after initial fine restoration, preparing for the assembly and erection processes.

The injection process was carried out for fine and relatively wide cracks in the granite obelisk. Moreover, gaps resulting from the various weathering processes were filled. This process including completion of the missing parts of granite with a mortar consisting of granite powder, Micro balloons, Paraloid B72 and small percentage of natural inorganic oxide^21^. In general, this stage of restoration works aimed at consolidating the weak parts of the obelisk, where the connectivity that micro-fissures provide in granite stone allows an easy impregnation with the consolidant, and significantly large depths can be fully consolidated^22^. Furthermore, Paraloid-B72 dissolved in acetone (3%) was applied to the red colored parts for consolidation and protection purposes in Fig. 6. As a future protective procedure, the researchers have developed Ethyl silicate/ZnO nanocomposite, in a similar study, which can be used for the self-protection of the obelisk, after erection and exposure to open air and pollutants. It may be applied by brush on the entire granite obelisk, almost every few years, in conjunction with the preventive conservation measures^23^.

### Obelisk 3D laser scanning and modeling

The 3D laser scanning to obtain 3D data about physical objects of different shapes and sizes is a cost and time effective technique. Laser scanners have been broadly utilized in different applications such as architectural, archeological, and deformation monitoring of structures^24–26^. The process of 3D laser scanning and modeling of the obelisk followed a procedure that consists of three phases: (1) site exploration and scanning planning, (2) data acquisition and processing, and (3) 3D modeling and properties extraction. The first phase (site exploration and scanning planning) is divided into two stages; the first one is the site exploration, and the second stage is the planning of the scanner locations. The site exploration stage involved an investigation tour to generate simple sketches, notes, videos, pictures, and initial measurements that would be integrated to have a complete plan for the site. The planning stage started after completing the site exploration stage. All information gathered in the previous stage was used in the planning task to determine the number of scans required to capture the features of the obelisk from all directions.

**Figure 7:**
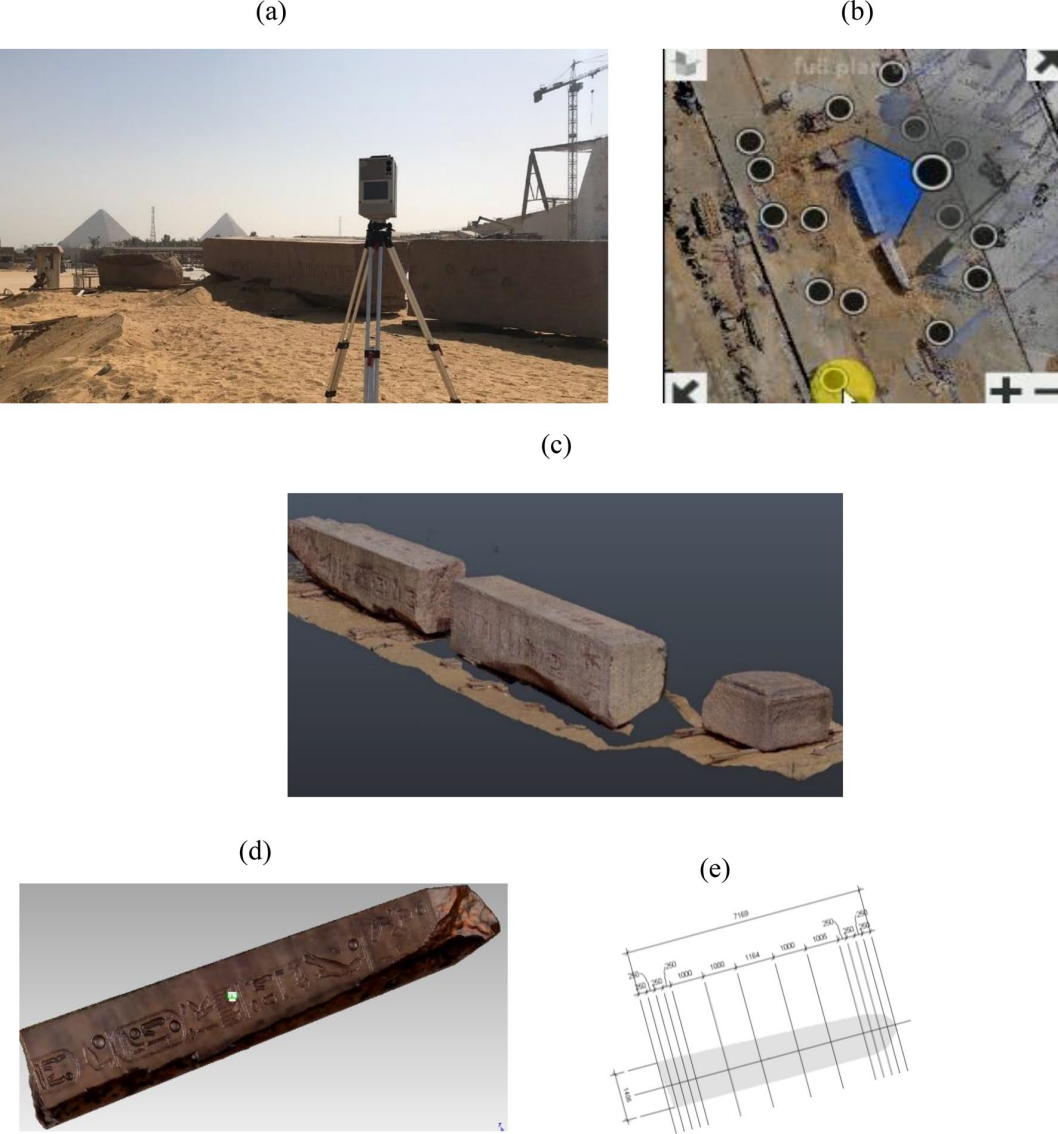
3D laser scanning and modeling of the obelisk. (**a**) Measurements using a Terrestrial Laser Scanner. (**b**) Processing scanned data. (**c**) Registering a point cloud of the obelisk. (**d**) Textured 3D models of the obelisk. (**e**) Extracting obelisk properties.

The second phase (data acquisition and processing) consisted of two steps. The first step involved the use of a Terrestrial Laser Scanner to scan and capture the texture of the obelisk according to the information gathered in the previous phase (Fig. 7a). As such, accurate documentation of the geometrical and texture features of the obelisk was obtained. A Z + F IMAGER® 5010C laser scanner was used to scan all the obelisk sides. A total of 16 laser scans were taken to cover all the obelisk sides (Fig. 7b). The scanned data was processed and a clean point cloud was registered to be used in the visualization and development of the 3D model. The processing of a point cloud started with a registration of the individual high-density point clouds generated from multiple scans created in the previous phase. All scans’ positions were imported in Autodesk Recap^27^ for registration using the Auto-Registration feature However, it was required for few to specify common points since the Auto-Registration feature failed to register these scans. The registered raw high-density point cloud is shown in Fig. 7c.

The third phase (3D modeling and properties extraction) involved modeling the main body skeleton, creating families, and adding structural parameters to all families and details. The modeling main body was made based on the generated 3D meshed model for the three parts of the obelisk. The point clouds are meshed and then textured using the images taken during the scanning phase (Fig. 7d). The 3D meshed model was then imported to Autodesk Revit^28^. Dynamo^29^ (Open-source graphical programming for design) was used to calculate the properties of the three parts such as volumes, cross-section areas at the different sections, and center of gravity as depicted in Fig. 7e. The 3D meshed model and calculated properties were used in structural analysis and finite element modeling.

### Non-destructive assessment and laboratory testing

A comprehensive non-destructive investigation and laboratory testing were carried out to assess the obelisk structure and the granite properties before erection and before the anchoring system design as an essential requirement. The non-destructive techniques used are rebound number measurement by rebound hammer, infrared-thermography, and Ground Penetrating Radar (GPR). The performed laboratory tests were physical, mechanical, chemical, mineralogical, and petrographic. The obelisk body was surveyed by a total station and scanned by a 3D laser scanner to geometrically link the non-destructive measurements with the obelisk faces. A detailed description of the techniques and the results are presented in the methods section.

**Figure 8:**
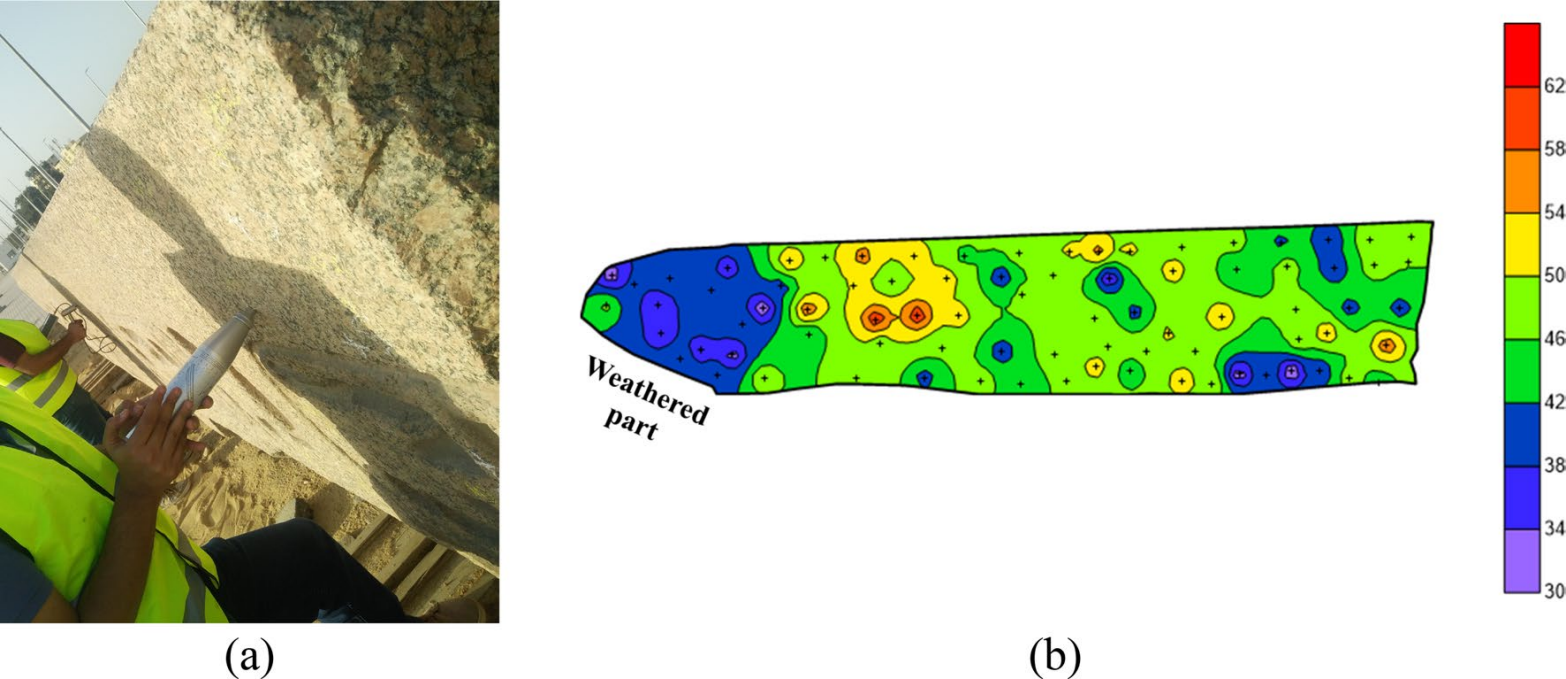
The rebound hammer testing. (**a**) Field work of rebound hammer testing. (**b**) the result represented as a contour map.

The rebound hammer measurements covered the obelisk faces to indicate non-destructively the material hardness and could estimate the strength variation in the obelisk faces (Fig. 8). A grid on each obelisk face was created to define the points of the rebound hammer measurements (ASTM D5873 – 14)^30^.

**Figure 9:**
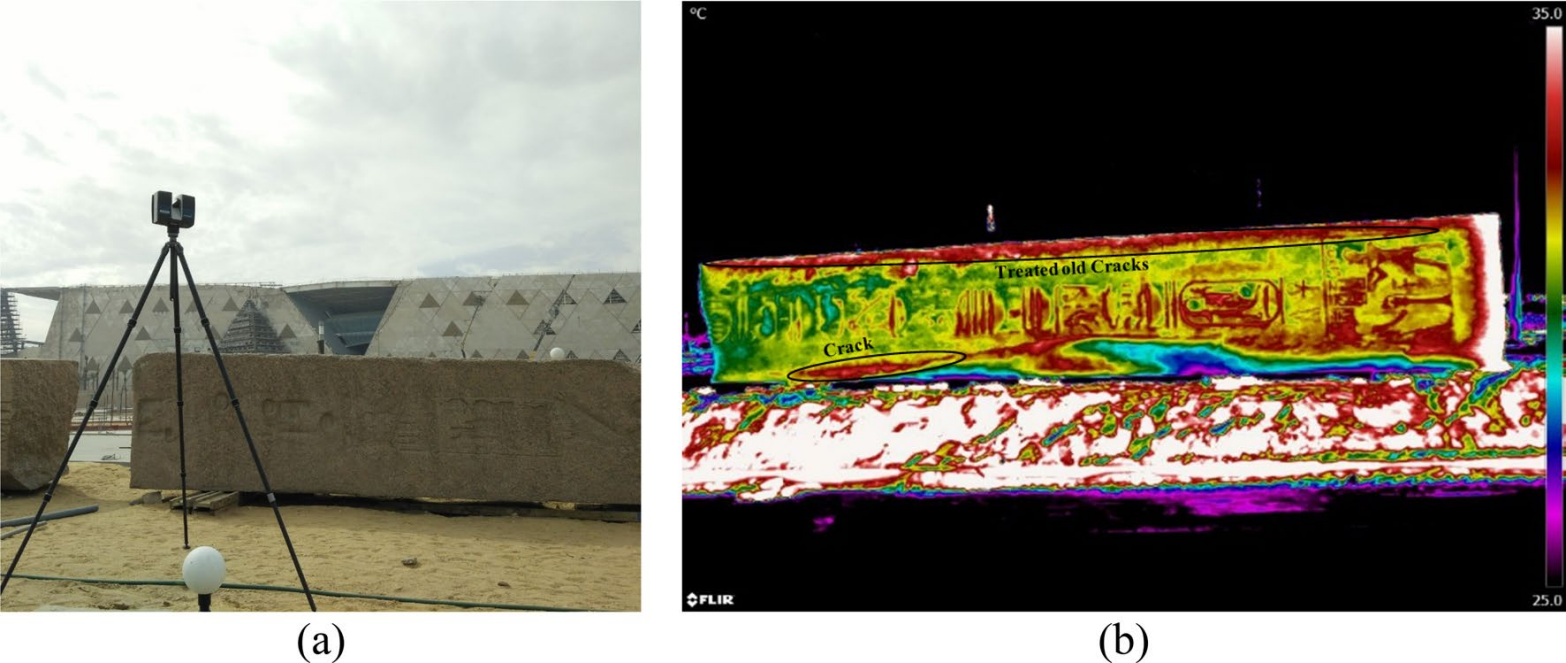
The Thermal imaging investigation. (**a**) the field work of thermal imaging. (**b**) an example of the produced thermal images.

Thermal imaging investigation for each face was carried out allowing detection of superficial cracks, moisture, weathered zones, or material heterogeneity that cause variation in material strength (Fig. 9). All the thermography scanning operations of the obelisk were executed using the passive thermography approach. Each face of the obelisk was scanned separately for 24 h.

**Figure 10:**
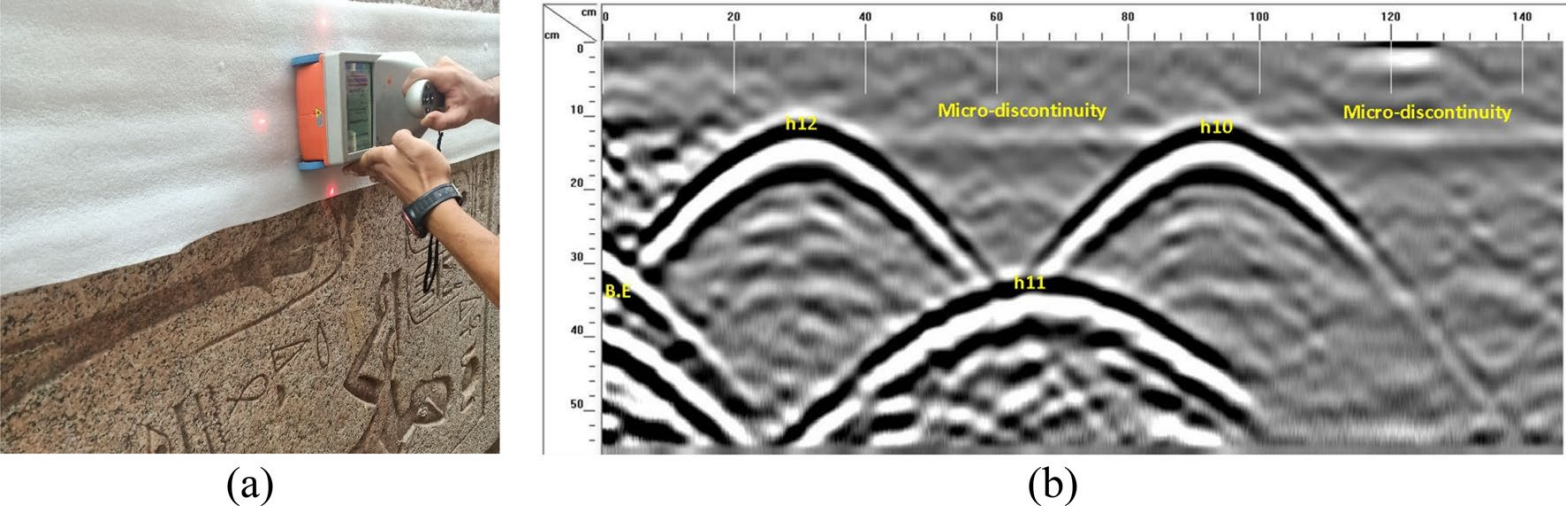
GPR measurements on the obelisk. (**a**) Field work of GPR. (**b**) an example of the GPR results.

GPR survey was carried out because it is a non-destructive fast technique and due to its ability to detect the internal discontinuities and anomalies of structures^31,32^ (Fig. 10). GPR survey was done in two rounds: the first round covered the two broken parts of the obelisk searching for hidden discontinuities using 900 MHz and 1500 MHz antennas whilst the second round, using a 1600 MHz frequency antenna, focused on the face where holes were drilled to connect the two parts by steel anchors aiming to detect any possible micro-discontinuities caused by drilling.

**Figure 11:**
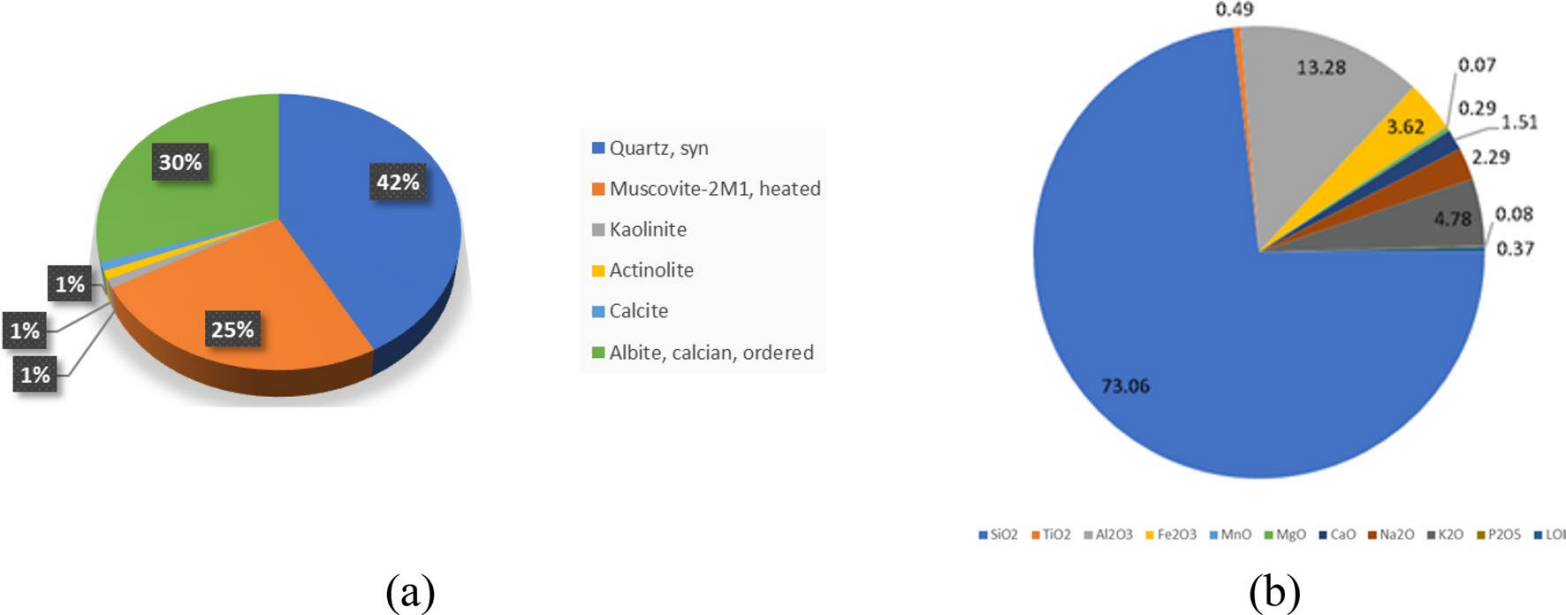
Results of XRD and XRF tests on a fallen sample from the obelisk. (**a**) the mineral composition represented in 3D pie chart. (**b**) the chemical composition represented in a pie chart.

X-ray diffraction (XRD) and X-ray fluorescence spectrometry (XRF) laboratory testing were carried out on fallen small material from the obelisk to determine the mineral and chemical composition of the obelisk (Fig. 11). XRD is considered the most promising technique used for the identification of minerals in a rock sample, while XRF is the most used technique to detect and quantify the chemical composition of rocks and minerals in a sample.

**Figure 12:**
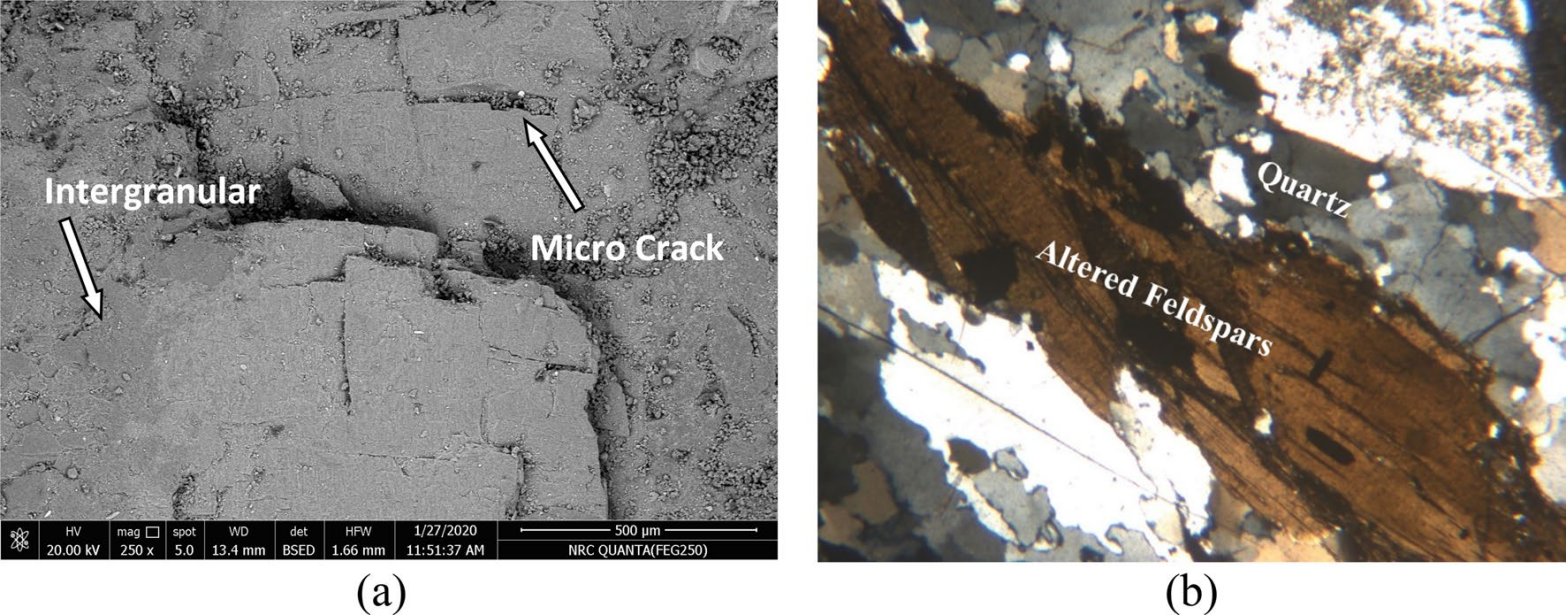
SEM and petrography of a fallen sample from the obelisk. (**a**) interpretation on SEM photo. (**b**) interpretation on petrography photo.

Petrographic study (using a polarizing microscope) and Scanning Electron Microscope (SEM) testing were carried out on the same samples to image precisely the internal structure of the material, allowing for the investigation of the surface texture, chemical composition, and crystalline structure of the sample (Fig. 12).

**Figure 13:**
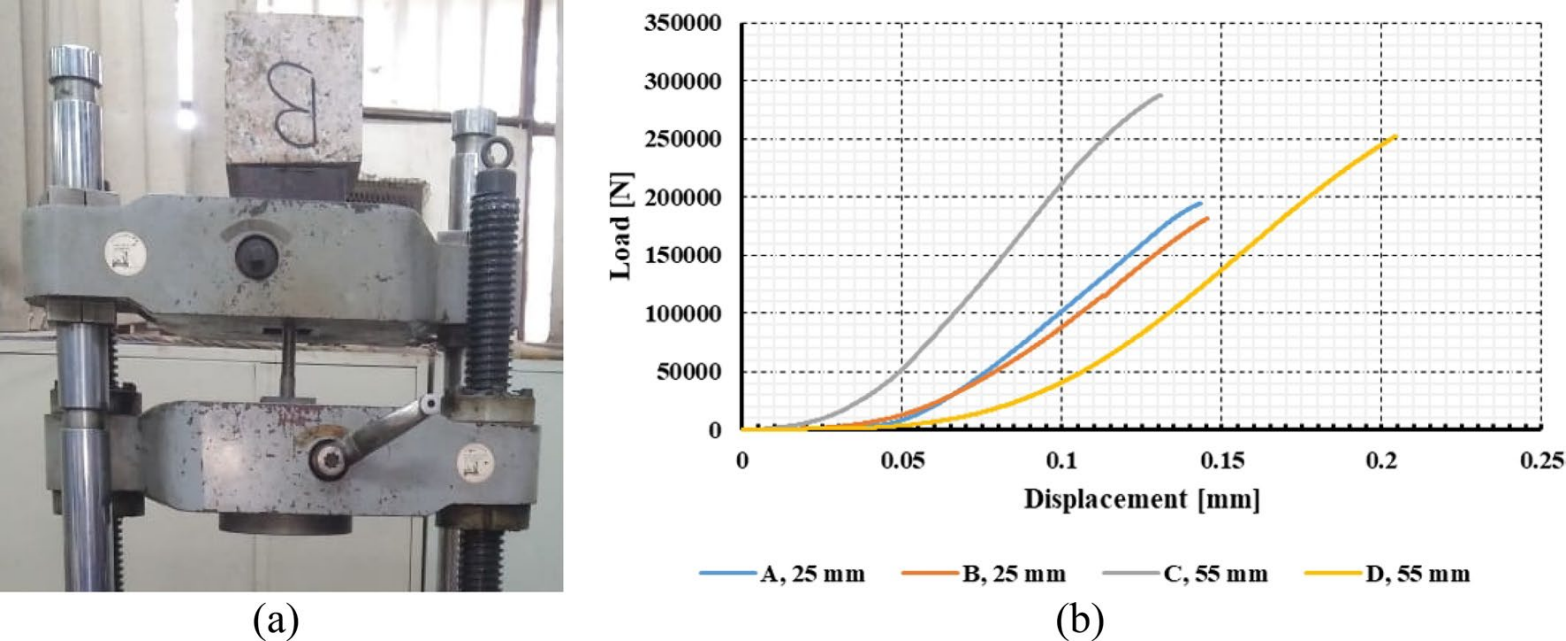
Pull out laboratory testing. (**a**) pull-out testing of an anchor. (**b**) displacement load representation of the anchors.

**Figure 14:**
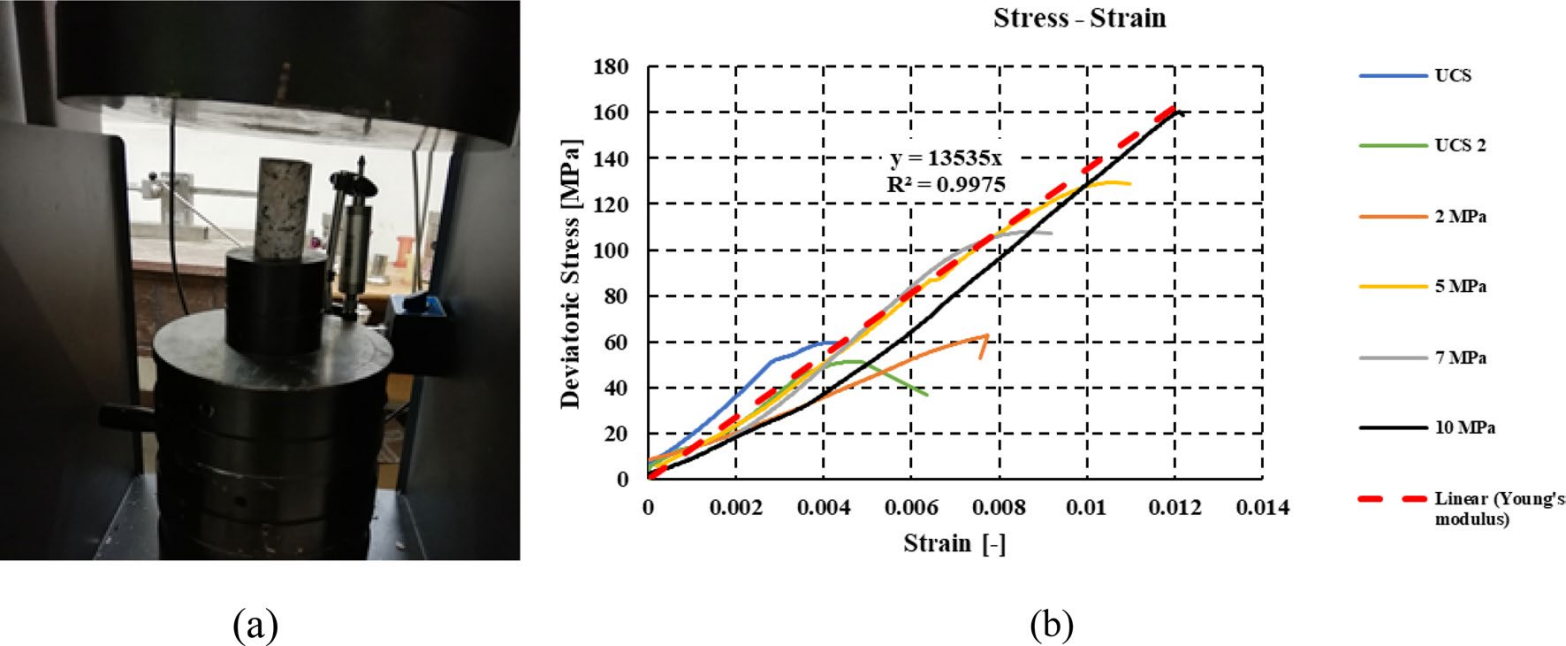
Granite laboratory testing. (**a**) granite sample under compression machine. (**b**) stress–strain curve of uniaxial and triaxial testing with average modulus of elasticity.

The connection between the different pieces of the obelisk was designed using stainless steel anchors. The embedment procedure was performed in fresh granite, avoiding weathered granite, in order to ensure proper force transfer. Therefore, a series of anchor pull-out laboratory tests were conducted as shown in Fig. 13. The tests were performed on four blocks obtained from a quarry of a similar rock type of the obelisk (red granite, Aswan). Prior to the pull-out tests, the mechanical and physical properties of the granite were quantified using a series of triaxial compression (single-stage, TC), unconfining compression (Uniaxial compression, UC), and tensile splitting (TS) tests (Fig. 14). It was found that the average Uniaxial Compressive Strength (UCS) value estimated from the rebound hammer for all faces of the obelisk was close to the laboratory results. Generally, the properties of the tested granite were in agreement with the obelisk granite.

The anchors used in the tests were from the same material used for the obelisk erection. The diameters used for testing were 25 mm, and 55 mm, while in the erection bolts the diameters were 55 mm and 70 mm. This was due to the limitation in dimensions of the available granite samples used for testing. The laboratory tests were carried out based on the procedure suggested by Benmokrane et al.^33^. The outputs of the pull-out tests are the bond strength of the entire anchorage system (the rock, the adhesive material, and the bolt) as well as the failure mode. The bond strength for the 25-mm diameter bolts was 15.95 MPa, while it was 10.42 MPa for the 55-mm diameter bolts. The failure occurred at the anchor/adhesion interface. The failure pattern of the granite blocks showed the maximum damage near the bottom of the hole.

### Structural modelling

**Figure 15:**
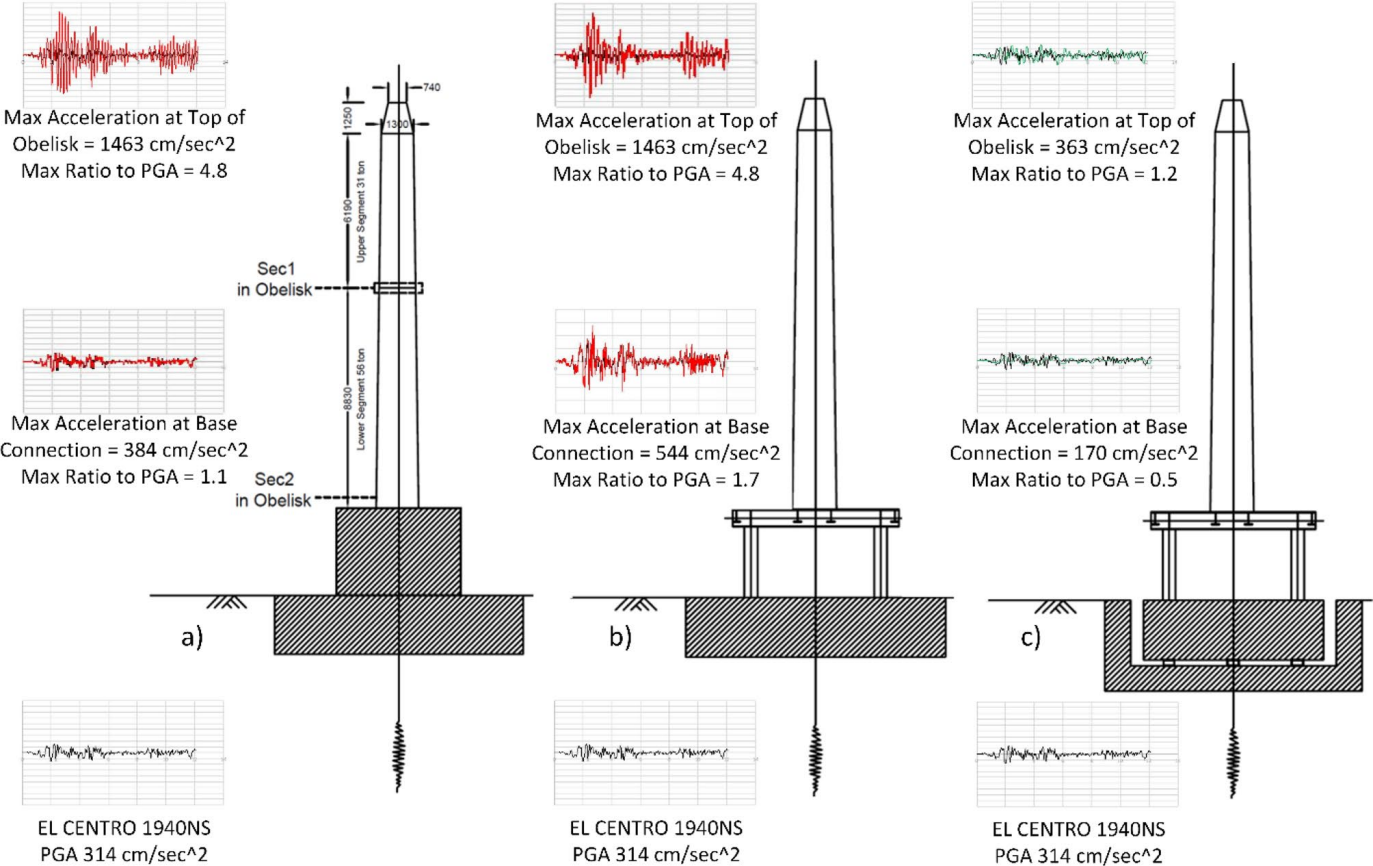
Geometric description of the different elements of the obelisk, and a comparison of the seismic response for three different base design cases, which are (**a**) Obelisk fixed on concrete base. (**b**) Obelisk fixed on steel frame supported on concrete base. (**c**) Obelisk fixed on steel frame supported on concrete base with base isolation.

After the restoration process, the obelisk was delivered into three main elements which were: (1) the original base, (2) the bottom segment of length about 8.83 m, and weighs about 56 tons, and (3) the top segment of the obelisk, in approximated length 7.44 m and weighs about 31 tons (Fig. 15). The structural team had two main objectives. The first objective was to design the elevated steel frame along with its foundation, which has a width of 4.5 m in both horizontal direction and a height of 3.7 m approximately. The second objective was to design the anchor connection between the two obelisk segments and the connection of the base with the steel frame. The major design challenge was the ability to resist the lateral loads, mainly seismic loads, on such slender tall structures. The fact that the obelisk was elevated to expose the Cartouche at its base increased the lateral stability challenge. To reduce the impact of the seismic loads, base isolation was adopted. Introducing base isolators is a well-established technique used to dissipate seismic energy as it decouples the building from the underneath shaking ground^34,35^. It is worth mentioning that in ancient times, simplified techniques similar to base isolations were occasionally used^36,37^.

Due to the importance of the obelisk, the design criteria were developed such that the stresses from the unfavorable load combination should remain below the yield strength with a considerable factor of safety. Therefore, the non-linear modes of failures were intentionally avoided, and the developed stresses are within the elastic range. SAP2000 model was used to determine the loads on the steel frame, and the straining actions at the different sections of the obelisk. The interaction between the obelisk and the supporting steel frame was analyzed using the numerical model developed in ANSYS due to its complexity. The connections on the other hand are designed using compatibility equations based on the straining actions calculated from the numerical models. The mechanical properties were modeled using linear elastic constitutive model due to the low stress level anticipated. The granite material was characterized based on laboratory test results providing an average modulus of elasticity of 13.5 GPa and compressive strength of 47 MPa and tensile strength of 4 MPa (Fig. 14). For the steel beams, the adopted linear elastic stress strain curve has a modulus of elasticity of 200 GPa and yield strength of 250 MPa. The stainless-steel material model adopted was linear elastic model for stainless-steel grade 304 with Young’s modulus of 193 GPa, yield strength of 205 MPa and tensile strength of 515 MPa.

The effectiveness of base isolation was evaluated through developing finite element models using SAP2000 software and comparing the results of three alternative base connection approaches (Fig. 15)^38^. In the first model, the obelisk was fixed on a concrete base without base isolations, which is close in its representation to the original obelisk and its granite footing. In the second model, the obelisk was elevated on a steel frame fixed on a concrete footing. The third model resembled the second one except for the introduction of base isolations beneath the concrete footing. In all models, the obelisk was subjected to El Centro earthquake time history record. As shown in (Fig. 15), the peak acceleration on top of the obelisk for the first and second models where no base isolations were used was about 4 times higher compared to the response induced by the base isolations. Therefore, the base isolation approach was adopted and situated beneath the concrete footing instead of the steel columns. This approach was effective because of the high overturning moment that triggered axial tension force on the bearings. It was advantageous to benefit from the heavyweight of the concrete footing by placing the isolators beneath it to reduce the tension forces. This design inspired the concept of a unique tunnel around the concrete footing to eliminate the lateral and hydrostatic forces from the soil to the concrete base. In addition, the tunnel facilitates regular maintenance for the base isolation. The final structural design yielded eight Lead Rubber Bearing (MLRB) provided by MAURER.

**Figure 16:**
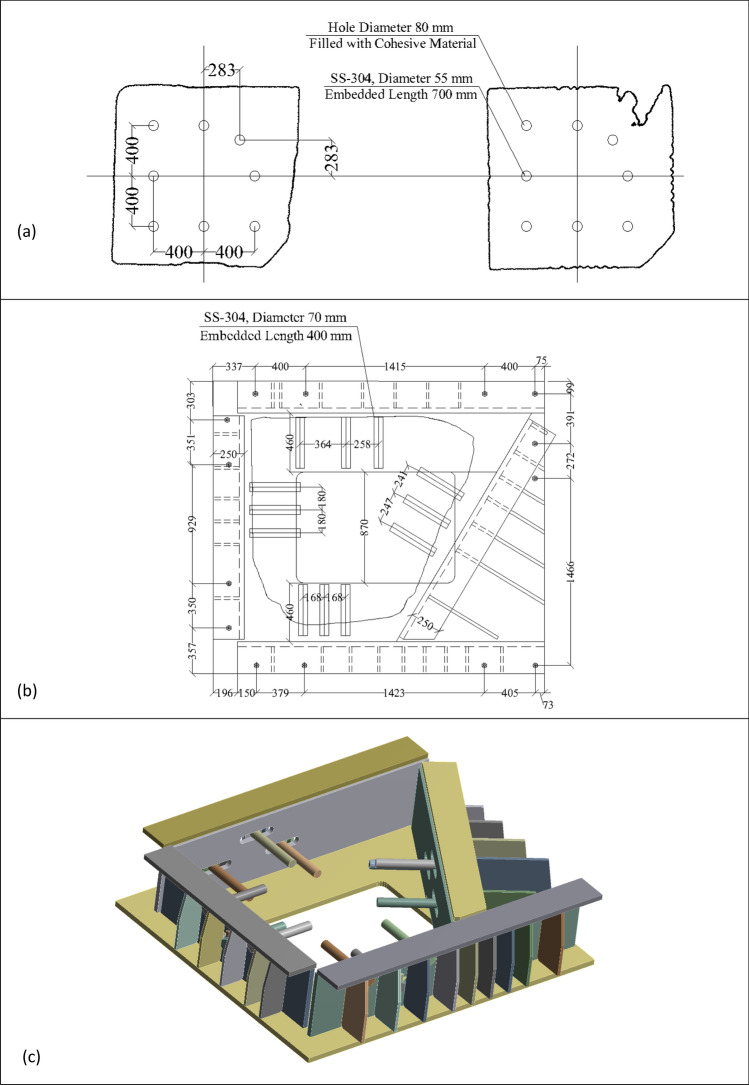
(**a**) The anchor connection of the two parts of the obelisk, (**b**) The anchor connection between the obelisk and the bottom steel frame, (**c**) the steel cage from the ANSYS model.

Stainless-steel anchors of grade SS-304 were used to connect the two segments of the obelisk and the bottom part to the elevated steel frame. The connections were designed according to the provisions of the Egyptian Code of Practice mainly to resist the obelisk's own weight and the seismic lateral loads (ECP 2012; ECP 2005)^39,40^. For the middle connection, eight vertical stainless-steel anchors of diameter 55 mm were installed (Fig. 16a). The critical seismic forces on this cross-section were directly obtained from the numerical model assuming rigid connection between both parts which yielded a bending moment of 400 kN.m and shear force of 90 kN and assuming strain compatibility between granite and stainless-steel anchors, a maximum combined normal and shear stress of 104 MPa is obtained which comprises around 50% of the yield strength and 20% of the tensile strength of the anchor.

Finally, to connect the obelisk base to the steel frame an intermediate steel cage was designed of a base plate with openings to expose the cartouche (Fig. 16b,c). Then, customized built-up sections were welded to the base plate to develop a system of beams covering the four sides of the obelisk. Anchors of diameter, 70 mm were installed in horizontal directions between the steel beams and the obelisk to avoid any interference with the cartouche face, in addition, a portion of the obelisk base rests on the steel cage by bearing. To check the design of this connection, critical seismic forces were obtained from the model to check both the granite material and anchors. The applied bending moment of 1500 kN.m along with the obelisk’s own weight yielded a maximum shear stress of 110 MPa which comprises around 54% of the yield strength and 21% of the tensile strength of the anchor.

**Figure 17:**
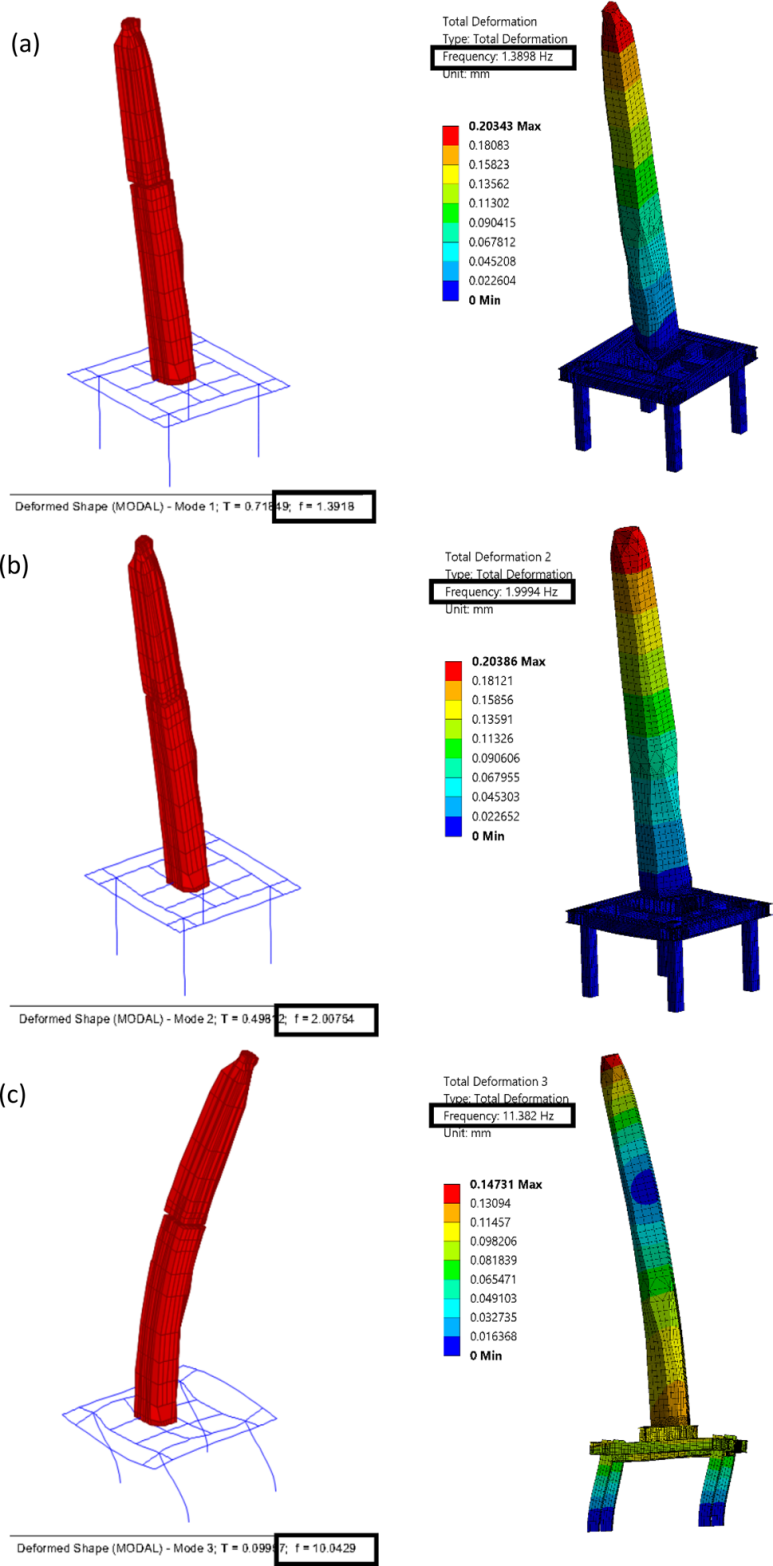
Comparison of natural frequencies between SAP2000 model on the left and ANSYS model on the right for, (**a**) Mode, (**b**) Mode 2, (**c**) Mode 3.

**Figure 18:**
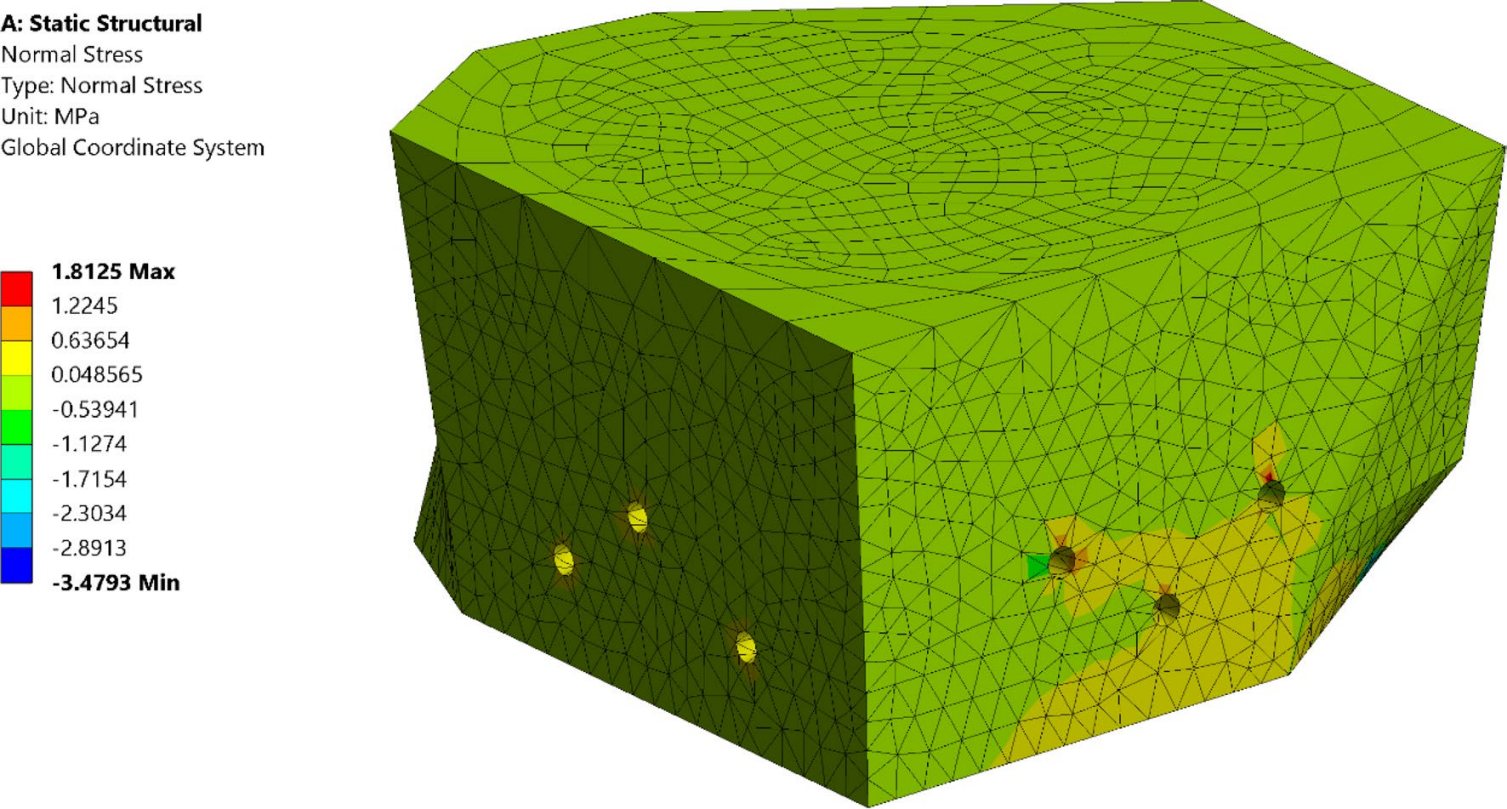
Tensile stresses in the obelisk due to thermal loads.

The connection between the obelisk granite material and the steel cage can result in additional thermal forces on the obelisk. In order to mitigate such forces, horizontal slots in the beams at the anchor's location are introduced and separation between the four steel elements was made to allow tolerance for differential temperature deformations between the two structures. In order to evaluate the effectiveness of those mitigation measures, a detailed 3D solid finite element model was developed using ANSYS software (ANSYS, INC)^41^. As a summary of the numerical model aspects, 3D solid elements were used for modeling of the obelisk, base connection and supporting steel frame using SOLID186 element in ANSYS which is a 20 node second order solid element. Bonded contact between steel members in the supporting steel frame was used. In modeling of the base connection, frictional contact between obelisk and steel base plate and between anchor washer and vertical plate is used along with bonded contact between anchor shank and obelisk body. For contact formulation, pure penalty formulation was used for bonded contacts while augmented Lagrange formulation was used for frictional contacts. This numerical model was compared with the simplified one developed in SAP2000 in terms of modal properties. The natural frequencies of both models for the first three modes show correlation as shown in (Fig. 17). The results of the thermal analysis showed that the mentioned measures were able to reduce the tensile stresses on the obelisk body to about 1.8 MPa compared to the maximum tensile strength of 4 MPa obtained from lab test results as shown in (Fig. 18).

### Surveying measurements procedure

Surveying measurements to ensure the verticality of the obelisk during erection was another challenge. The four facets of the obelisk are not truly 2D planes due to the deterioration of these surfaces. The four facets of the obelisk are not even vertical planes due to the inclination of its facets around the axis. Moreover, the four edges are not straight. Hence, it was impossible to find the true pair of lines in two vertical planes perpendicular to each other on two intersected facets. To overcome these difficulties a line passing through mid-points of the distance between every two parallel facets in different sections along the height of the lower part of the obelisk is defined on two intersected facets. The two vertical planes passing through these two lines should pass through the centerline of the obelisk. A mid-point each one meter along the height was located in both eastern and southern facets in which two lines were projected in the two intersected facets. Two station points were selected in front of the two facets to observe and measure the deviation from the verticality of the two lines of the lower part of the obelisk.

**Figure 19:**
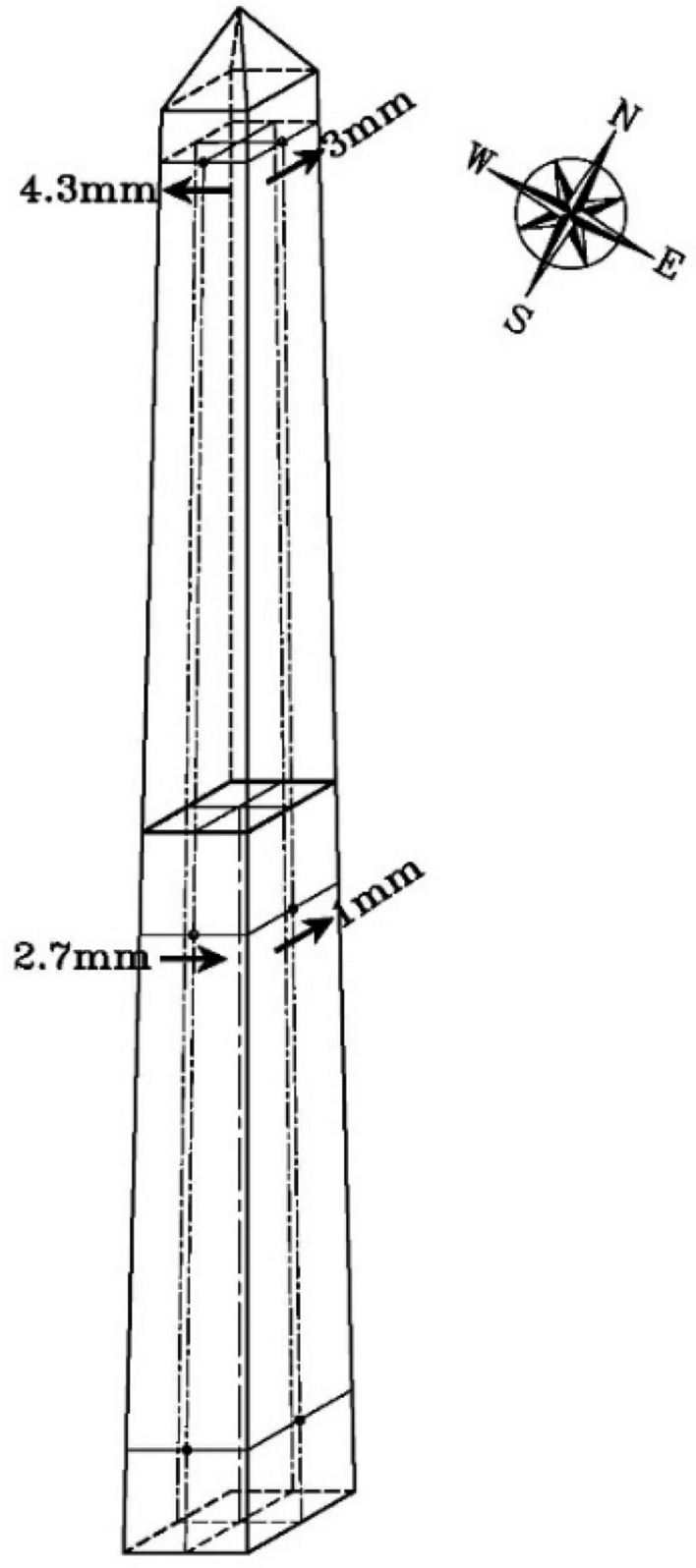
Actual inclination after erection.

Measurements were taken in two phases. The first phase measured the lower part and the second measurement was taken after the whole obelisk was installed at its permanent location. Measurements for the lower part of the obelisk (8.83 m) concluded no significant horizontal displacement between top and bottom of the lower part of the obelisk (only 1 mm horizontal displacement of the eastern facet, toward north direction, and 2.7 mm horizontal displacement of the southern facet toward east direction) as shown in Fig. 19 Measurements for the whole obelisk (16 m) concluded also no significant horizontal displacement between top and bottom of the whole obelisk (3 mm horizontal displacement of the eastern facet, toward the north direction, and 4.3 mm horizontal displacement of the southern facet toward west direction) as shown in Fig. 19.

**Figure 20:**
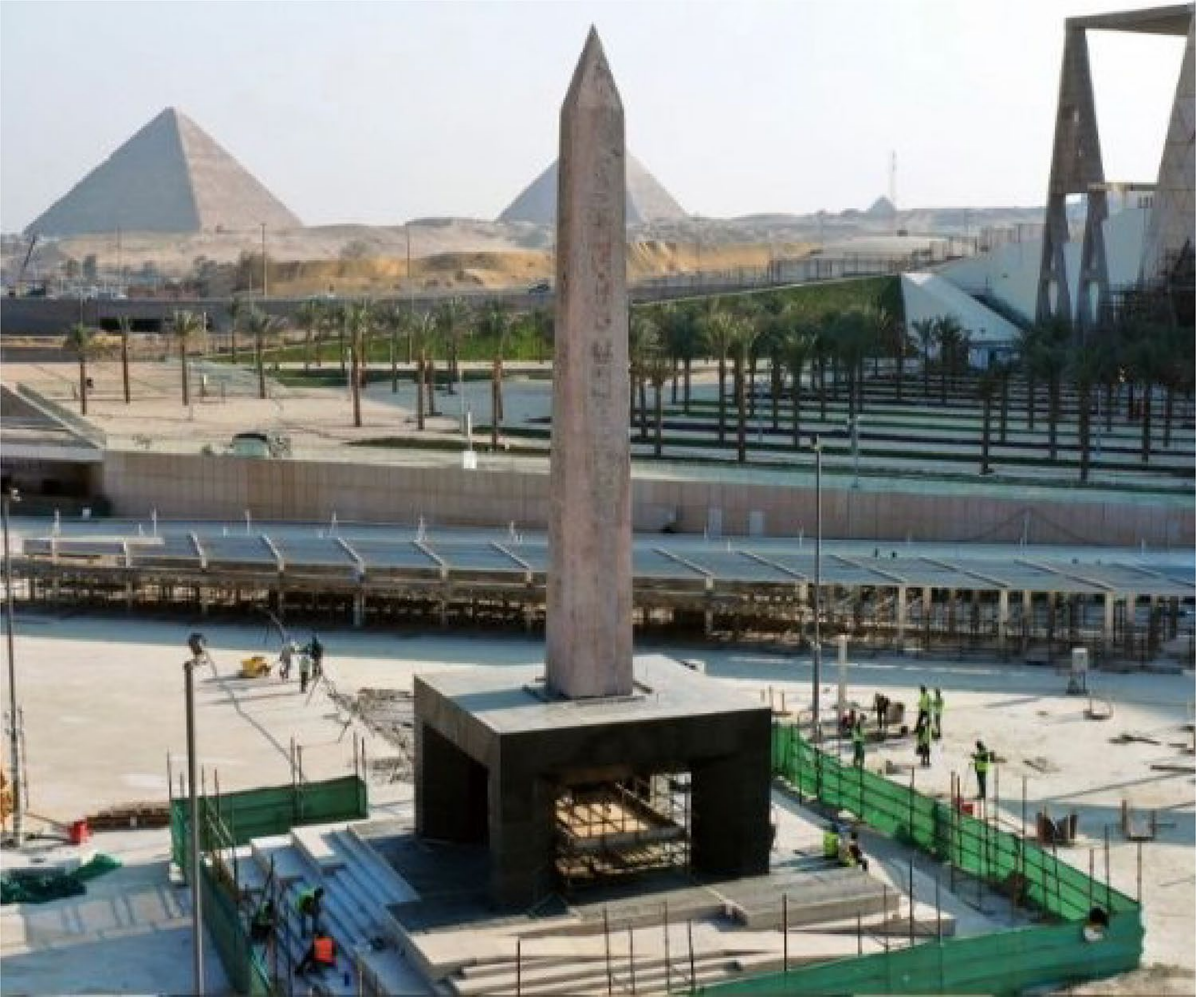
Obelisk after erection on its elevated platform.

As shown in Fig. 20, the obelisk is now erected at the entrance piazza of the Grand Egyptian Museum, which is currently under construction and scheduled to be opened in 2022.

## Conclusion

The paper summarizes the process of assessing the conditions of a damaged obelisk, then designing and erecting it to be the first elevated obelisk. A truly multidisciplinary problem was tackled by different experts from the concept stage to the final implementation. The effort was coordinated to reach the final product. The entire team was focused on creating a conservative and sustainable solution for the challenges associated with the handling and exhibition of such delicate object representing our world’s cultural heritage. This combined effort put together not only the knowledge of several scientific expertise but established also bridges between destructive, non-destructive, analytical, and numerical modeling approaches that would be useful for similar projects. The process started by performing 3D laser scanning and modelling the obelisk current condition. Subsequently, field and laboratory material assessment were carried out to decide on the best anchoring approach. Structural modeling was performed to design the elevated frame, the associated base isolations, and to design the anchor connection between the elevated frame and the obelisk. During erection, survey work was performed to ensure verticality of the obelisk.

## Supplementary Information

Supplementary Information.

## Data Availability

The datasets generated during and/or analysed during the current study are available from the corresponding author on reasonable request.
