# Supplementary material for: The restoration and erection of the world’s first elevated obelisk
Source: Sci Rep. 2023 Feb 4;13:2065. doi: 10.1038/s41598-023-29092-z (PMC9899242; doi:10.1038/s41598-023-29092-z)
Supplement: Supplementary file 1 — Supplementary Information. [file 41598_2023_29092_MOESM1_ESM.docx]

**METHODS**

**Non-destructive Assessment and Laboratory Testing**

**Non-destructive assessment. *Total station surveying*.** A detailed topographical mapping for the obelisk was necessary before conducting the non-destructive tests. This surveying was done using Sokkia IX 1005 total station. The obelisk was divided into two main parts. Part I had four visible faces, while part II had five visible faces. We coded the faces as follows: A and C for both-sided faces, B for the top face, D for the plane of separation, and E for the face representing the base of the obelisk. A sketch for each face for the two parts is shown in Extended Data Figure 1.

***Schmidt hammer.*** The Schmidt hammer rebound number indicates the material hardness and could be used to estimate several rock properties, particularly Uniaxial Compressive Strength (UCS)^36,37,38,39,40,41^. Furthermore, it provides a good indication of the weathering state of rock^42,43,44,45^. The limits of the taken measurements were set according to Sjöberg^45^: low rebound number (< 30) means a soft and weathered surface, and high number (>55) means a hard, un-weathered surface. A grid was defined on each obelisk face to locate the points of the Schmidt hammer test (Extended Data Figure 2). The following was considered in choosing these points:

- The surface should be as flat as possible.
- The distance between the grid points should not be less than 150 mm (when possible)
- The number of points on each face should be sufficient enough to account for the natural heterogeneity of the obelisk material.
- The archaeological value of the obelisk when performing the test at each point, i.e., cracked and heavily weathered areas as well as the hieroglyphic inscriptions were avoided.

Surfer® from Golden Software was used to plot the rebound number at each point location. Then, contours were used to represent such values to locate the weak zones within each face. The rebound number contour maps have indicated several weak zones on different obelisk faces. It can be interpreted that the main causes of these weak zones are weathering effects, previously mechanical stresses induced damages or both. Furthermore, relatively strong zones were observed at the locations (or near) of mafic lenses/ xenoliths (especially at faces C1 and E2). It could indicate significant material deterioration at the obelisk separation planes (faces D1 and D2). Furthermore, a descriptive statistical analysis had been carried out on the UCS values estimated from the Schmidt hammer test (Extended Data Table 1). Extended Data Figure 3 shows the mean UCS values for each obelisk faces, and it shows that mean UCS are close for faces A1, A2, B2, C1, C2, and E2. Extended Data Figure 4 shows the minimum and maximum UCS values for all faces, while face B1 shows a lower mean UCS compared to the before-mentioned faces. On the other hand, faces D1 and D2 (i.e., break planes of the obelisk) show the lowest mean UCS values compared to other faces. This indicates weaker zones, which may be caused by weathering or due to the previously induced mechanical stresses on these faces. The maximum UCS value for faces A1, A2, B1, B2, C1, and C2 were close and were significantly lower for faces D1 and D2. This is expected as face D1 and D2 are separation planes (i.e., planes where the obelisk has been broken). This agreed with UCS mean values and rebound number contour map of these faces, The minimum UCS values observed for faces A1, B2, C1, and E2 are close to each other. Meanwhile, faces A2, B1, C2 and D2 showed close minimum UCS values but these values are lower compared to the before-mentioned faces. Face D2 showed eventually the lowest value of all faces. When considering both minimum and maximum UCS values for each face, it could be indicated that faces A1, B2, and C1 are more competent (i.e. highest minimum and maximum values). However, faces D1 and D2 show significant material deterioration (i.e. lowest minimum and maximum values).

***Infrared Thermography.*** Thermographic measurements were performed using a thermal imaging camera (FLIR T540) with a measurement frequency of 1 hour to remotely detect the material anomalies (i.e., surface cracks or restoration material), and to diagnose moisture penetration or material heterogeneity that causes a variation in material strength. For ancient granite structures, thermography was used to detect material anomalies and inform repair methods ^46^. The thermal inspection was applied at different faces of the obelisk during the period of 10 to 18 December 2019 (Extended Data Figure 5). The thermography analyses were made passively, without relying on a thermal excitation source, using a camera resolution of 464 × 348 pixels. Considering that each imaged side of the obelisk has different dimensions/area, the thermal camera’s focal angle was chosen to ensure that the whole side is captured in one image (14°, 24°, and 42°). To ensure maximum contrast – i.e., choose adequate min/max scale – for each image, the image temperature scale was fixed between the following intervals 08:00 to 12:00; 12:00 to 15:00; 15:00 to 17:00; 17:00 to 19:00; 19:00 to 24:00; 24:00 to 06:00; and 06:00 to 08:00. The obelisk’s red granite material emissivity was assumed to be 0.80. The images were processed via the FLIR Studio software to generate logarithmic temperature scales to amplify the temperature contrast between the different sections of each face investigated (Extended Data Figure 6). The temporal sequences of the thermal images were laid over the skin of constructed 3D model comprising each of the scanned surfaces of the obelisk. The infrared thermography survey results (Extended Data Figure 6) have indicated several cracks and weak zones. The results matched with the later visual inspection from the site that major cracks existed in many places such as faces I-C, II-C, I-A, II-A, and I-E. Furthermore, it showed many places with different thermal properties (anomalies) which may be explained as mafic lenses/xenoliths and heterogeneity zones (different materials) in both parts such as faces I-C, and II-E. Additionally, infrared thermography inspection allowed us to determine the zones that were previously restored such as II-C. To illustrate the difference between the crack spots and the rest of the surrounding rock, face I-A (Extended Data Figure 6b) was chosen as an example. Extended Data Figure 7 shows the difference between two points: one on the crack (P) and the other one on the surrounding (S), in the rate of heat gaining and releasing (difference in thermal properties).

***Ground Penetration Radar (GPR).*** GPR has been used because of its proven ability to detect the internal discontinuities and anomalies of the structures^47^. Regarding data acquisition and processing the GPR instrument used in this study consisted of a Subsurface Interface Radar (SIR) System-4000, equipped with 900 and 1500 MHz and 1600 MHz shielded antennas (all produced by Geophysical Survey Systems GSSI). The GPR survey was conducted in two rounds. In the first round, the 900 MHz antenna was selected to cover the two parts of the obelisk body from different directions with reasonable resolution (Extended Data Figure 8). The 1500 MHz antenna was used in a few spots where a higher resolution was required. In the second round, 1600 MHz antenna was used to investigate the effect of anchor holes drilling at the base of the obelisk for erection, providing high resolution up to the expected penetration depth of 500 mm in granite. During the first round, two orthogonal faces in part I (I-A and I-B) were surveyed using the 900 MHz antenna in parallel lines as illustrated in Extended Data Figure 9a and b, respectively. Two outcropping cracks (Crack 1 and Crack 2) in the top of part I (Face I-B) were surveyed by the 1500 MHz antenna to investigate the depth of the cracks. In part II, two orthogonal faces II-B and II-C were surveyed using the 900 MHz antenna in parallel lines as illustrated in Extended Data Figure 9c and d, respectively. An outcropping crack (Crack 3) in Face II-A was investigated by the 1500 MHz antenna (Extended Data Figure 9e). Each survey line was named with a code formed of two parts: the antenna frequency and the survey line number. For instance, OB900-07 refers to line no. 7 surveyed by the 900 MHz antenna. The propagation velocity and dielectric constant in part I and part II were estimated from the 6 radargrams of Face I-A and the 5 radargrams of face II-C using the “depth to a known reflector” method^25,26^. The propagation velocity average of part I is 128.39 mm/ns and for part II is 131.40 mm/ns then the average dielectric constant (ε) of the medium was calculated to be 5.45 and 5.20, respectively. The results were compatible with the known range of dielectric constant in granite^48,49^: from 4.0 to 7.0. The GPR data were processed using the Radan 7 software package.

In the second round, the GPR survey was conducted mainly at the base of the obelisk using the 1600 MHz antenna (Extended Data Figure 10). There were three holes in each side of the obelisk base for the anchoring system (Extended Data Figure 10 b and c). The propagation velocity was estimated after time zero correction based on the known depth of the holes. The average dielectric constant was set to 4.03. The display gain used in the radargrams as represented using a color scale was 6 and using a greyscale was 12. The horizontal axis (X) and vertical axis (Y) are represented in metric units (first round) and cm (second round). The interpretation of anomalies is depicted on the radargrams (Extended Data Figure 11,12, and 13). The 900 MHz GPR results showed that the internal body in Part I and Part II of the obelisk is characterized by a relatively higher amplitude reflection. This could be interpreted as a change in physical properties such as being moist or having different concentrations of other rock-forming minerals. In Part I, several xenoliths were detected by the 900 MHZ from orthogonal directions (Face I-A and Face I-B) identifying their 3D shape and location. No xenolith was detected in Part II. In part 1, Crack 1 and Crack 2 were investigated by the 1500 MHz antenna that identified the internal extension of the two cracks. In Part II, the broken edge of the obelisk base, the extension of Crack 3 and Crack 4 were detected and geometrically identified by the 900 MHz antenna from two orthogonal directions (Face II-B and Face II-C). The extension of Crack 3 was investigated as well by the 1500 MHz from Face II-A identifying its depth of extension from this direction. In the obelisk base where boreholes were drilled, the GPR could detect two networks of micro-discontinuities at depths of about 15 cm and 35 cm (Extended Data Figure 14). The network of micro-discontinuities at 15 cm depth is clearer and more continuous in the radargrams than the network of micro-discontinuities at 35 cm depth.

**Laboratory testing. *Chemical, Mineralogical, and Petrographic Analysis*.**  Samples from small pieces have been collected (fallen on the ground) from the obelisk site under the supervision of the archaeological team at the Grand Egyptian Museum. It was required to identify the rock-forming minerals of the obelisk, especially for minerals of small sizes (clay size); therefore, X-ray diffraction (XRD) and X-ray fluorescence spectrometry (XRF) were applied on samples^50^ (Extended Data Table 2 and 3, respectively). For the petrographical study, thin sections were prepared for the petrographic polarizing microscope investigation (Extended Data Figure 15). Out of the study, the rock was identified as asperthitic granite, which is plutonic acidic igneous rock. Rock texture was fine to coarse-grained inequigranular, hypidiomorphic, perthitic, and piokilitic texture. The rock mineral composition was composed mainly of potash feldspar (microcline & orthoclase perthite), quartz, plagioclase, and biotite together with accessory opaque minerals and titanite. Secondary minerals were represented by sericite, iron oxides, clay minerals, and chlorite. For the scanning electron microscopy analysis, the samples were examined by the FES/SEM type Quanta FEG250 equipment with an EDAX-Unit to (i) determine elements that the obelisk consists of, and (ii) describe the corrosion surface. The first sample was found to have micro-cracks shown in (Extended Data Figure 16a). These cracks were spread all over the surface of the sample. This is considered as a result of the weathering conditions of temperature and pressure changes. Intergranular corrosion was also observed. The surface of the second sample (Extended Data Figure 16b) was found to be laminated. Some other areas within the sample were found to be porous. The pores contained no type of bacteria. In addition, it was observed that the sample contains potassium oxide (Na_2_O) and sodium oxide (K_2_O). This can be caused by the existence of clay content.

***Physico-Mechanical laboratory testing.*** As it was planned to erect the obelisk via rock anchors, it was necessary to test such an anchorage system. In general, the rock anchor system is composed of three parts; the anchor (made of stainless steel), the rock material (granite), and the adhesive material (epoxy or grout). To quantify the design parameters of erecting the obelisk using rock anchors, a series of rock bolt anchor pull-out tests were conducted. The laboratory tests were carried out based on a scientific procedure^27^. There are three possibilities of failure; anchor failure, rock failure, or adhesive material failure^51^. To justify the failure basis, the characteristics of the granitic rock material must be identified to expect the rock splitting. Granitic rock samples were selected to be similar to the granitic rock of the obelisk. In this respect, a series of triaxial tests were performed as well as tension and uniaxial compression tests according to ASTM (D3967-16)^52^ and ASTM (D7012-14e1)^53^. The determination of the compression and the tensile strengths are required to plot the full failure envelope of the granitic rock. The granite rock was assumed to follow the Mohr-Coulomb failure criteria with tension cutoff (Extended Data Figure 17). The pull-out test was conducted on four blocks of the same granite material to test the anchorage of the 25 mm and 55 mm anchors (Extended Data Table 4 and Extended Data Figure 18). The steel threaded bars were high-grade stainless steel. The fixation of these bars into the granite blocks was done using high-strength pourable epoxy grout which is a chemical anchor system qualified for anchor and post-installed rebar connections. The test monitored the applied load as well as the recorded displacement till failure (Extended Data Figure 19). Most codes and standards do not provide guidance in choosing the hole diameter as a function of the bolt diameter and rock type. In this case, the nominal bit diameter required for a 25 mm diameter threaded bar anchoring into granite was chosen to be 54.7 mm. While the nominal bit diameter for a 55 mm diameter threaded bar was about 75 mm (Extended Data Table 4). The embedment length used for anchorage testing of both 25 mm and 55 mm threaded bars was 150 mm. It must be noted that the embedment length should be at least five times the diameter. The tests (4 samples) were performed at standard room temperature as the blocks were rested on the top of the loading machine, while the steel bar was pulled out using grippers (Extended Data Figure 18). To determine the bond strength, the failure load is obtained from the pull-out test as well as the embedment length and the threaded bar diameter (Extended Data Figure 19). A constant bond strength along the embedment depth is assumed and is thus given by the following equation.


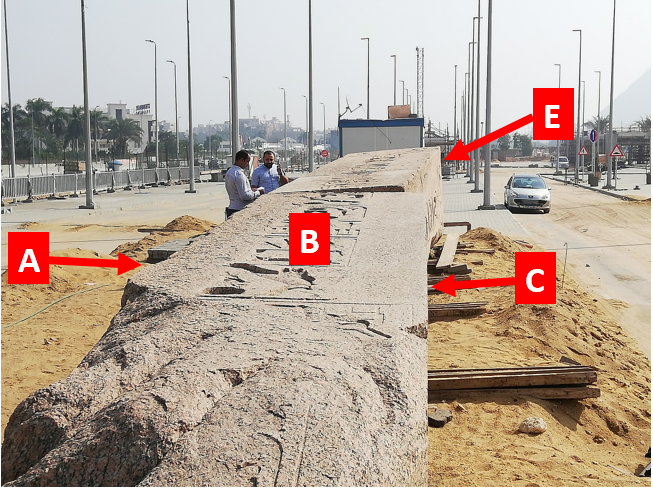


Fig. B-3: coding of obelisk faces

$N_{ult}=\pi hd\tau_{B}$ (1)

Where,

- *N_ult_* is the failure load in *N*.
- *h* is the embedment depth in *m*
- *d* is the rod diameter in *m*, and
- *τ_B_* is the bond strength in *Pa*.

It can be expected that the above-mentioned equation could obtain the bond strength for concrete with compressive strength of *f_c_* = 20 MPa very well and it is also valid for concretes up to class C50/60 (which could be applicable for the granite in our case)^54^. However, it requires the embedment depths 4.5 ≤ h /d ≤ 20 and the anchor diameters d ≤ 50 mm which are not always found in the test case as well as in the applications. The load-displacement curves are shown in Extended Data Figure 19. The test results are summarized in Extended Data Table 5. The failure occurred at the anchor/adhesion interface^27^. The failure pattern of the granite blocks shows the maximum damage near the bottom of the hole. The distribution of shear stress along a fully grouted rock bolt explains the failure pattern very well^55^. Assuming axisymmetric condition, the peak shear strength between the anchor and the grout is formed almost near to the base of the hole which is propagating in the granite block as tensile forces creating tension crack.

## Selection of Elastomeric Bearings

In order to reduce the seismic demand on the obelisk, elastomeric bearings were used at foundation of the obelisk. The selection of those bearings is applied through different stages. The first stage is to determine the location and arrangement of the bearings. Whereby, eight bearings were used and are symmetrically configurated to ensure uniform distribution of demand loads (Extended Data Figure 20a). Their location was selected to be between the foundation and the concrete base to take advantage of the concrete base weight to avoid rocking effect and tensions forces resulting from lateral loads. Moreover, this approach allowed for developing a maintenance tunnel around the concrete base, where the bearings are placed near the base edges for ease of accessibility. The second stage is to determine the initial characteristics of the elastomeric bearing and to develop a finite element model for the structural system. The natural period for any system depends typically on the mass (m) and the stiffness (k) as shown in equation 2. However, due to the presence of the elastomeric bearings, the stiffness is developed from two components (1) the bearing stiffness (K_b_), and (2) the frame stiffness K­_f_. Since the two components can be considered connected in series, the combined stiffness can be written as shown in equation 4. By substituting equations 2 and 3 a final natural period equation can be developed as shown in equation 4. Whereby, the bearing stiffness can be determined since the target period, frame stiffness, and the system mass are known.

$T_{1}=2\pi\sqrt{\frac{m}{k}}$ (2)

$k=\frac{K_{b}*K_{f}}{K_{b}+K_{f}}$ (3)

$T_{1}=2\pi\sqrt{\frac{m}{\frac{K_{b}*K_{f}}{K_{b}+K_{f}}}}$ (4)

Based on the preliminary analysis eight Lead Rubber Bearing (MLRB) provided by MAURER with equivalent linear stiffness of 1550 KN/m in both horizontal directions were utilized. In order to have a more accurate assessment of the selected bearings, a finite element model using SAP2000 analysis software was developed, where the obelisk and the steel frame were modeled using frame element, while the concrete base was modeled using shell elements (Extended Data Figure 20b) ^32^.

In the third stage, response spectrum analysis was done to check all parameters selected and to redesign all components. Then, seismic records and the selected bearing characteristics were applied to the finite element model to evaluate a suite of Engineering Demand Parameters (EDP’s). Several international standards such as the Eurocode EC8 and also Egyptian code allows to subject the structure to seven different earthquake records ^33,34,56^. Unfortunately, there is scarecty in the histroirc ground records in Egypt. Therefore, time history records from different locations are adopted in this study. Seven earthquake records were selected to cover short, medium, and long-period ground motions. In addition, those records are scaled to the same Peak Ground Acceleration PGA of 0.32g. Extended Data Figure 20c and d shows the selected ground motions for the original records and the scaled records, respectively. The selection of the ground records also takes into consideration the response spectrum as presented by the Egyptical Code of Practice (ECOP). Extended Data Figure 21 shows that the average of the seven records results in typically higher values compared to the ECOP response spectrum when they were all scaled to spectral acceleration of 0.32g at natural period equal to zero. In addition to that, Elcentro earthquake records is compared to the average of the seven ground motions, which showed higher peak acceleration at the top of the obelisk compared to the average of the seven records (Extended Data Table 6). Therefore, Elcentro was adopted to be used for further structural analysis of the obelisk and its supporting frame. Different EDPs were selected to show the response of the structure under the selected seven records and Elcentro earthquake record, which are the normal force in bearings, the shearing force in bearings, the max drift between top and bottom points, the stresses at base of obelisk, the stresses at base of steel column, and top point acceleration (Extended Data Figure 22). In addition, the average of the seven records and the square root of mean square is shown for comparison. It can be concluded that the selected bearing resulted in a suitable design in terms of lateral drift and no tension on the obelisk base.

**Structural Modeling**

Some challenges were encountered at the design stage of the stainless-steel anchor connections The main challenge can be attributed to the interaction between the elevated steel frame and the steel cage at the bottom connection. Whereby, local deformations and uneven load distributions were expected to occur due to the complex geometry of the base connection and the resulting load concentrations at some locations. For that reason, a simplified frame element model was not sufficient and a 3D finite element ANSYS model was developed (Extended Data Figure 23a). The model is developed from 20-node second-order structural solid elements The element is hexahedral in its most general shape. By combining some of the nodes, the element can degenerate into a triangle-based prism, quadrilateral-based pyramid, or tetrahedron. The model meshing resulting in 170,000 nodes with the aim of achieving a reasonable representation of the structural elements behavior. Base isolations were modeled using springs at the column's lower face, where its parameters are defined to represent the isolators vertical and lateral stiffness. The seismic loads were defined in a conservative manner by neglecting the seismic response modification factor (i.e., R=1). This factor is typically taken by a value greater than unity to reduce the seismic forces and allows for large deformations in the structure. However, due to the historic importance of the obelisk, it was decided not to take this approach and hence, large seismic loads were introduced to the structure.

The resulting tensile stresses on the obelisk base due to the gravity load in addition to the seismic lateral loads based on 475-year earthquake return period is 3.75 MPa (Extended Data Figure 23b). Meanwhile, test results showed an ultimate capacity of 4 MPa. The maximum stress on the top plate was 25 MPa, while the allowable stress is 210 MPa for the grade of the used steel (Extended Data Figure 23d). Finally, in the stainless-steel anchor bolts the maximum shear stress was 68 MPa, which comprises around 33% of the yield strength and 13% of the ultimate strength (Extended Data Figure 23d). The results showed the effectiveness of the 3D finite element model to present the concentrated stresses in the different elements. In addition, the model was able to represent the developed stresses and deformations due to temperature variations. In fact, the temperature impact was a significant parameter in the design process. This is because the coefficient of thermal expansion of the steel plates is not the same as the obelisk material. Therefore, the elevated steel frame and the attached steel cage with its anchors would expand or contract independently from the obelisk base. This behavior developed large stresses inside the obelisk base. In order to decouple the two materials, a horizontal slot is created in the steel elements to allow for some deformations between the steel and granite without inducing additional stresses. This approach was suitable for the problem since the main use of the anchors were to resist overturning moments through vertical movement.

One more challenge that needs to be mentioned is related to the construction process. Whereby, the selection of the adhesive material between the anchor and the obelisk granite material was governed by the hardening time of this material. This is because a large amount of adhesive material had to be injected in each anchor hole. The hardening time of the adhesive material has to be long enough to allow for adjusting the surveying and leveling of the obelisk while filling all the anchor holes. For that reason, high strength pourable epoxy resin groutadhesive material had been used with hardening time of 24 hours. The bond between the anchor and the obelisk granite material was tested in the laboratory and showed acceptable results as shown in the physico-mechanical laboratory testing section.

**References**

1. Armaghani, D., et. al. Prediction of the strength and elasticity modulus of granite through an expert artificial neural network. *Arabian Journal of Geosciences* **9**(1), 1–16 (2016).

https://doi.org/10.1007/s12517-015-2057-3

1. Breysse, D. ed. *Non-destructive Assessment of Concrete Structures: Reliability and Limits of Single and Combined Techniques: State-of-the-Art Report of the RILEM Technical Committee 207-INR* **1**. Springer Science & Business Media (2012).

https://doi.org/10.1007/978-94-007-2736-6

1. Aydin, A., & Basu, A. The Schmidt hammer in rock material characterization. *Engineering Geology* **81**(1), 1–14 (2005).

https://doi.org/10.1016/j.enggeo.2005.06.006

1. Kahraman, S. Evaluation of simple methods for assessing the uniaxial compressive strength of rock. *International Journal of Rock Mechanics and Mining Sciences* **38**(7), 981–994 (2001).

https://doi.org/10.1016/S1365-1609(01)00039-9

1. Kılıç, A., & Teymen, A. Determination of mechanical properties of rocks using simple methods. *Bulletin of Engineering Geology and the Environment* **67**(2), 237–244 (2008).

https://doi.org/10.1007/s10064-008-0128-3

1. Yagiz, S. Predicting uniaxial compressive strength, modulus of elasticity and index properties of rocks using the Schmidt hammer. *Bulletin of Engineering Geology and the Environment* **68**(1), 55–63 (2009).

https://doi.org/10.1007/s10064-008-0172-z

1. Betts, M. W., & Latta, M. A. Rock surface hardness as an indication of exposure age: An archaeological application of the Schmidt Hammer. *Archaeometry* **42**(1), 209–223 (2000).

https://doi.org/10.1111/j.1475-4754.2000.tb00877.x

1. Nesje, A., McCarroll, D., & Dahl, S. O. Degree of rock surface weathering as an indicator of ice‐sheet thickness along an east—west transect across southern Norway. *Journal of Quaternary Science* **9**(4), 337–347 (1994).

https://doi.org/10.1002/jqs.3390090404

1. Shakesby, R. A., Matthews, J. A., & Owen, G. The Schmidt hammer as a relative-age dating tool and its potential for calibrated-age dating in Holocene glaciated environments. *Quaternary Science Reviews* **25**(21–22), 2846–2867 (2006).

https://doi.org/10.1016/j.quascirev.2006.07.011

1. Sjöberg, R. Relative datings with the schmidt test-hammer of terraced house-foundations in Forsa Parish, Halsingland, Sweden. *Laborativ Arkeologi* **5**, 94–99 (1991).
2. Jo, Y.H. & Lee, C.H. Quantitative modeling and mapping of blistering zone of the Magoksa Temple stone pagoda (13th century, Republic of Korea) by graduated heating thermography. *Infrared Physics & Technology* **65**, 43-50 (2014).

https://doi.org/10.1016/j.infrared.2014.02.011

1. Annan, A. P. Ground Penetrating Radar Principles, Procedures, and Applications. *Sensors & Software* **278** (2003).
2. Daniels, D.J. *Surface Penetrating Radar*. The Institution of Electrical Engineers. London, UK (1996).
3. Davis, J. L. & Annan, A. P. Ground-penetrating radar for high resolution mapping of soil and rock stratigraphy. *Geophysical Prospecting* **37**(1), 531–551 (1989).

https://doi.org/10.1111/j.1365-2478.1989.tb02221.x

1. Lewis, D. W. & McConchie, D. *Analytical sedimentology*. Springer Science & Business Media (2012).
2. Mahrenholtz, C., Eligehausen, R. & Reinhardt, H. W. Design of post-installed reinforcing bars as end anchorage or as bonded anchor. *Engineering Structures*, **100**, 645-655 (2015).

https://doi.org/https://doi.org/10.1016/j.engstruct.2015.06.028

1. ASTM D3967-16. Standard Test Method for Splitting Tensile Strength of Intact Rock Core Specimens. *West Conshohocken, PA: ASTM International* (2016).

https://doi.org/10.1520/D3967-16

1. ASTM D7012-14e1. Standard Test Methods for Compressive Strength and Elastic Moduli of Intact Rock Core Specimens under Varying States of Stress and Temperatures. *West Conshohocken, PA: ASTM International* (2014).

https://doi.org/10.1520/D7012-14E01

1. BSI. Eurocode 2 : Design of Concrete Structures : British Standard. *British Standards Institution* (2008).
2. Li, C. & Stillborg, B. Analytical Models for Rock Bolts. *International Journal of Rock Mechanics and Mining Sciences* **36** (8): 1013–29 (1999).

https://doi.org/10.1016/S1365-1609(99)00064-7

1. Code, P. Eurocode 8: Design of structures for earthquake resistance-part 1: general rules, seismic actions and rules for buildings. *Brussels: European Committee for Standardization* (2005).


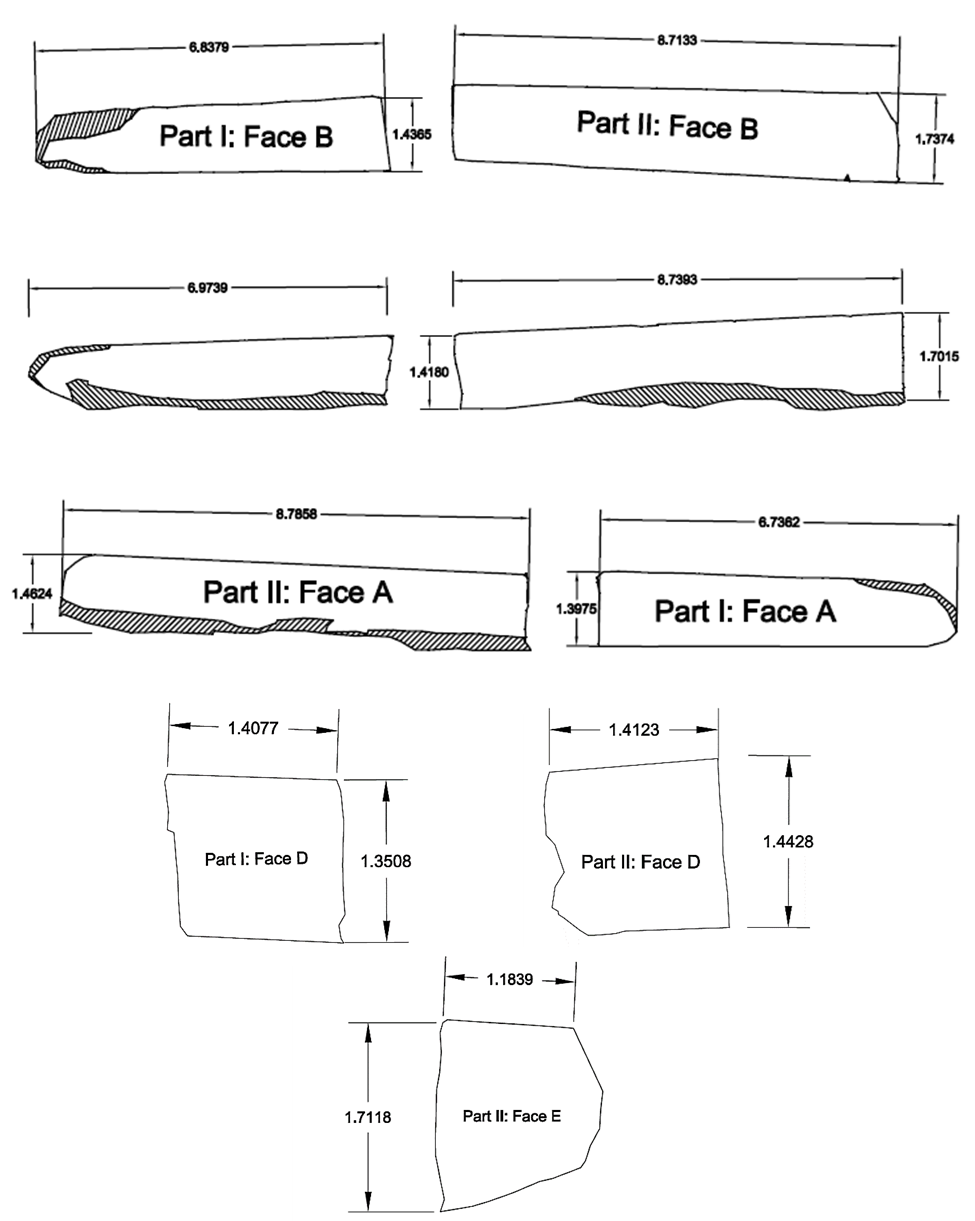

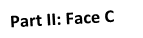

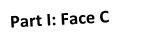


**Extended Data Figure 1 | A Sketch of the parts and faces of the Obelisk.**

Dimensions are metric.

| *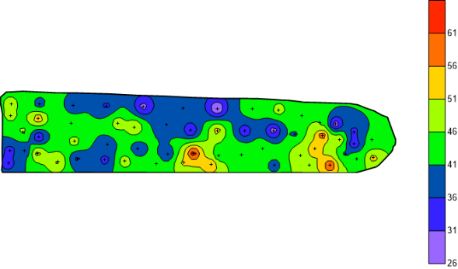* | *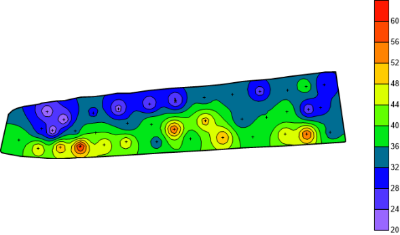* | *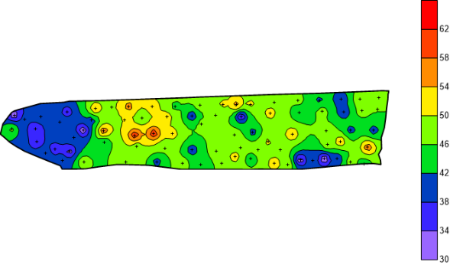* |
| --- | --- | --- |
| **Face A1** | **Face B1** | **Face C1** |
| *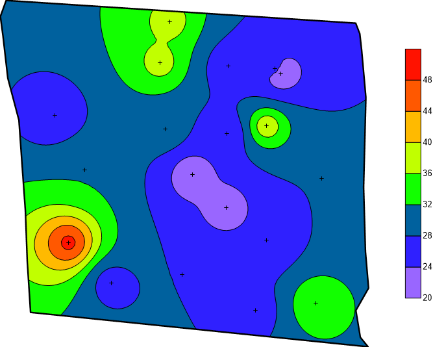* | **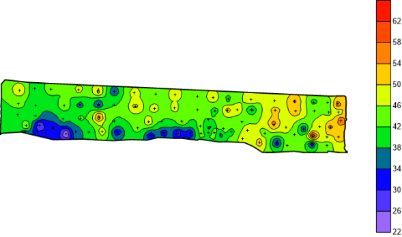** | *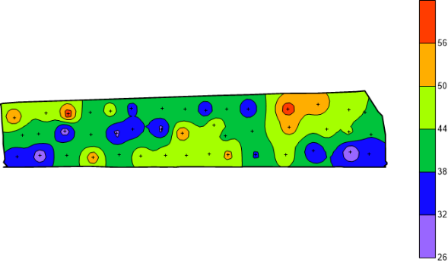* |
| **Face D1** | **Face A2** | **Face B2** |
| *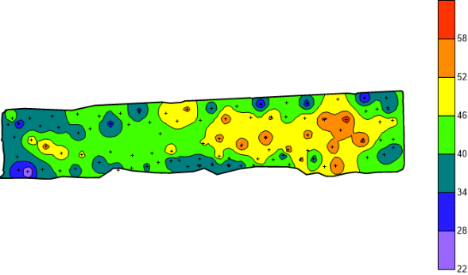* | *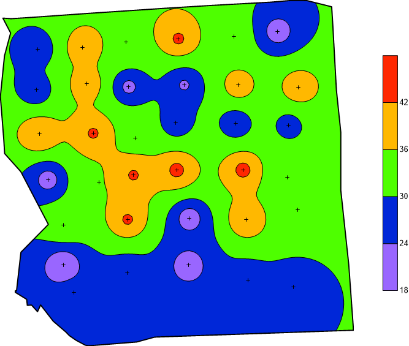* | *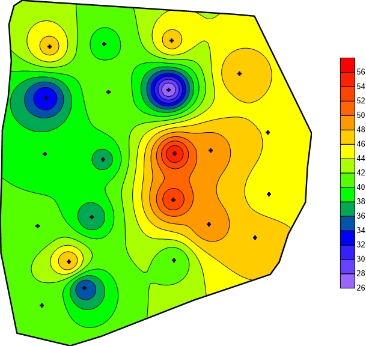* |
| **Face C2** | **Face D2** | **Face E2** |
| **Extended Data Figure 2 \| Rebound number contour map of the obelisk faces** | | |

Figure 3. Mean UCS values for each obelisk face


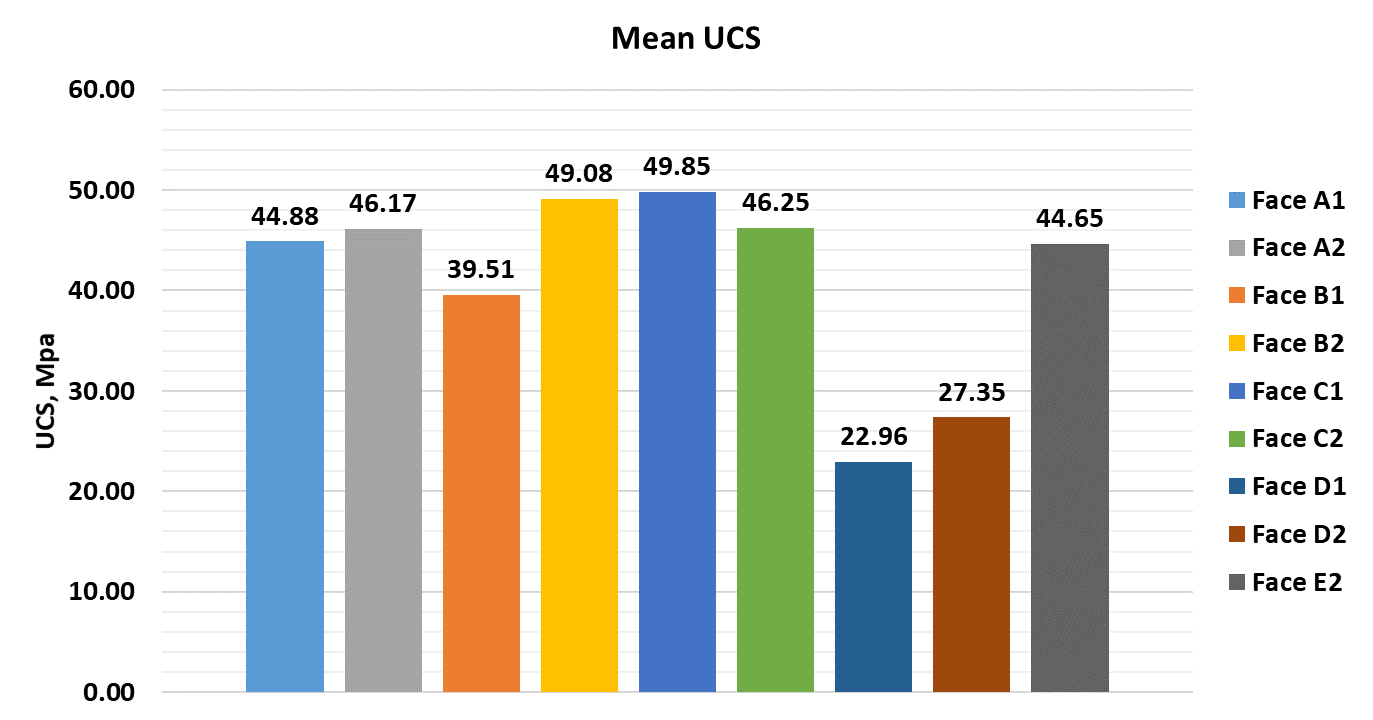


**Extended Data Figure 3 | Mean UCS values for each obelisk face**

*Figure 4. Minimum and maximum UCS values for each obelisk face*


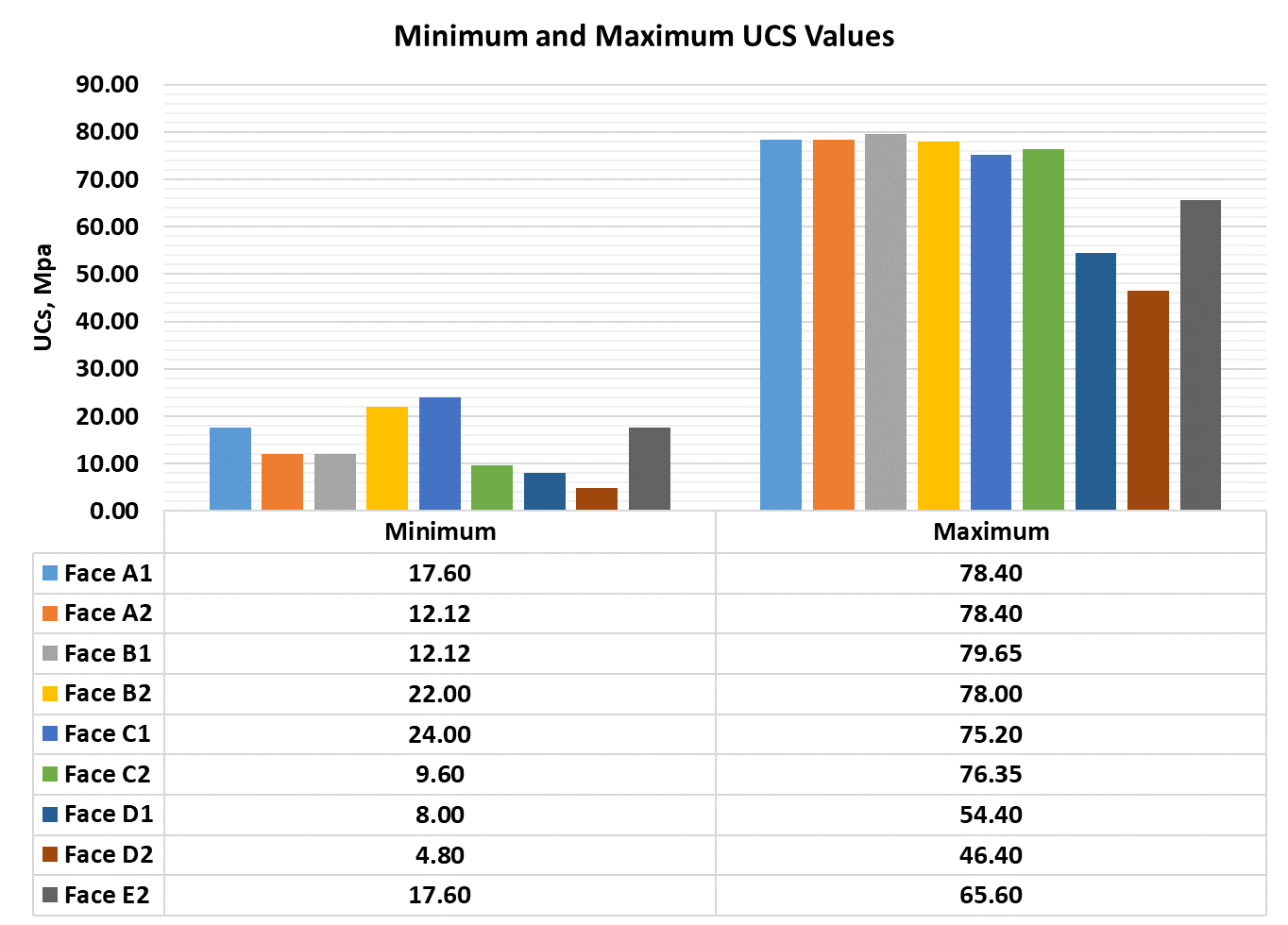


**Extended Data Figure 4 | Minimum and maximum UCS values for each obelisk face**


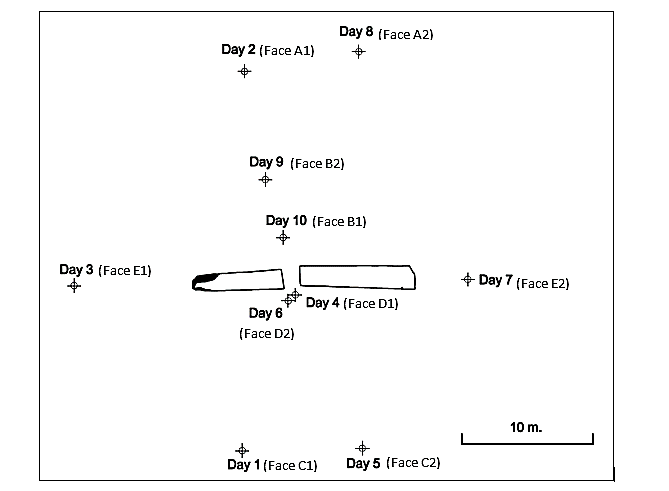


**Extended Data Figure 5 | Thermal camera positions.**

J


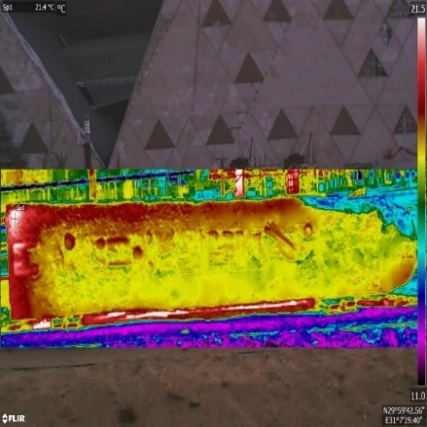


**P**

**Crack**

**S**


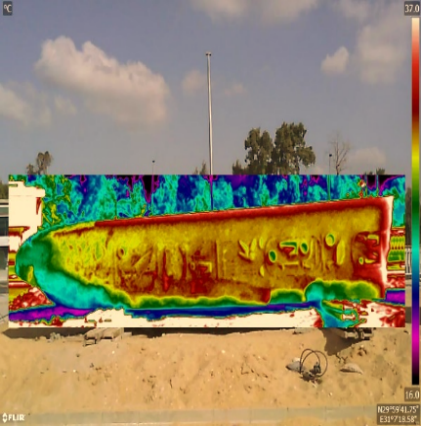


**Crack**

**Mineral heterogeneity**


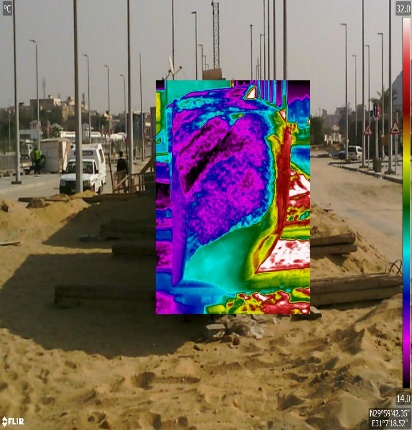


**Cracks**

(a) (b) (
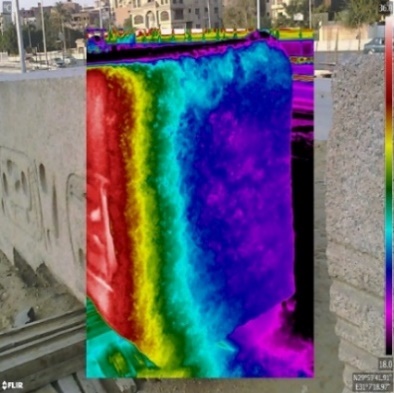
c)


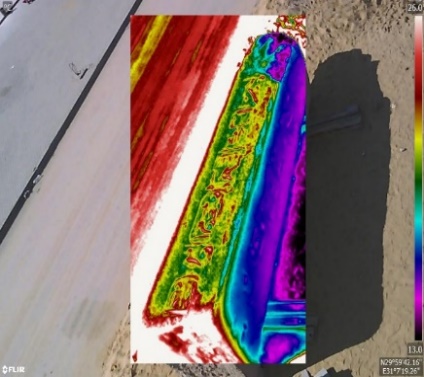


**Crack**


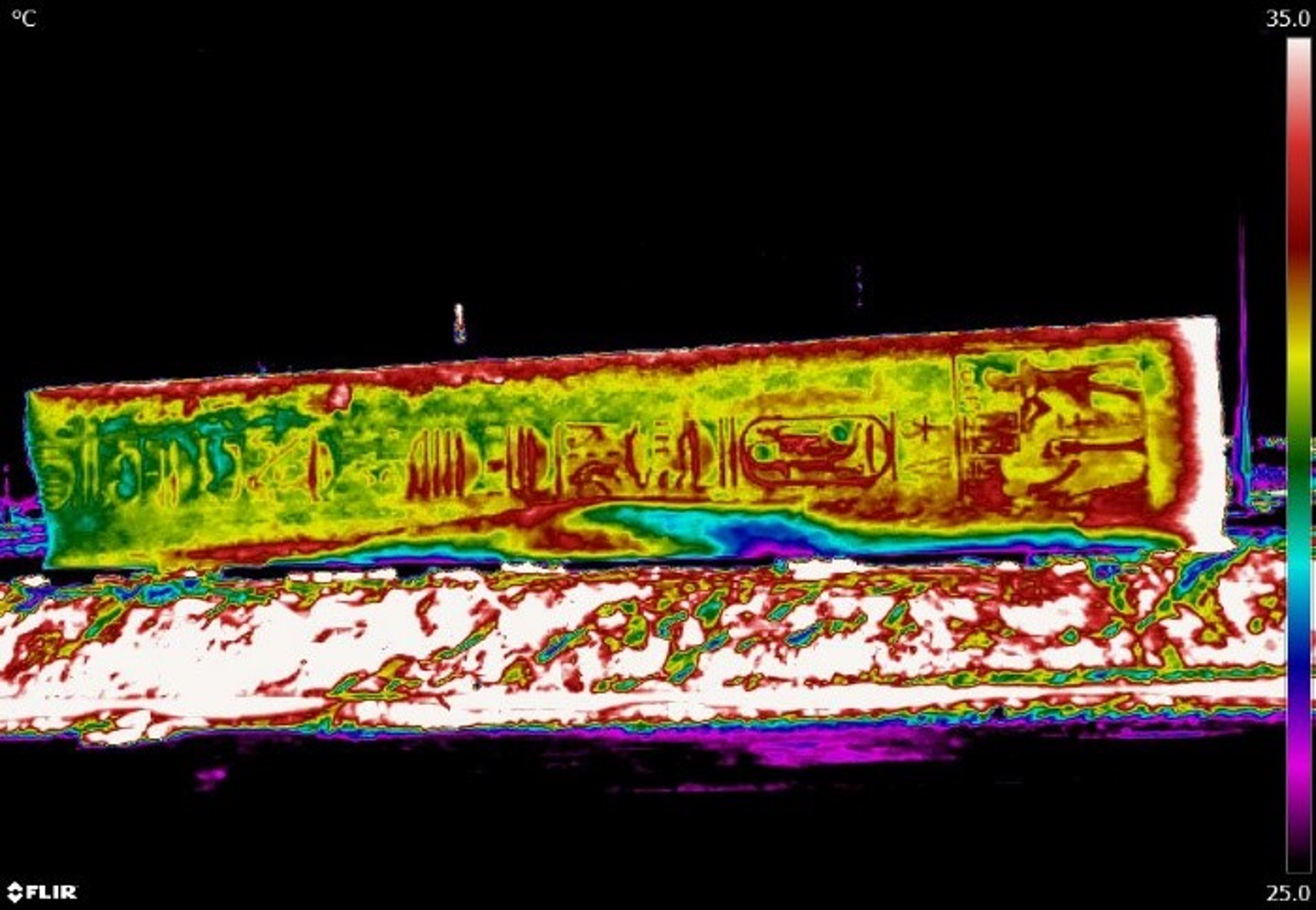


**Crack**

**Treated old Cracks**

(d) (e) (
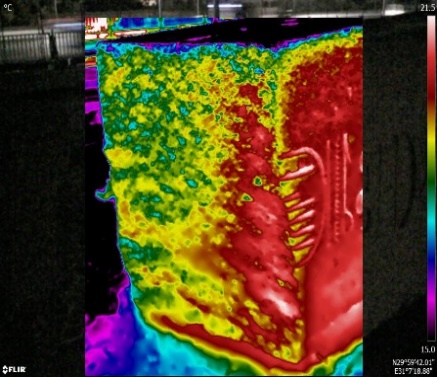

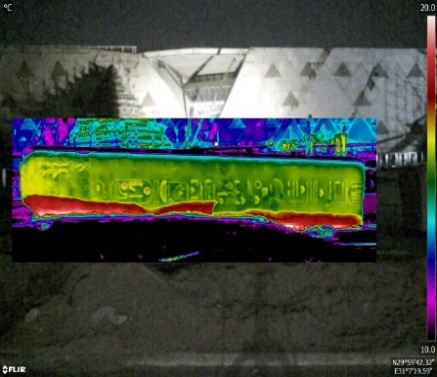
f)


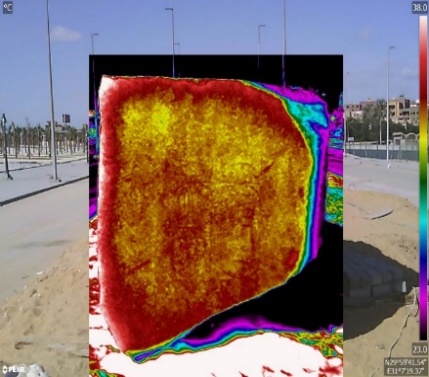


**Lenses or veins**

(g) (h) (i)


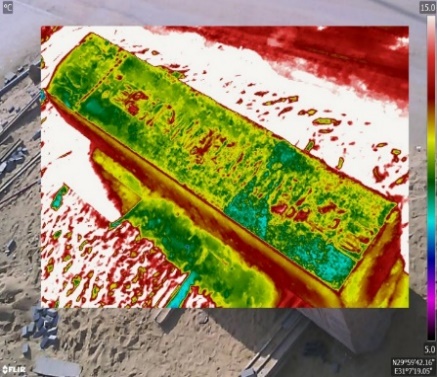


**Break lines**

(j)

**Extended Data Figure 6 *|* Thermal image of different faces with interpretation.** a, Face CI shows presence of crack and mineral heterogeneity. b, Face AI shows presence of crack. c, Face EI shows presence of cracks. d, Face DI. e, Face BI shows presence of crack. f, Face CII shows presence of crack and a zone of treated old cracks. g, face DII. h, Face EII shows presence of mafic lenses or xenoliths. i, Face AII. j, Face BII shows presence of break lines duo to different degrees of surface wear and polishing.

**Extended Data Figure 7 *|* Differences in heat gaining and releasing.**

**
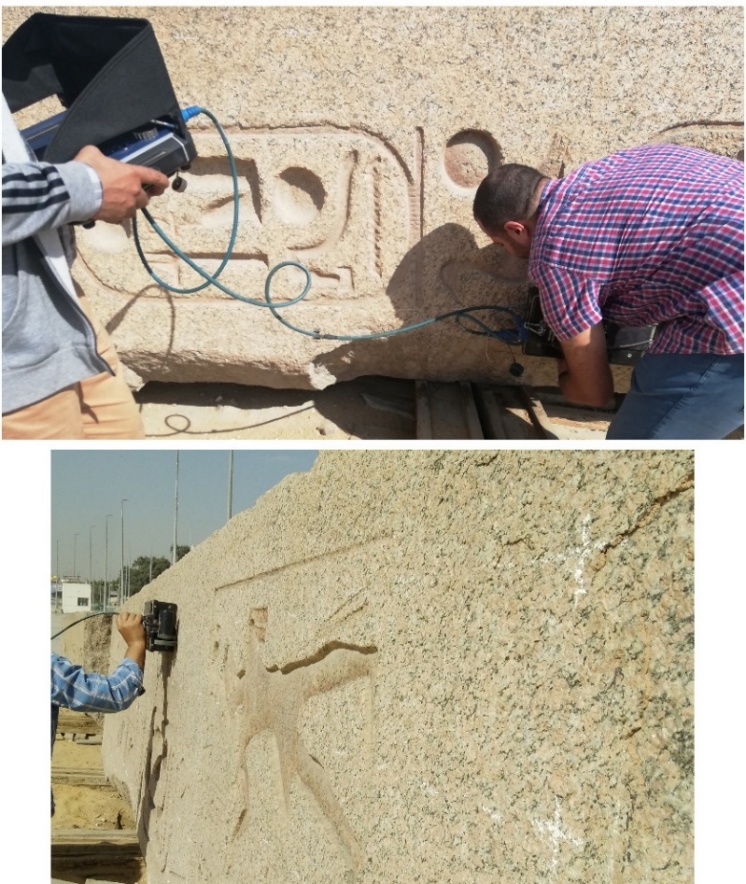
**

**Extended Data Figure 8 *|* GPR survey in part I (top) and part II (bottom).**

| 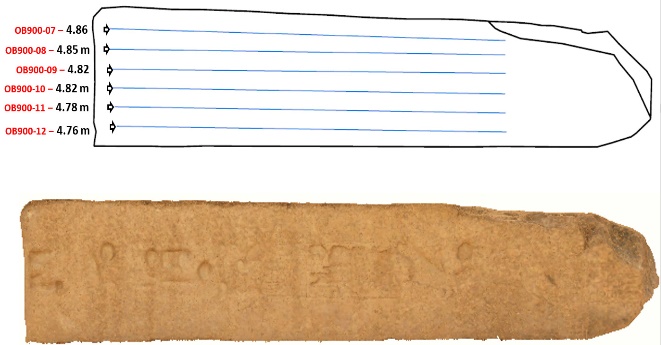  (a) | 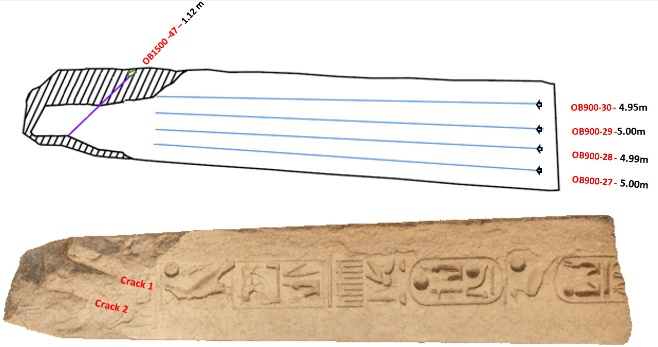  (b) |
| --- | --- |
| 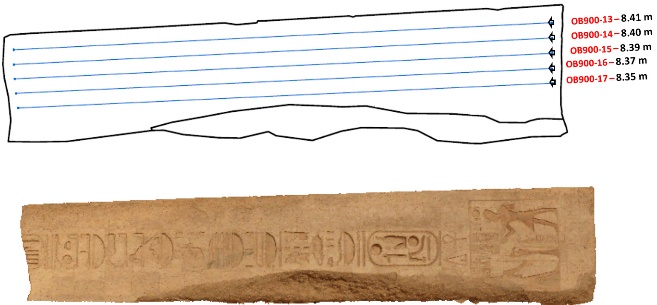  (c) | 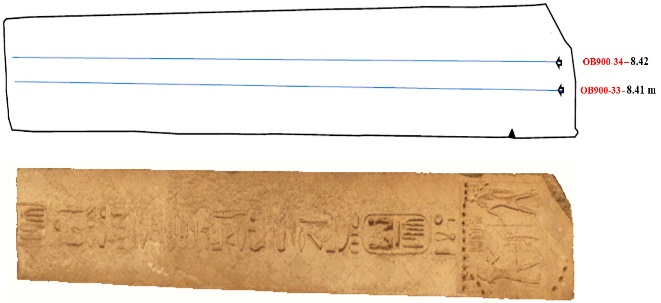  (d) |
| 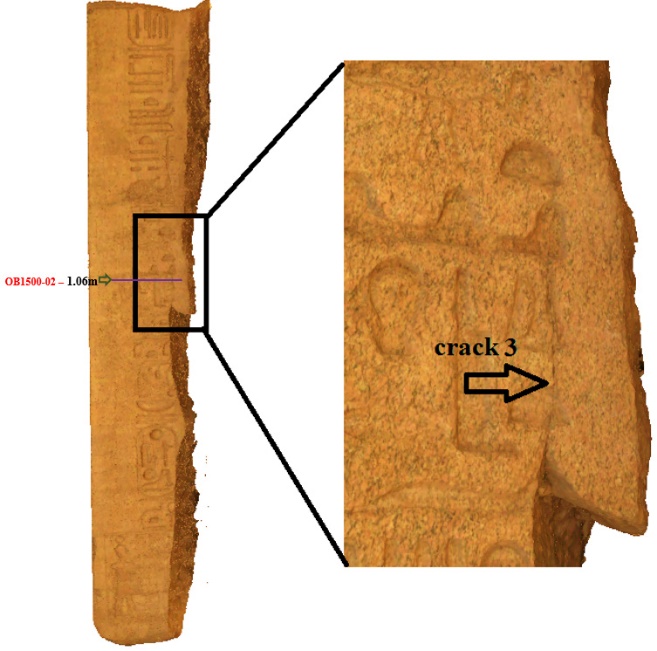  (e) | 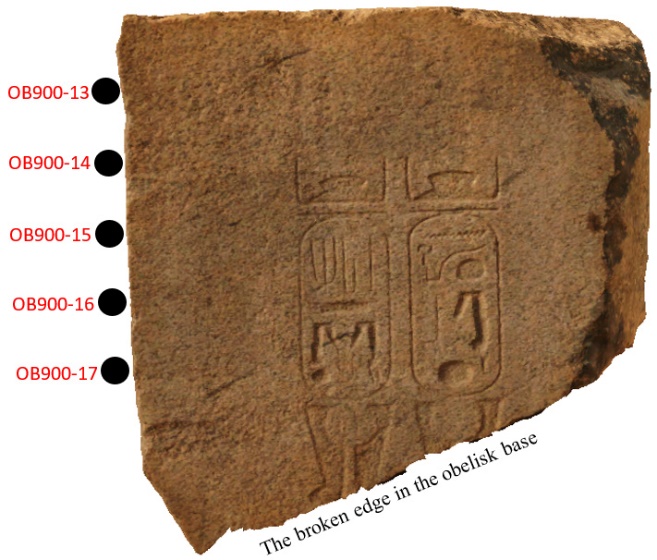  (f) |

**Extended Data Figure 9 |** **The layout of GPR measurements lines in the first round.** a, GPR Survey lines layout in face AI indicating the survey direction and lengths (top), the actual shape of face AI extracted from the constructed 3D model (bottom). b, Survey lines layout in face BI indicating the survey direction and lengths (top), the actual shape of BI extracted from the constructed 3D model (bottom). c, Survey lines layout in face CII indicating the survey direction and lengths (top), the actual shape of face CII extracted from the constructed 3D model (bottom). d, Survey lines layout in face BII indicating the survey direction and lengths (top), the actual shape of face BII extracted from the constructed 3D model (bottom). e, GPR survey line over Crack 3 in Face AII. f, The obelisk base and the position of the radargrams of face CII.

| 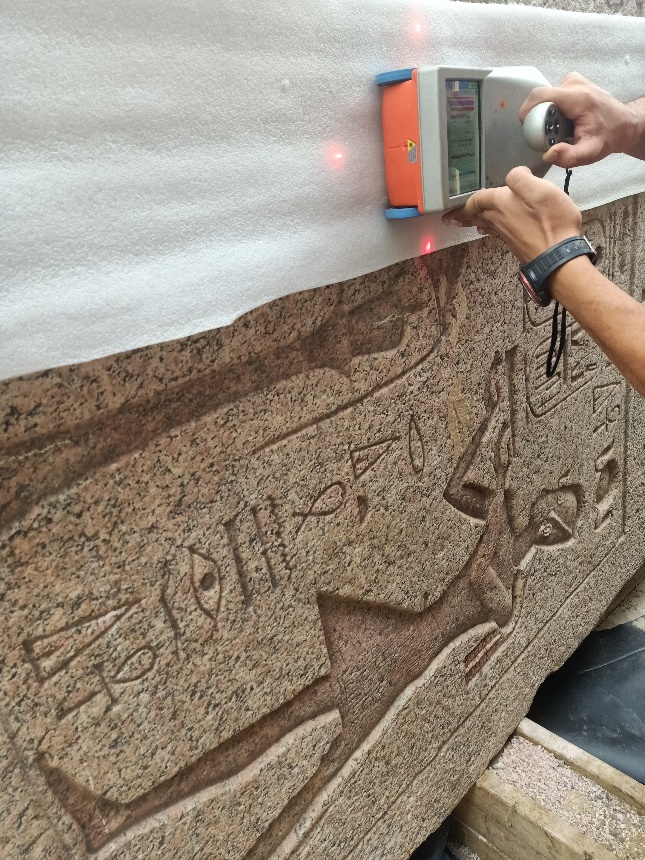  (a) | **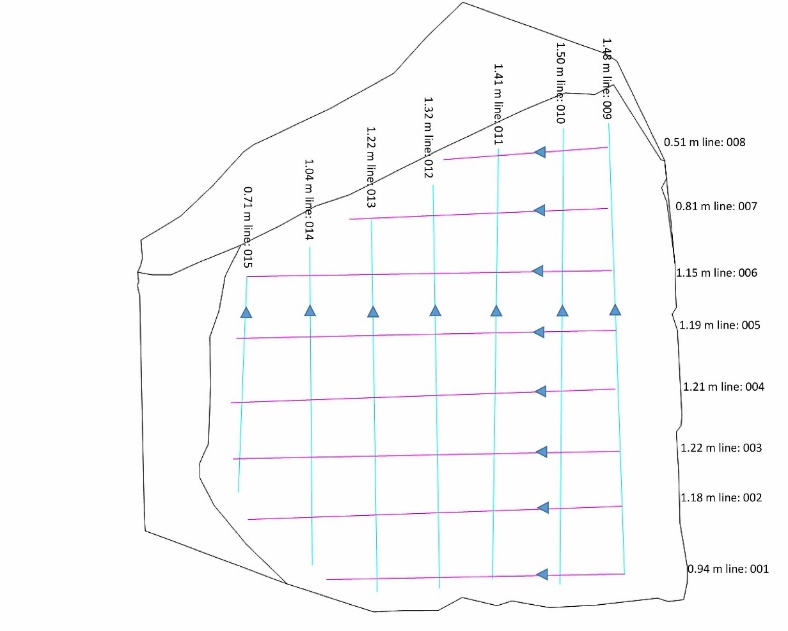**  (c) |
| --- | --- |
| 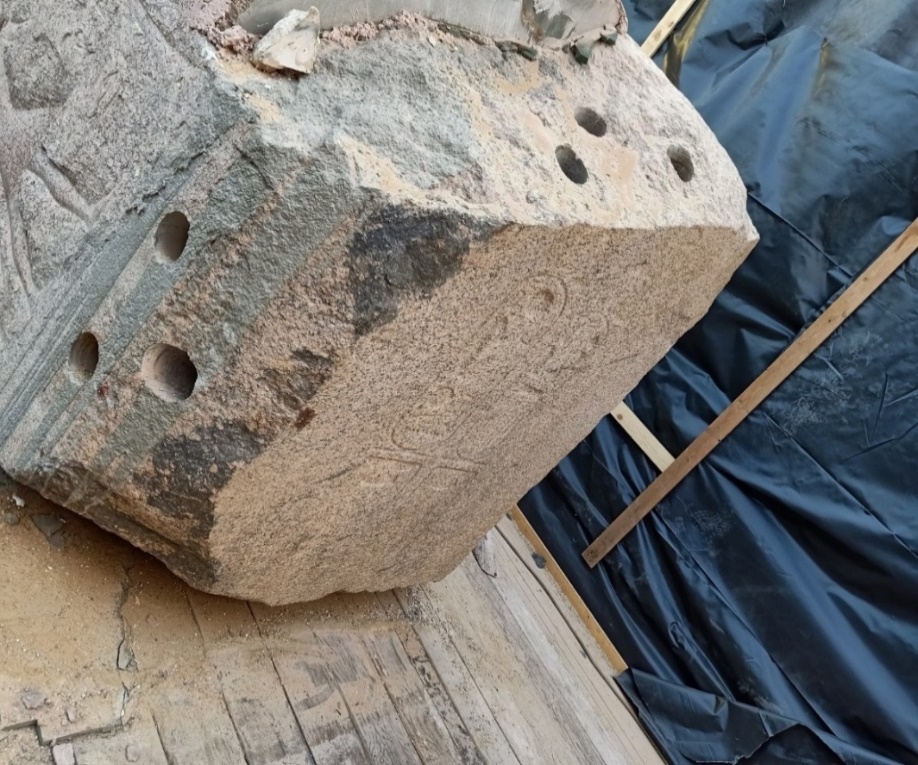  (b) | **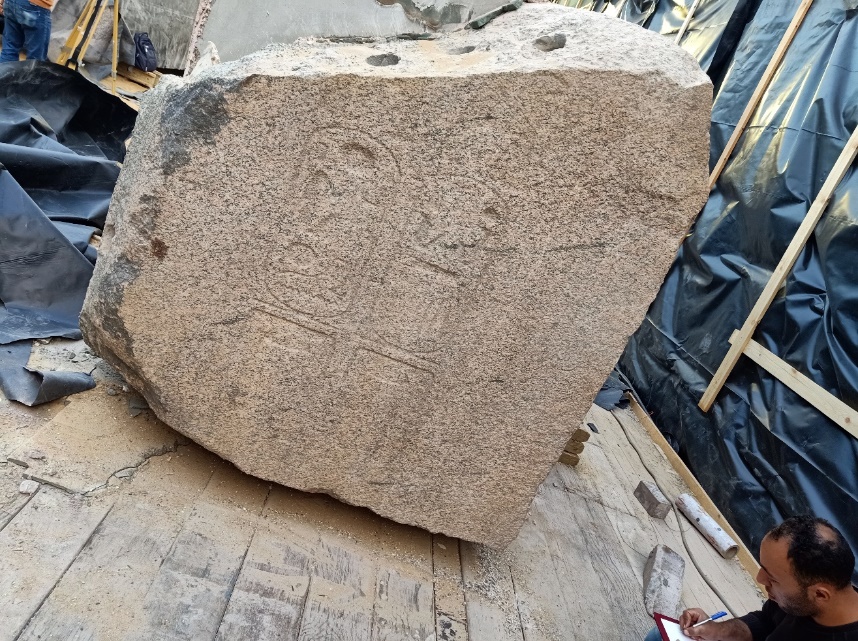**  (d) |

**Extended Data Figure 10 | The layout of GPR measurements lines in the second round.** a, The used GPR antenna. b, The obelisk base shows the drill holes on the sides of the base. c, The survey lines layout in the area indicates the survey direction and lengths. d, The actual picture of the area at the survey time.

| 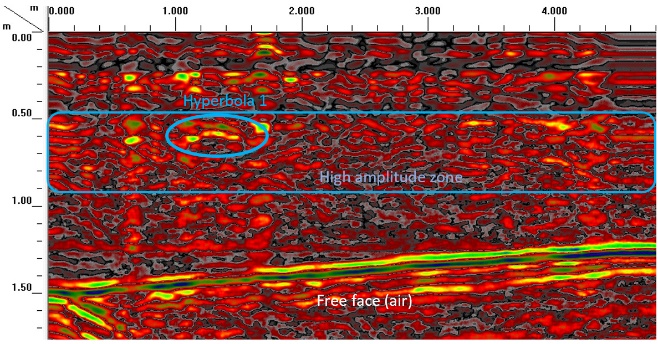  (a) | 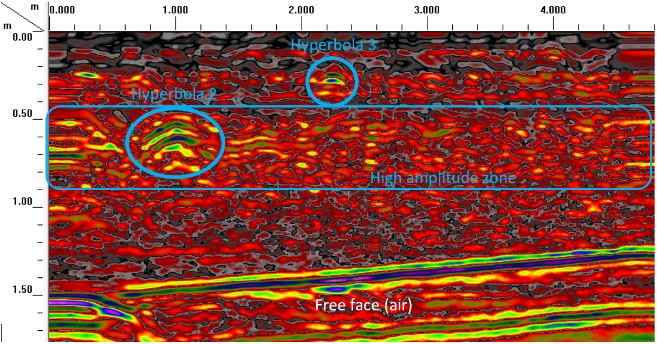  (b) |
| --- | --- |
| 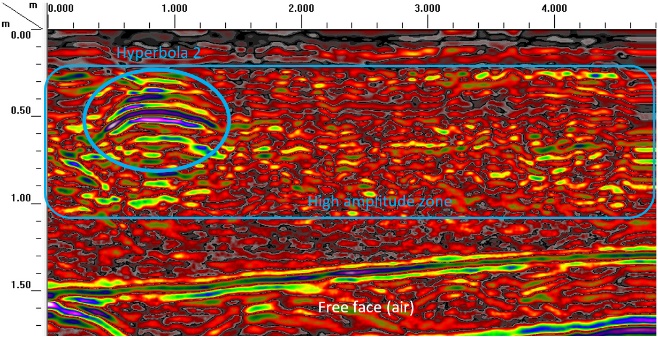  (c) | 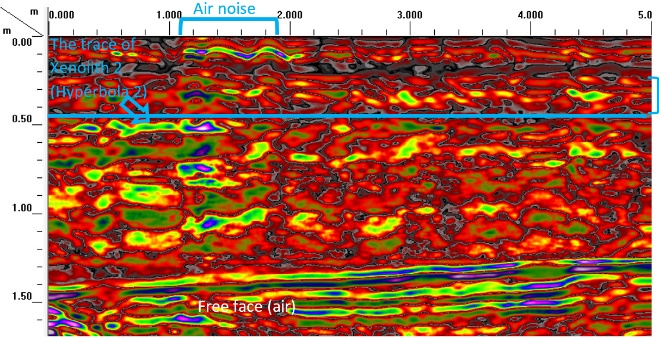  (d) |
| 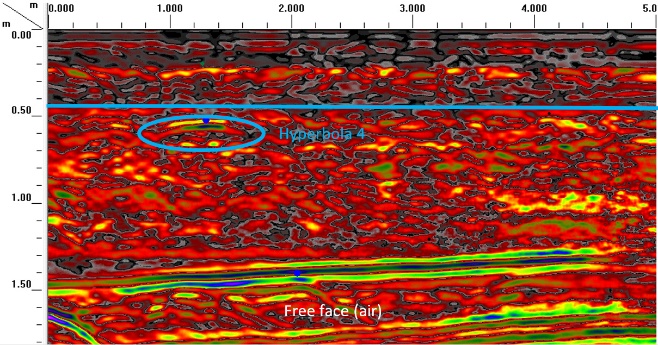  (e) | 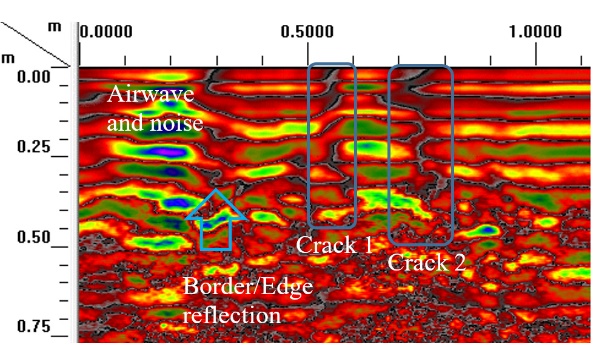  (f) |

**Extended Data Figure 11 | Interpreted radargrams of the first round measurements.**
a, Radaragram OB900-09. b, Radaragram OB900-11. c, Radaragram OB900-12.
 d, Radaragram OB900-29. e, Radaragram OB900-27. f, Radaragram OB1500-47.

| 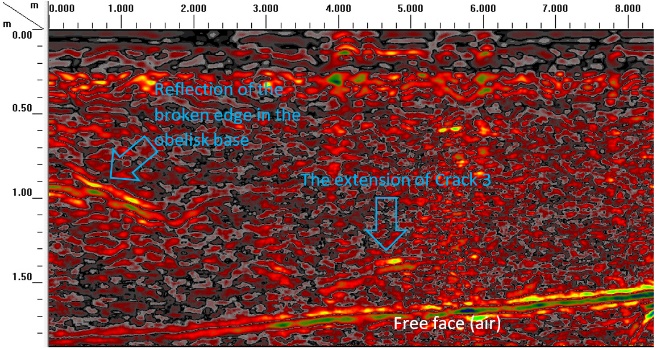  (a) | 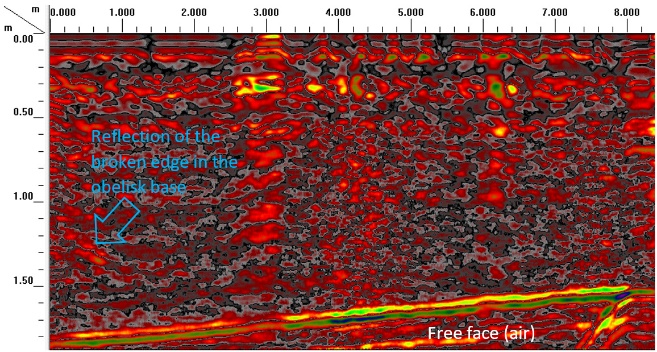  (b) |
| --- | --- |
| 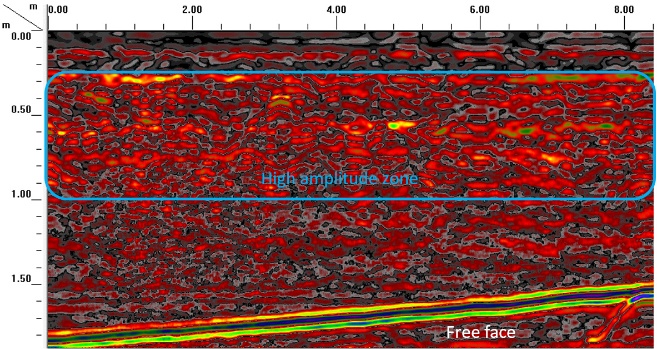  (c) | 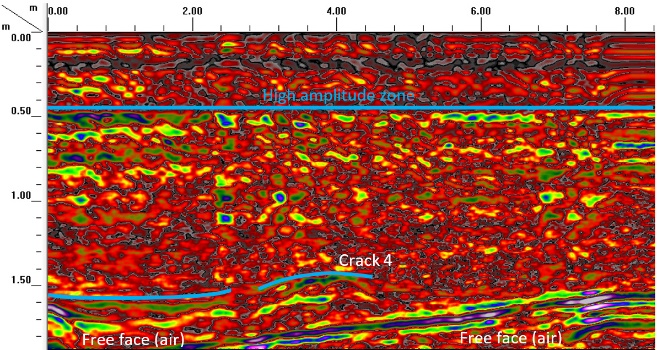  (d) |
| 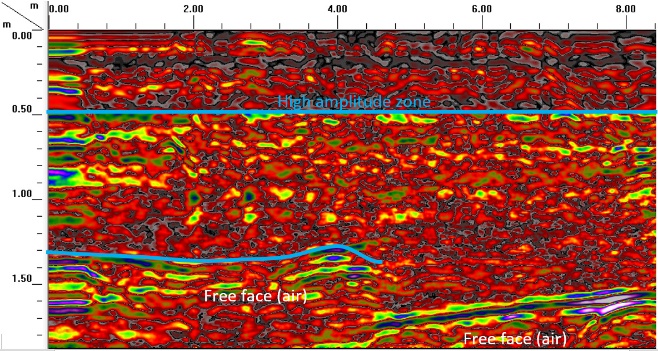  (e) | 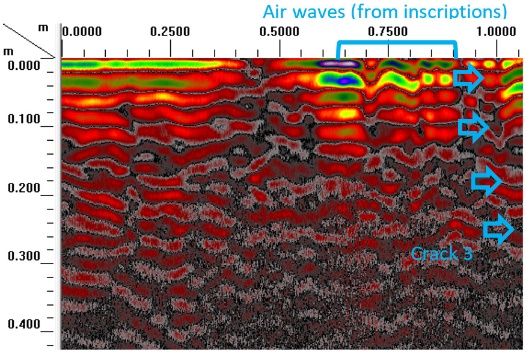  (f) |

**Extended Data Figure 12 | Interpreted radargrams of the first round measurements.**
a, Radargram OB900-17. b, Radargram OB900-15. c, Radargram OB900-13.
d, Radargram OB900-33. e, Radargram OB900-34. f, Radargram OB1500-02.

| 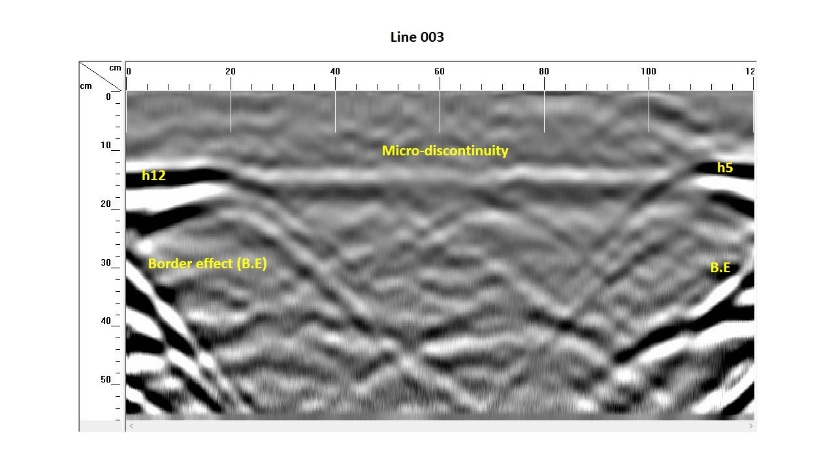  (a) | 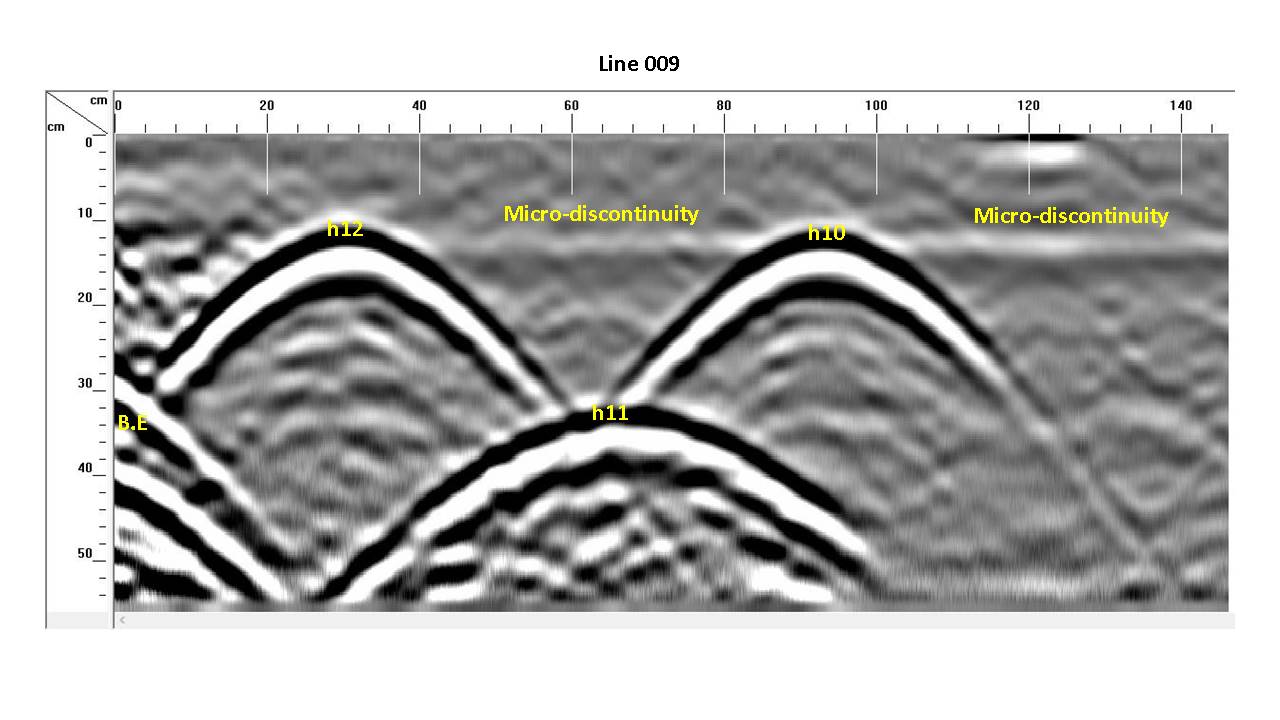  (d) |
| --- | --- |
| 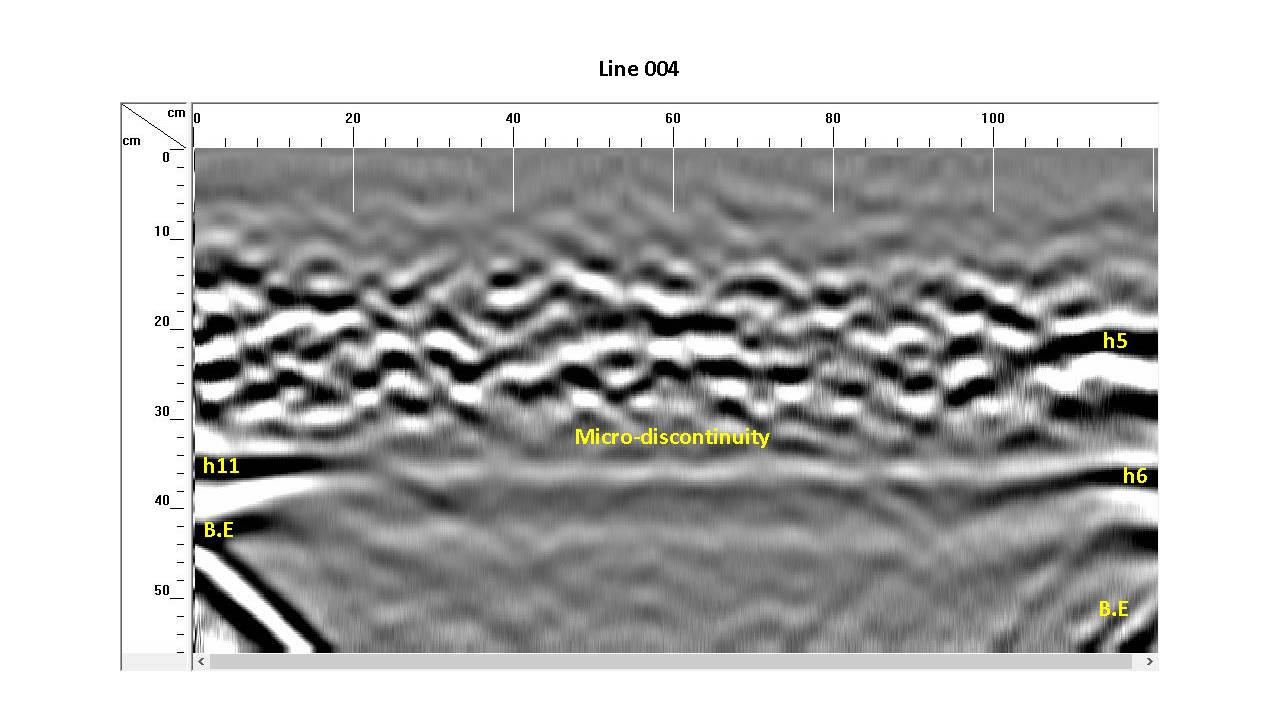  (b) | 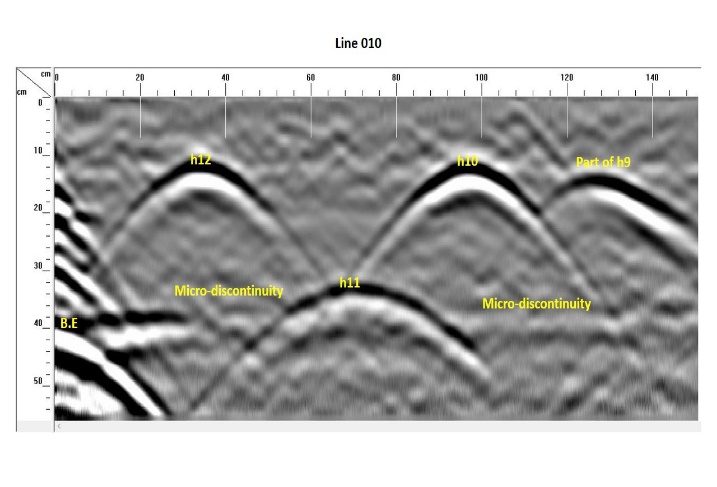  (e) |
| 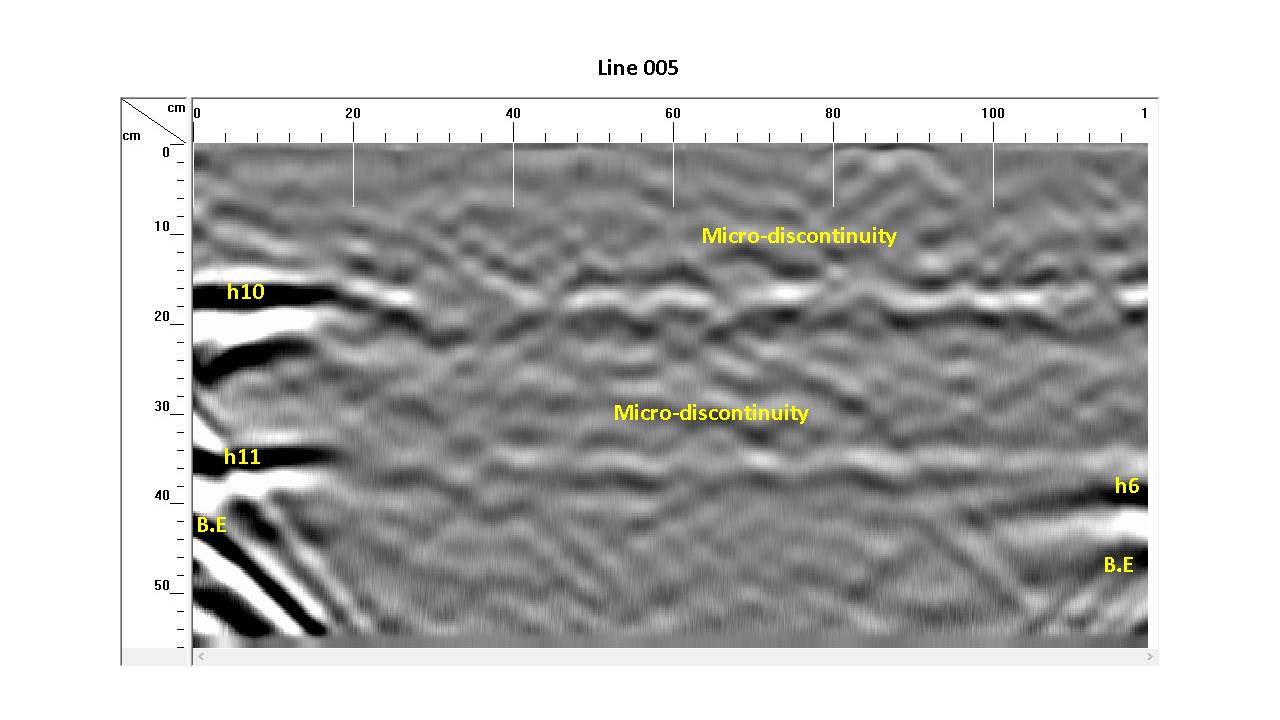  (c) | 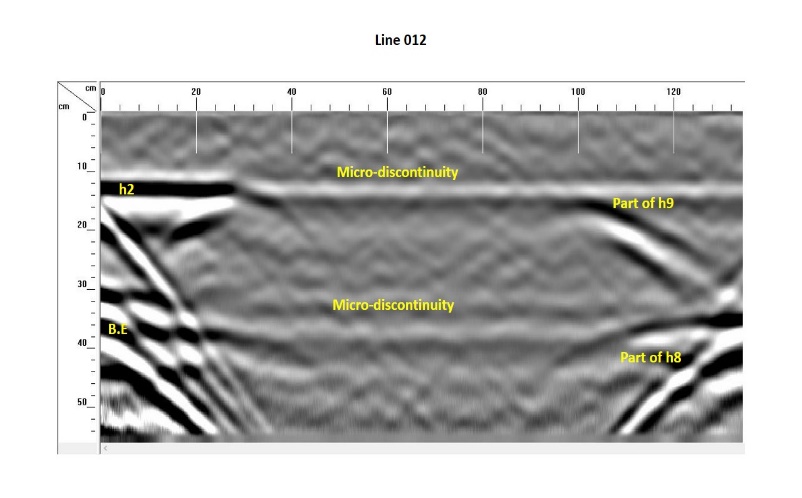  (f) |

**Extended Data Figure 13 | Interpreted radargrams of the second round measurements.**
a, Radargram 003, b, Radargram 004, and c, Radargram 005 are selected radargrams
from horizontal lines; while d, Radargram 009, e, Radargram 010, and f, Radargram 012
 are selected radargrams from vertical lines.


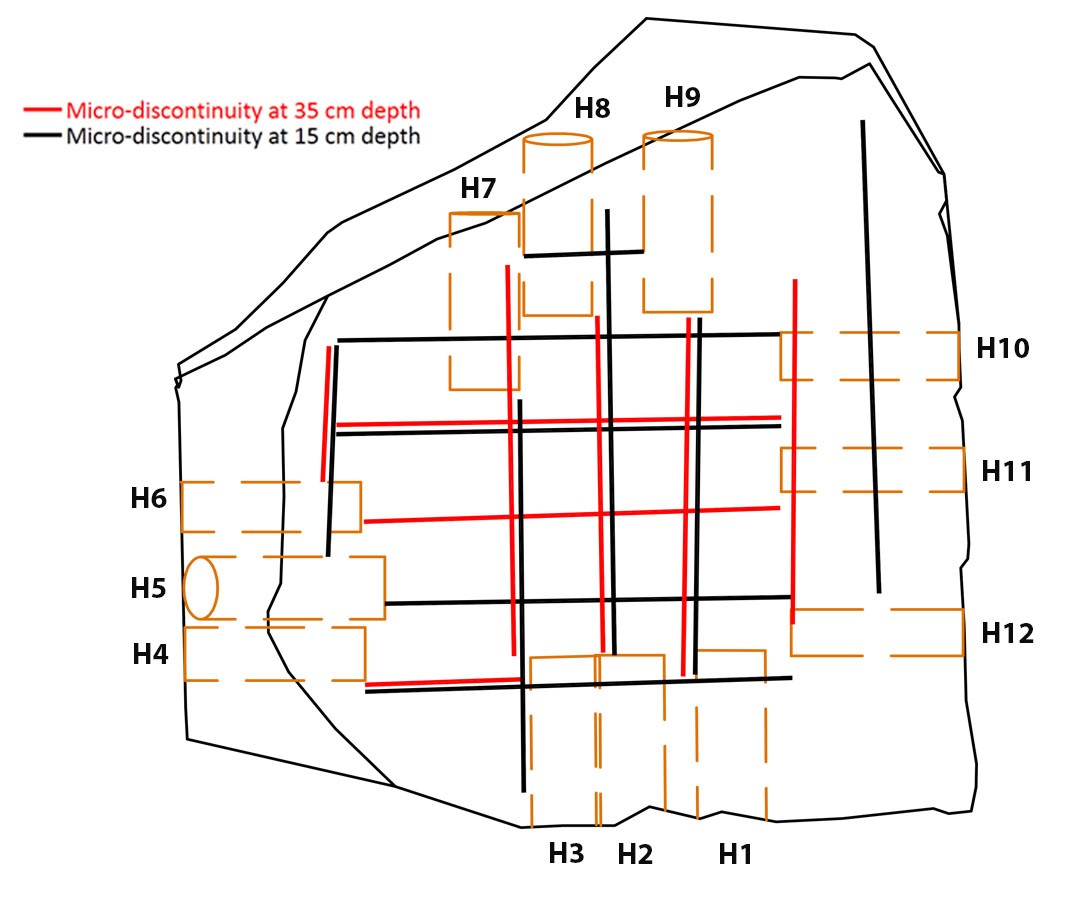


**Extended Data Figure 14 | The obelisk base with the interpreted networks of micro-discontinuities.**

| **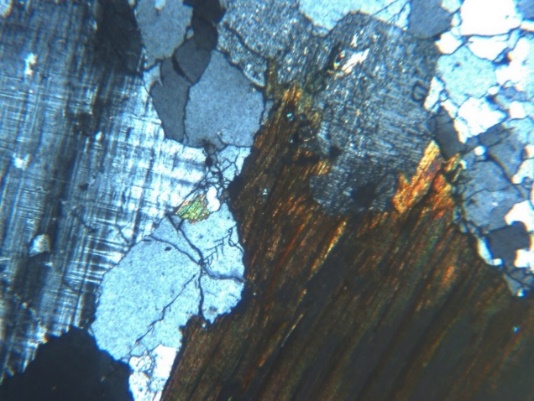**  (a) | **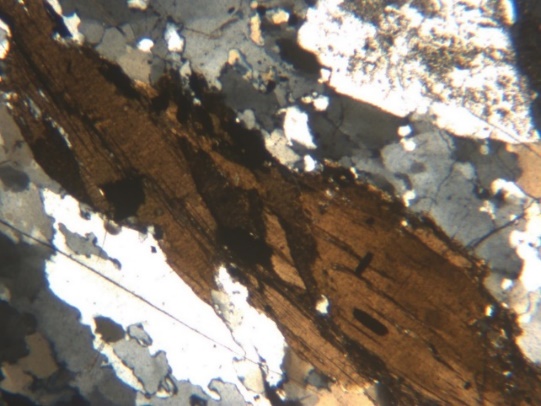**  (b) |
| --- | --- |

**Figure 15 | The samples under petrographic microscope.** a, Sample 1. b, Sample 2.

| 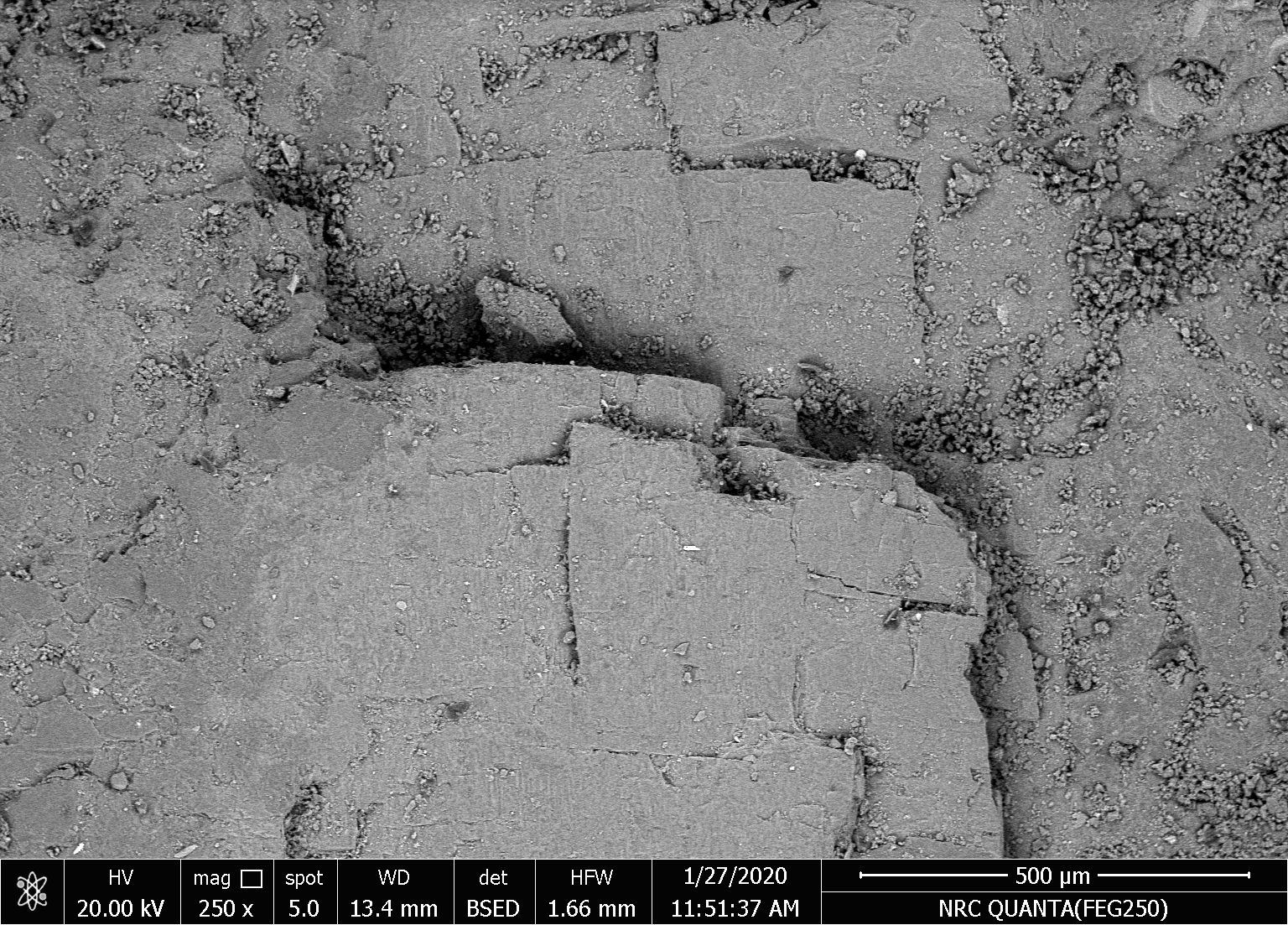 **Micro Crack**  **Intergranular corrosion** | 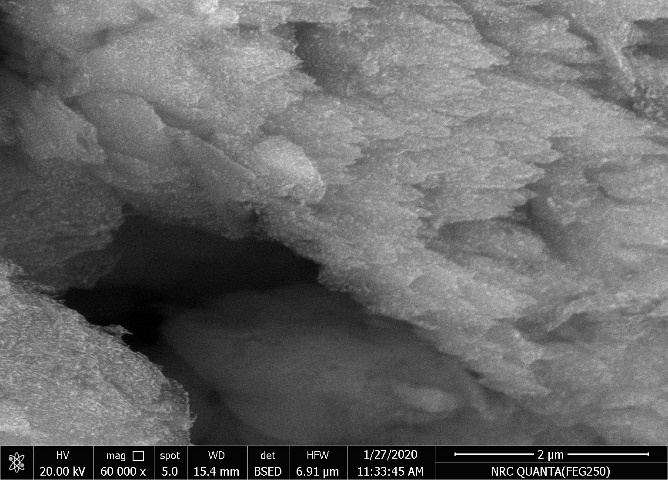 |
| --- | --- |
| (a) | (b) |

**Extended Data Figure 16 | SEM Analysis of the samples.** a, Sample 1. b, Sample 2.


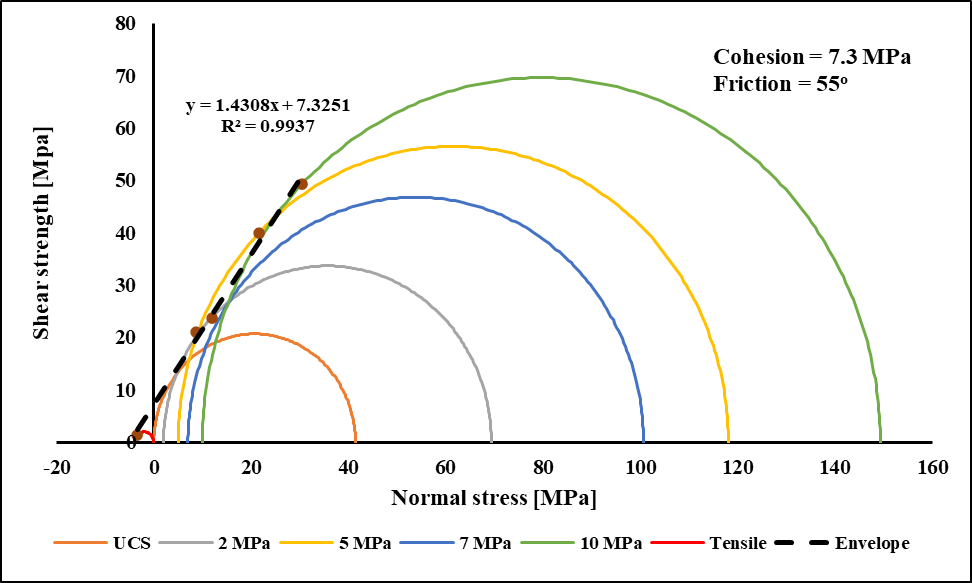


**Extended Data Figure 17 | Mohr–Coulomb failure envelope with tension cut-off, tensile failure is about 4 MPa. Compression is +ve.**


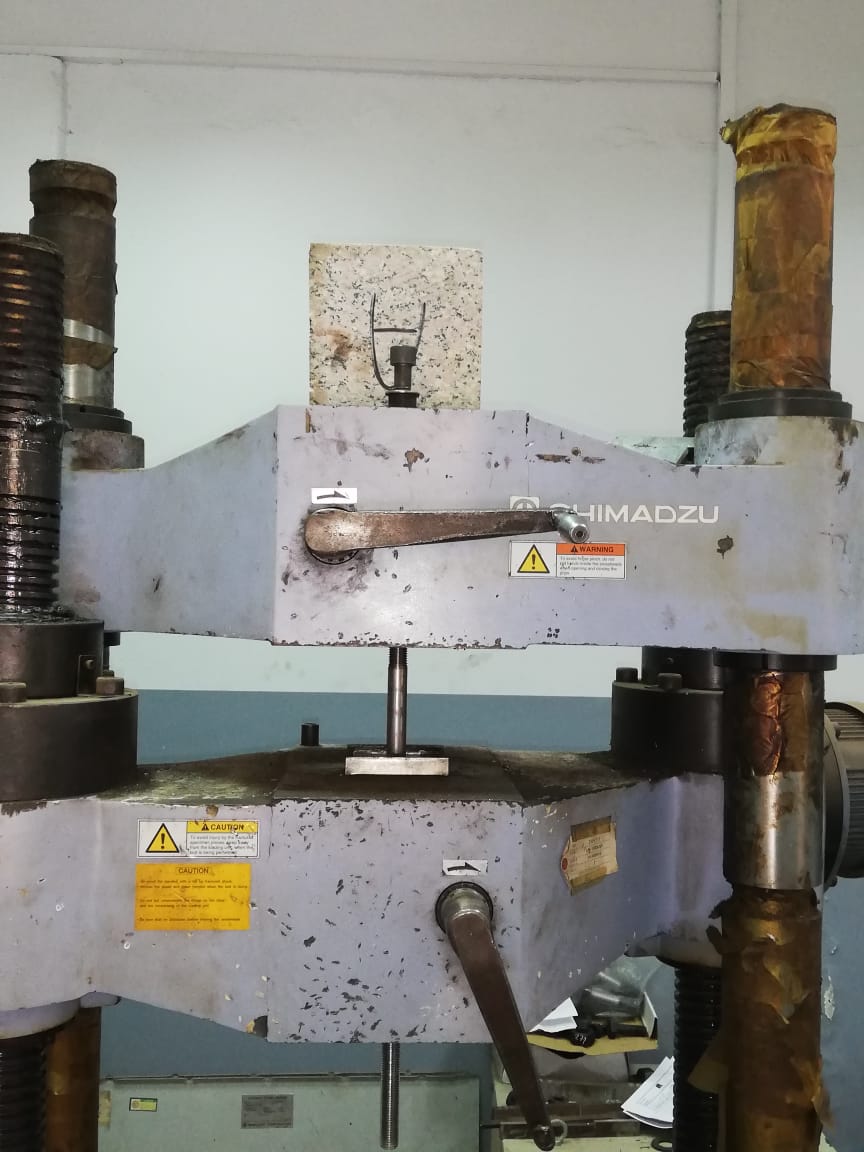

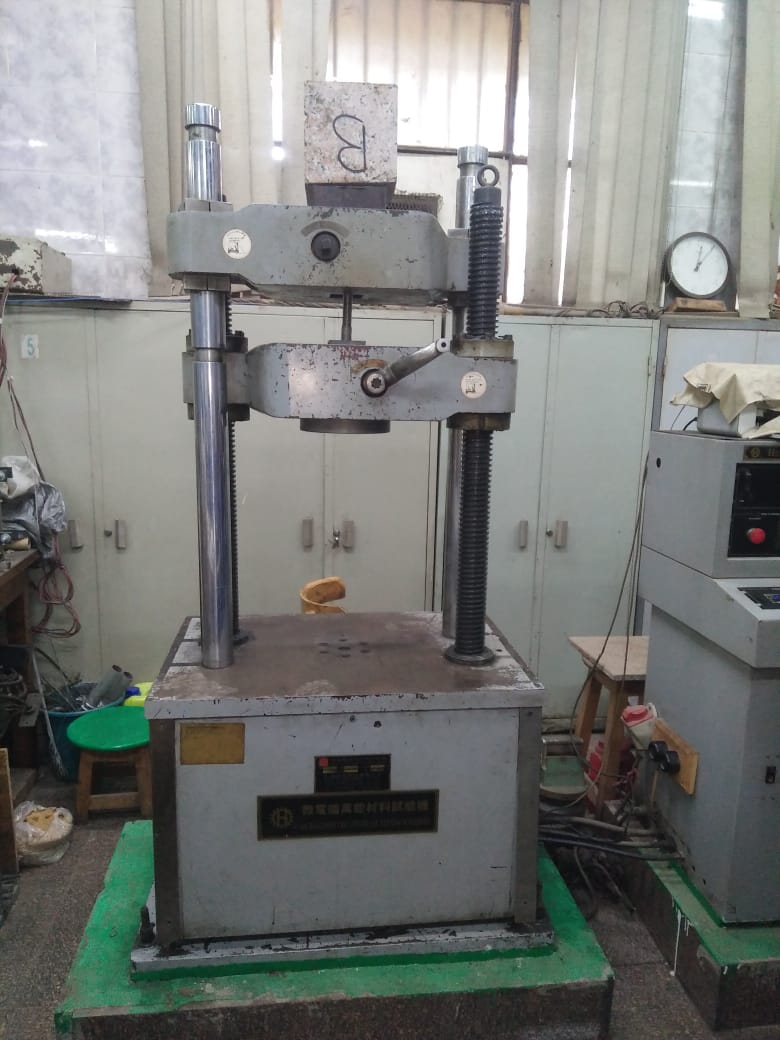

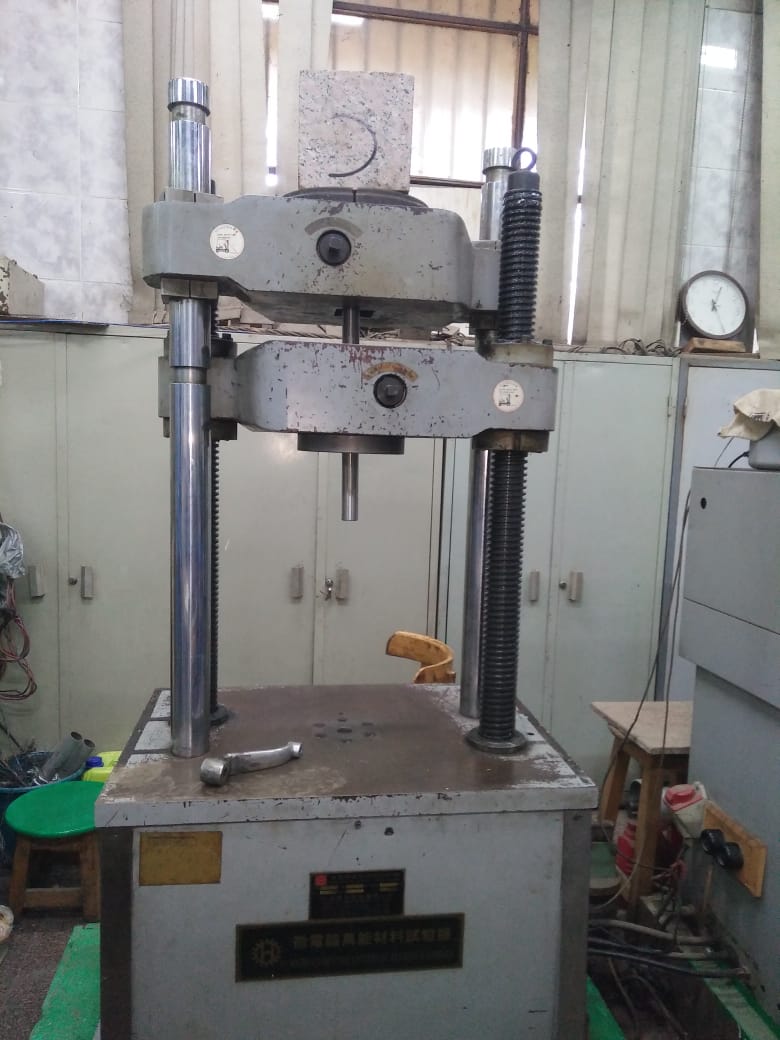

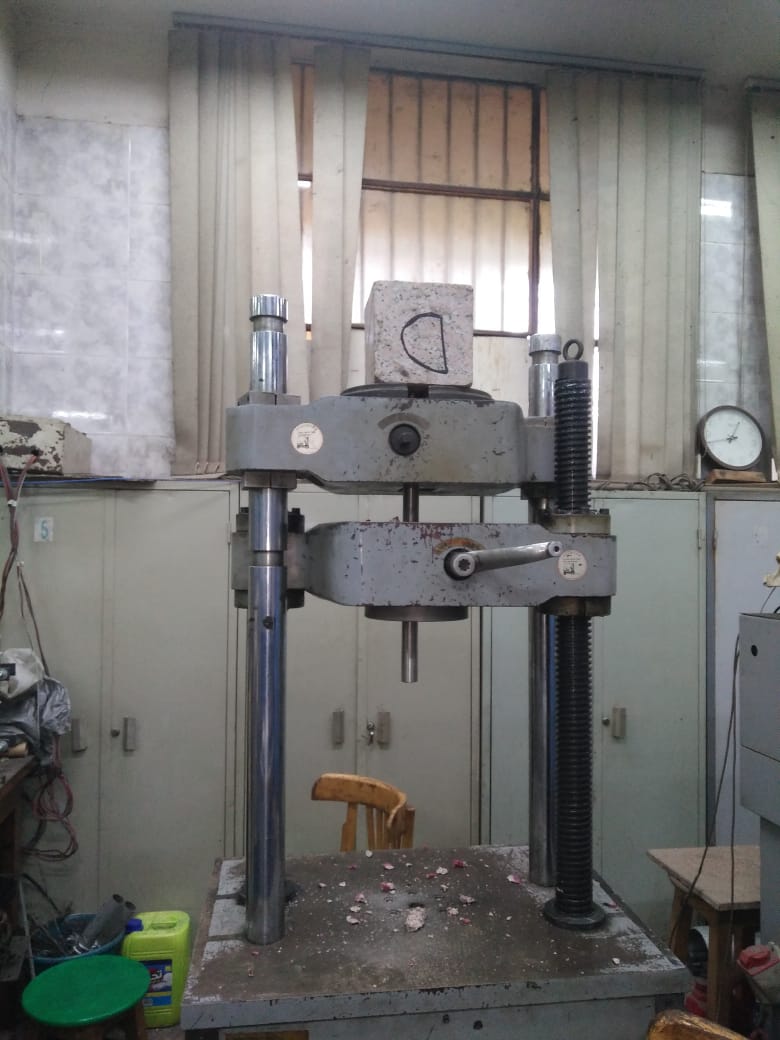


**Extended Data Figure 18 | Granitic rock samples.** (A, B, C and D) as installed in the pull-out test, the block was fixed on the top, while the anchor was pulled down.


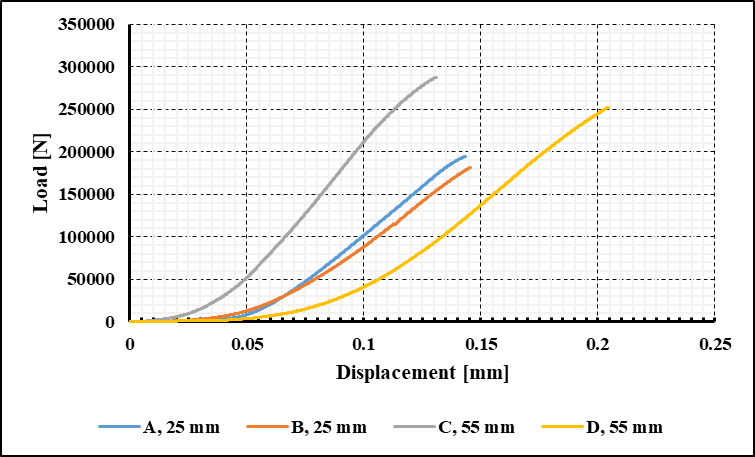


**Extended Data Figure 19 | Load – displacement curves for A, B, C, and D showing peak strength values.** The initial non-linearity in displacement could be explained due to many factors (such as: slippage of anchor, compression of pre-existing fractures, and movement of the loading rig among others)

| a | 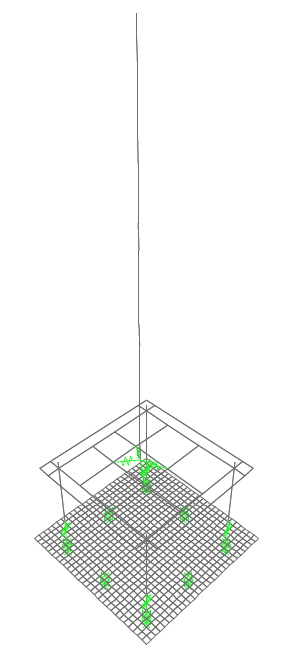  b |
| --- | --- |
| 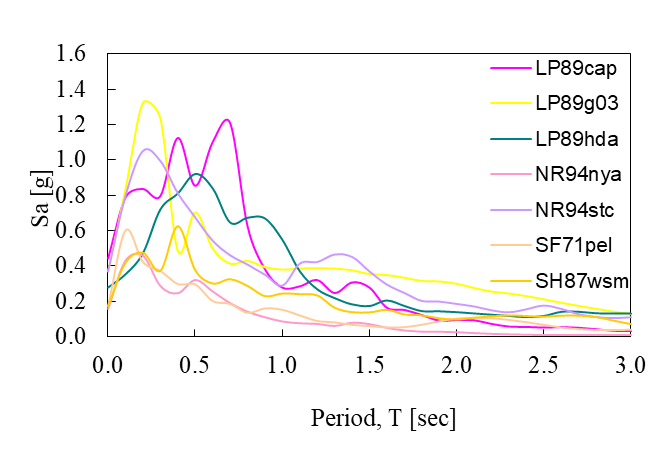  c | 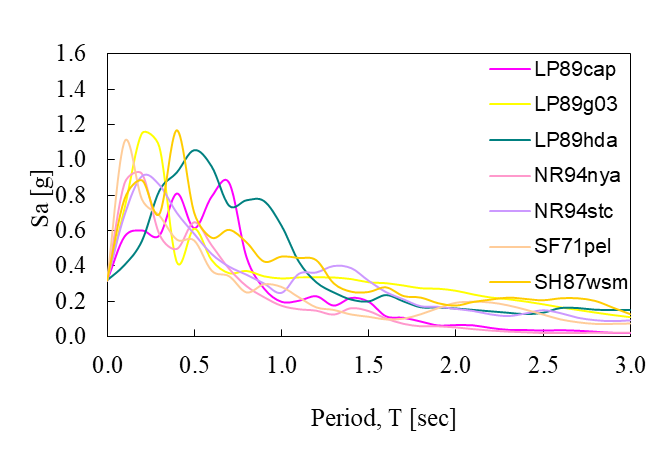  d |

**Extended Data Figure 20 | The elastomeric bearing analysis.** a, Arrangement of bearings under
the base. b, The developed finite element model. c, original response spectrum for the seven
records selected. d, scaled response spectrum for the selected records.


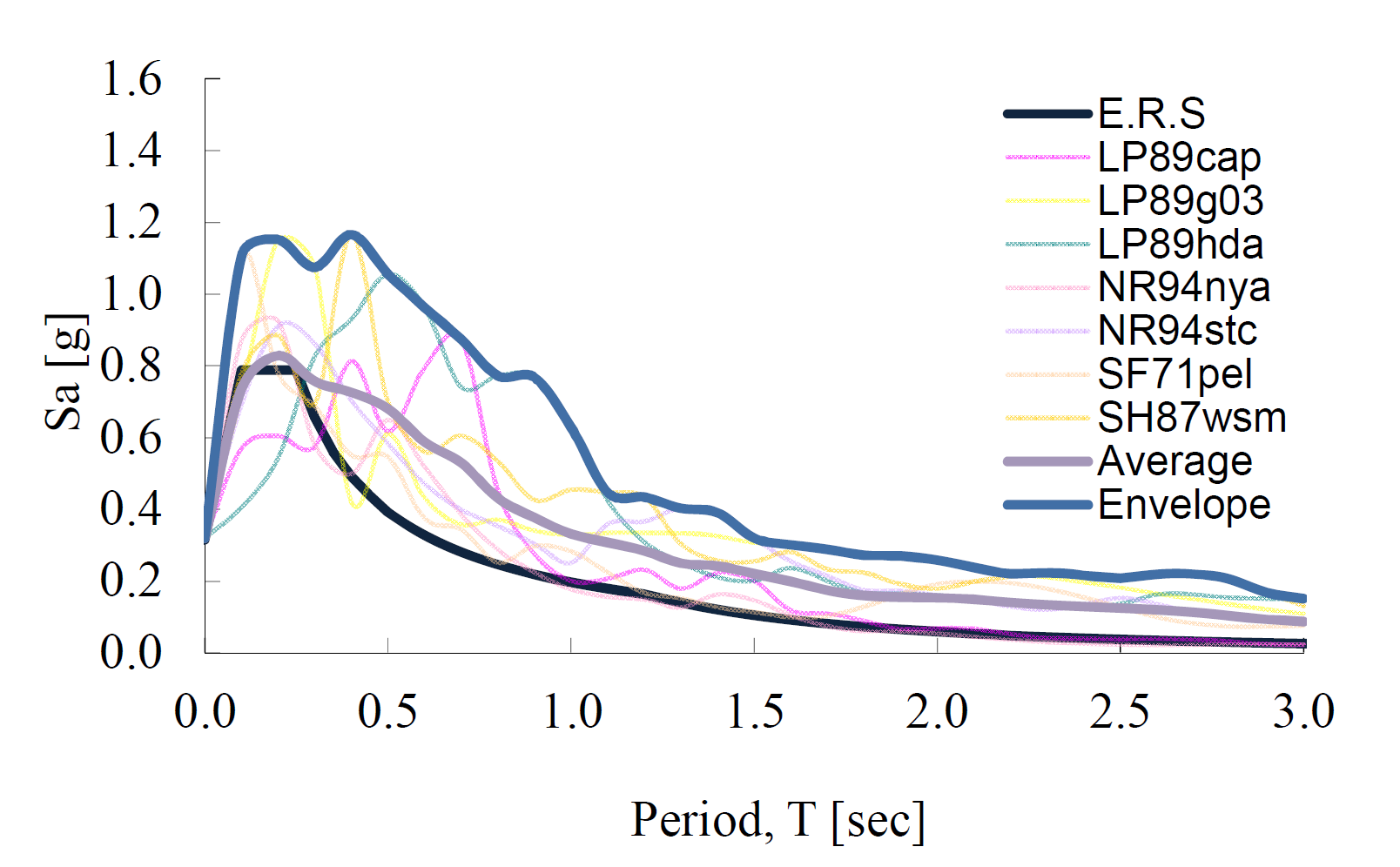


**Extended Data Figure 21 | Figure showing comparison between the Egyptian Code of Practice response spectrum (E.R.S) to the response spectrum of the seven selected earthquake records, including the average and the envelope values.**

| 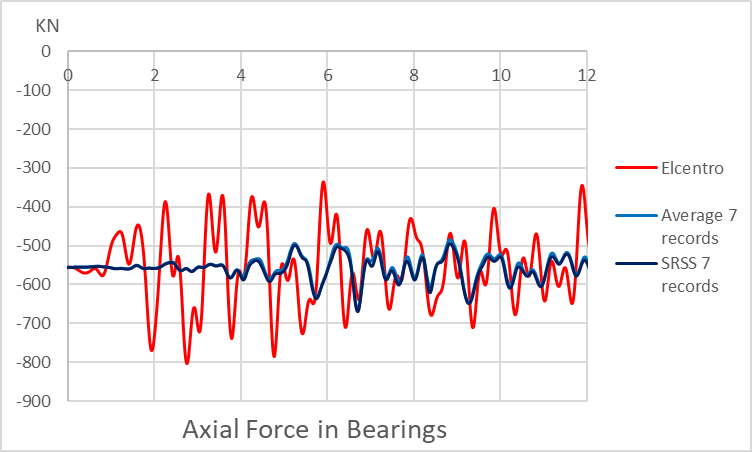 | 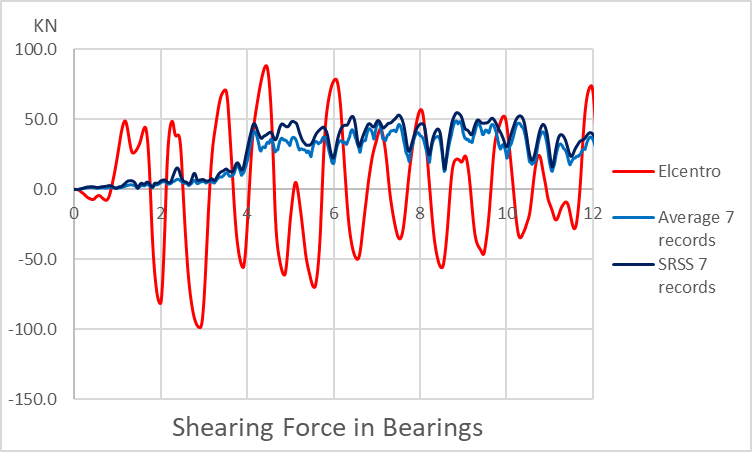 |
| --- | --- |
| a | b |
| 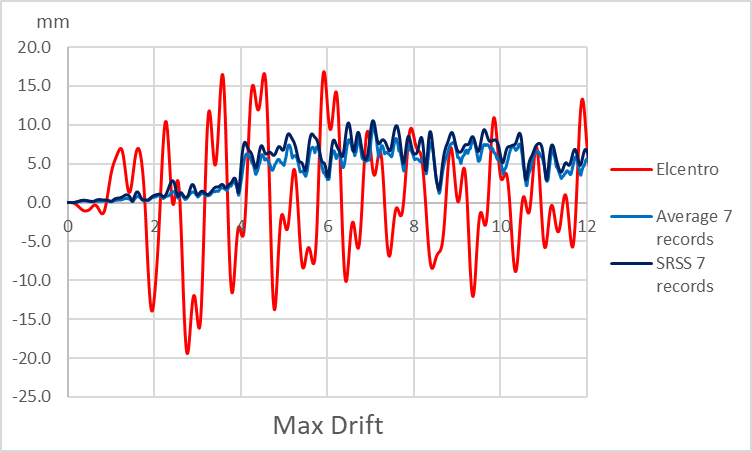 | 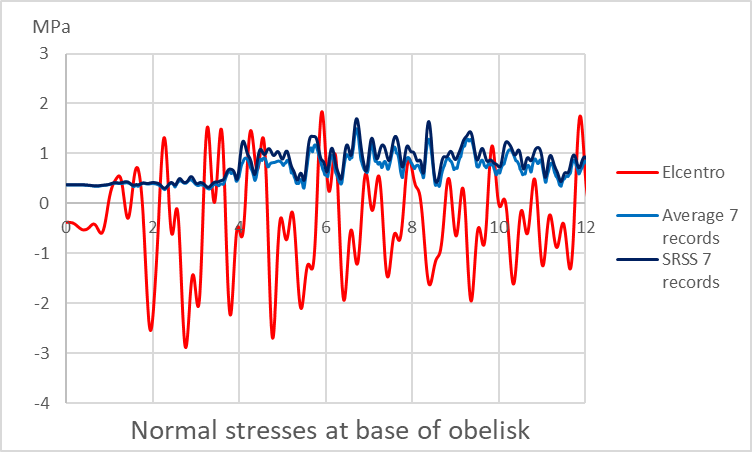 |
| c | d |
| 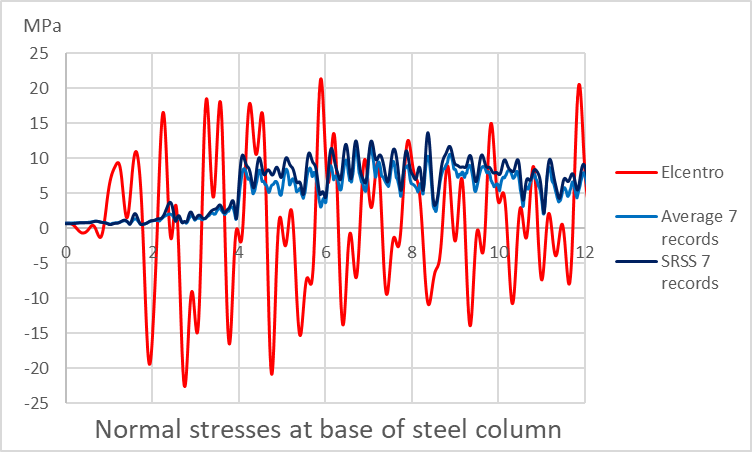 | 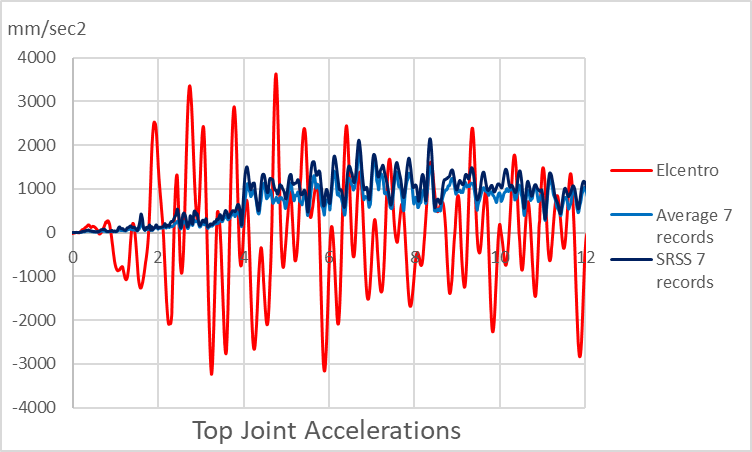 |
| e | f |

**Extended Data Figure 22 | Engineering demand parameters of the obelisk.**

| 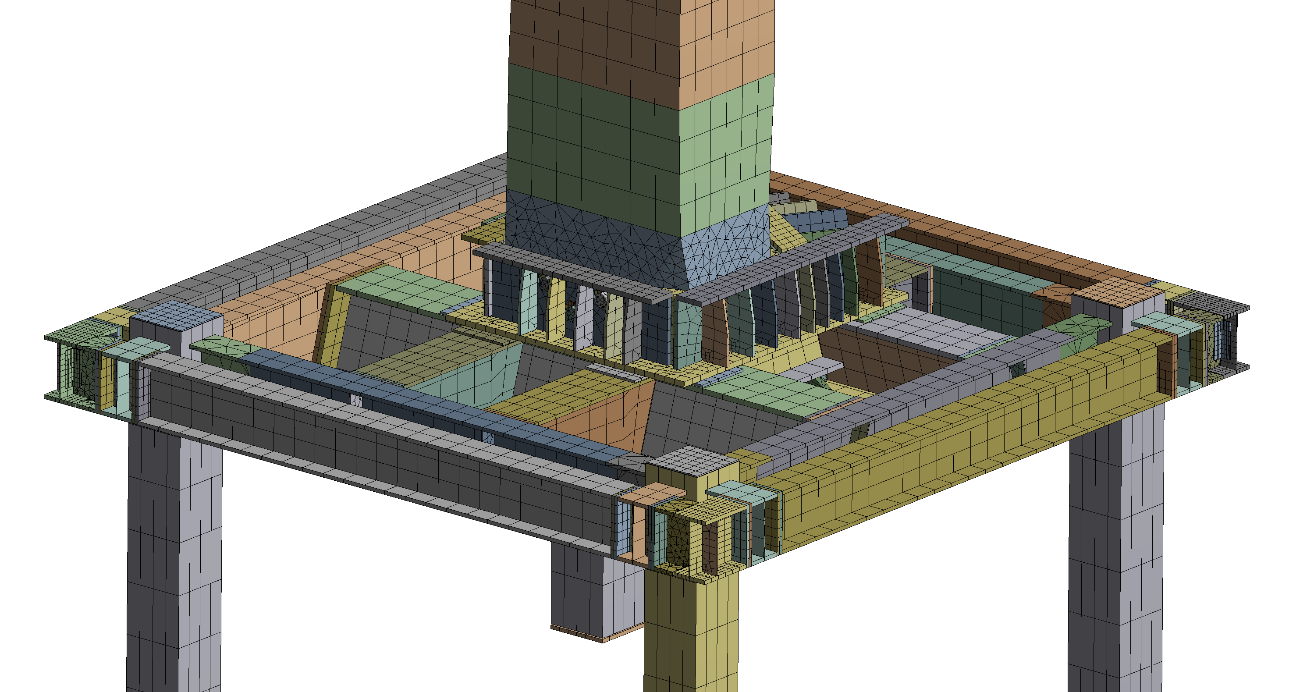(a) |
| --- |
| **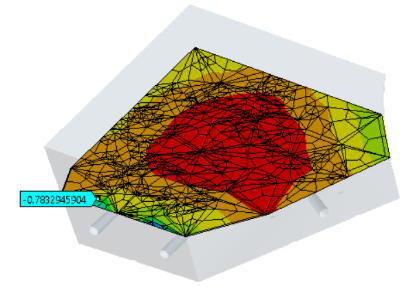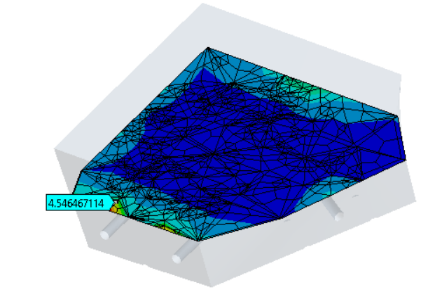**(b)  Stresses in granite due to lateral loads  Stresses in granite due to gravity loads |
| 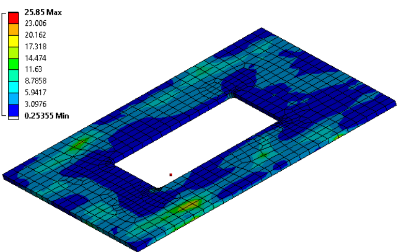(c) |
| (d)  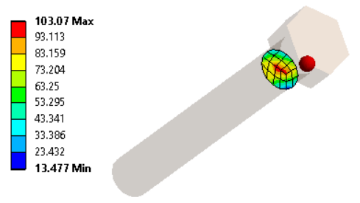 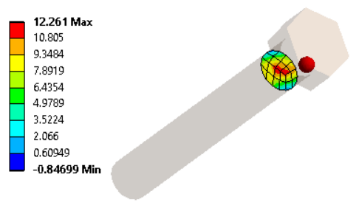  Stresses in anchor due to lateral loads  Stresses in anchor due to gravity loads |

**Extended Data Figure 23 | The 3D finite element model illustration.** a, Geometric illustration of the model. b, The stresses in the obelisk granite material due to gravity and lateral loads in MPa. c, The stresses in the top plate supporting the obelisk base in MPa. d, The stresses in the stainless-steel anchor due to gravity load and lateral loads in MPa.

**Extended Data Table 1 | Descriptive statistical analysis of the Schmidt hammer test results**

|  | **Face A1** | **Face A2** | **Face B1** | **Face B2** | **Face C1** | **Face C2** | **Face D1** | **Face D2** | **Face E2** |
| --- | --- | --- | --- | --- | --- | --- | --- | --- | --- |
| **Mean** | 44.88 | 46.17 | 39.51 | 49.08 | 49.85 | 46.25 | 22.96 | 27.35 | 44.65 |
| **Standard Deviation** | 14.71 | 12.88 | 18.05 | 14.41 | 11.21 | 14.49 | 12.04 | 13.67 | 11.80 |
| **Range** | 60.80 | 66.28 | 67.53 | 56.00 | 51.20 | 66.75 | 46.40 | 41.60 | 48.00 |
| **Minimum** | 17.60 | 12.12 | 12.12 | 22.00 | 24.00 | 9.60 | 8.00 | 4.80 | 17.60 |
| **Maximum** | 78.40 | 78.40 | 79.65 | 78.00 | 75.20 | 76.35 | 54.40 | 46.40 | 65.60 |
| **Count** | 57 | 90 | 38 | 45 | 80 | 96 | 20 | 35 | 22 |
| **Confidence Level (95.0%)** | ± 3.90 | ± 2.70 | ± 5.93 | ± 4.33 | ± 2.49 | ± 2.94 | ± 5.64 | ± 4.69 | ± 5.23 |

**Extended Data Table 2 | XRD results for Samples 1 and 2**

| **Sample 1** | | **Sample 2** | |
| --- | --- | --- | --- |
| **Mineral** | **SemiQuant [%]** | **Mineral** | **SemiQuant [%]** |
| **Quartz, syn** | 42 | **Quartz** | 35 |
| **Muscovite-2M1, heated** | 25 | **Muscovite-2M1** | 20 |
| **Kaolinite** | 1 | **Actinolite** | 4 |
| **Actinolite** | 1 | **Kaolinite** | 1 |
| **Calcite** | 1 | **Albite** | 40 |
| **Albite, calcian, ordered** | 30 |  |  |

**Extended Data Table 3 | XRF analysis results**

| **2** | **1** | **D.N** |
| --- | --- | --- |
| 73.97 | 73.06 | **SiO2** |
| 0.34 | 0.49 | **TiO2** |
| 13.26 | 13.28 | **Al2O3** |
| 2.73 | 3.62 | **Fe2O3** |
| 0.08 | 0.07 | **MnO** |
| 0.30 | 0.29 | **MgO** |
| 1.55 | 1.51 | **CaO** |
| 2.58 | 2.29 | **Na2O** |
| 4.41 | 4.78 | **K2O** |
| 0.07 | 0.08 | **P2O5** |
| 0.51 | 0.37 | **LOI** |

**Extended Data Table 4 | Samples specifications and preparation**

| **Block code** | **Edge of block (cm)** | **Nominal Bit diameter (mm)** | **Rod diameter (mm)** | **Embedment length (mm)** |
| --- | --- | --- | --- | --- |
| **A and B** | 20 | 54.74 | 25 | 150 |
| **C and D** |  | 75 | 55 | 150 |

**Extended Data Table 5 | Summary of test results for pull-out tests in granitic rock**

| **Item\Block** | **A** | **B** | **C** | **D** |
| --- | --- | --- | --- | --- |
| **h [mm]** | 150 | 150 | 150 | 150 |
| **d [mm]** | 25 | 25 | 55 | 55 |
| h /d | 6 | 6 | 2.7 | 2.7 |
| **N [KN]** | 194.5 | 181.4 | 287.6 | 252.5 |
| **τ_B_ [MPa]** | 16.5 | 15.4 | 11.1 | 9.74 |
| **Average τ_B_** | 15.95 | | 10.42 | |

**Extended Data Table 6 | The peak acceleration at top of the obelisk**

| **Record name** | **LP89cap** | **LP89g03** | **LP89hda** | **NR94nya** | **NR94stc** | **SF71pel** | **SH87wsm** | **Average** | **Elcentro** |
| --- | --- | --- | --- | --- | --- | --- | --- | --- | --- |
| **Peak acceleration** | 3240 | 2570 | 5010 | 2640 | 3510 | 3020 | 3500 | 3556 | 3630 |
